# Heterogeneity and multi-scale dynamics in the molecular bearing of the bacterial flagellum

**DOI:** 10.1038/s41467-026-74079-9

**Published:** 2026-06-12

**Authors:** Martin Rieu, Daping Xu, Gunasekaran Subramaniam, Ashley L. Nord, Alexis Courbet, Hafez El Sayyed, Richard M. Berry

**Affiliations:** 1 Department of Physics, University of Oxford, Oxford, UK; 2 Kavli Institute for Institute for Nanoscience Discovery, Oxford, UK; 3 Centre de Biologie Structurale, Université de Montpellier, CNRS, INSERM, Montpellier, France; 4 Institute for Protein Design, University of Washington, Seattle, WA USA; 5 Department of Biochemistry, University of Washington, Seattle, WA USA; 6 Howard Hughes Medical Institute, University of Washington, Seattle, WA USA

**Keywords:** Single-molecule biophysics, Proteins, Molecular modelling, Kinetics

## Abstract

The bacterial flagellum is a protein-based rotary machine that drives bacterial motility. It comprises the bacterial flagellar motor (BFM), consisting of a stator which is anchored to the cell wall and a rotor in the cytoplasmic membrane, linked via the flagellar rod to the extracellular hook and filament. We observe passive rotational diffusion of six individual *Escherichia coli* flagella lacking torque-generating units via polarization microscopy of single gold nanorods attached to the hook, sampled at 250 kHz. Transitions across energy barriers of the 26-fold symmetric LP-ring/rod flagellar bearing exhibit highly non-Poissonian kinetics spanning four orders of magnitude in time scale. At sub-millisecond timescales we observe anomalous ultra-slow diffusion typically associated with disordered systems, despite the ordered crystalline atomic structure of the bearing revealed by cryo-Electron Microscopy. Over longer periods, we observe dynamic shifts in the preferred angular positions, indicating that the bearing’s energy landscape evolves over time.

## Abstract (web summary)

Ultra-fast recording of individual rotating bacterial flagellar bearings reveals that each bearing is distinct, and variable on timescales from microseconds to minutes. This dynamic picture complements the static average structures provided by Cryo-EM.

## Introduction

Recent advances in cryo-electron microscopy (cryoEM) are revealing atomic structures of ever larger and more complicated molecular machines, leading to ever more detailed models of how they work^1–13^. Among those, the bacterial flagellum^1,2^ contains one of the best-studied of all large molecular machines: an ion-driven rotary motor ~50 nm in diameter embedded in the bacterial envelope, containing hundreds of proteins of tens of different types, called the Bacterial Flagellar Motor (BFM)^2,9,14^. CryoEM studies shed light on several parts of the flagellum^3,5,6,10,15–18^, including the bearing that anchors the flagellum in the cell wall. The bearing structure shows the 26-fold LP-ring tightly surrounding the flagellar rod, a polymer built on a helical lattice with 5-, 6-, and 11-start helices^19^. But CryoEM structures of protein complexes show averaged ensembles of frozen, isolated complexes—leaving in situ dynamics to be guessed qualitatively or occasionally inferred from differences between alternative structures^20^.

Twenty years ago, we observed the 26-fold symmetry by detecting steps in single-molecule traces of active BFM rotation^4^. At the time, we attributed these steps to the ion-powered driving process, a hypothesis later contradicted by structural data: the 26-fold symmetry corresponds to the fixed bearing^5^, whereas the stator units exhibit 5:2 symmetry^6,12^, and the cytoplasmic rotor 34-fold symmetry^10,21^. Despite these structures, nothing is known about the rotary dynamics of this, or indeed any, molecular bearing. Recent breakthroughs in protein design that allow de novo engineering of synthetic molecular bearings^22^ add to the relevance of measuring and understanding their rotation.

Here we describe the use of polarization microscopy of single gold nanorods to observe rotation with ~0.5° resolution over timescales ranging from 10 μs to tens of minutes, fully disentangling bearing dynamics from the driving process. We calculate the orientation of gold nanorods attached to the flagellar hook from the polarization anisotropy of laser illumination backscattered by nanorod surface plasmon resonance^23–25^, sampled at 250 kHz in a custom-built darkfield microscope. We resolve the passive rotation of six individual bearings: two in cells expressing sodium-powered stator units, first actively rotating and then rendered passive by removing sodium; and four with torque-generating stator units genetically deleted. Rather than free diffusion, we observe rotation dominated by transitions, separated by ~0.1 s on average (at 21 °C), between energy minima typically separated by 1/26−1/28 rev. The transition time distributions are very far from exponential, ranging from a few hundred microseconds to a few seconds. Between transitions, we observe anomalous diffusion. Furthermore, transition rates vary >10-fold between different locations in the same bearing, and even over time at the same location in a single bearing. We compute using Rosetta^26^ the bearing potential (as a function of rotation angle) predicted by the cryo-EM structure^27^, which indicates a rough landscape with heterogeneous 26-fold barriers. The unexpected dynamic behaviour of the passively rotating flagellar bearing adds an extra level to our understanding of this canonical large molecular machine, complementary to the static atomic precision of the averaged cryo-EM structure.

## Results

### High resolution measurement of flagellar rotation using polarization anisotropy of gold nanorods

**Fig. 1:**
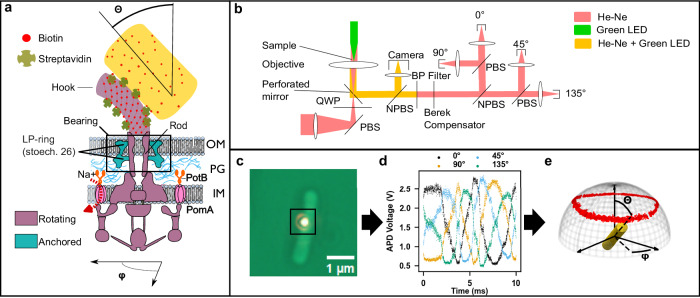
Measurement of flagellar rotation by nanorod polarization. **a** Schematic of the structure of the bacterial flagellar motor and attachment of the gold nanorod. The internal part of the BFM has been magnified 2-fold compared to the hook and the gold rod. **b** Schematic of the optical setup. Laser illumination at 633 nm (HeNe) backscattered by a gold nanorod is split into 4 polarization channels, each imaged onto an avalanche photodiode (APD). QWP: quarter wave plate. BP: 633 nm bandpass filter. PBS: polarizing beam splitter. NPBS: non-polarizing beam splitter. **c** True-colour image of a nanorod (red) attached to a hook on an *E. coli* cell (green). Thousands of such rod+cell combinations were observed. **d** Raw polarization signals obtained from a rotating motor. **e** Projection onto the unit upper hemisphere of raw nanorod orientations from the polarization signals shown in d and definition of the azimuthal angle *φ* and of the elevation angle *θ*.

To perform fast measurements of flagellar rotational diffusion, we took inspiration from experiments in which the dipolar polarization pattern of an asymmetric emitter encodes its orientation^24,28–32^ in 4 polarization channels. This compact encoding allows faster acquisition rates over longer durations than data-intensive video methods, and, unlike single fluorescent molecules, nanorods do not photobleach, which allows individual recordings of unlimited length. We attached gold nanorods to the biotinylated flagellar hook^33^ (Fig. 1a) and custom-built a laser backscattering dark-field microscope to measure their orientation (Fig. 1b). Circularly polarized collimated laser illumination reaches the sample through a hole in a mirror, which also lets light reflected by the coverslip escape back towards the laser. Light back-scattered by the rod (Fig. 1c) is reflected to a combination of polarizing and non-polarizing beam-splitters, which separate the signal into four polarizations (0°, 45°, 90°, 135° from vertical; Fig. 1d) and direct them onto four avalanche photo diodes (APDs).

Figure 1e and Supplementary Movie 1 show the orientations of a nanorod attached to a bacterial flagellum, calculated from the polarization signals using analytical formulae of Fourkas^34^ for a dipole emitter. The data are close to the ideal case of a dipole emitter rigidly attached to a flagellum rotating purely around a single axis, which would appear in Fig. 1e as a circle in a plane perpendicular to the rotation axis. The observed small deviations from this ideal may be due to the hook conformation, shifts in the rotation axis, imperfect polarization properties of the optics, possible scattering or circular dichroism induced by the cytoplasm of the cell, and deviations from pure dipole emission by the nanorod. Experimental details and assessments of potential experimental imperfections are given in Methods, Supplementary Methods S1–3, Supplementary Notes S1–6 and Supplementary Figs. S1–S7.

### In the absence of motor torque, the flagellar bearing diffuses across a finite number of predominant energy barriers

**Fig. 2:**
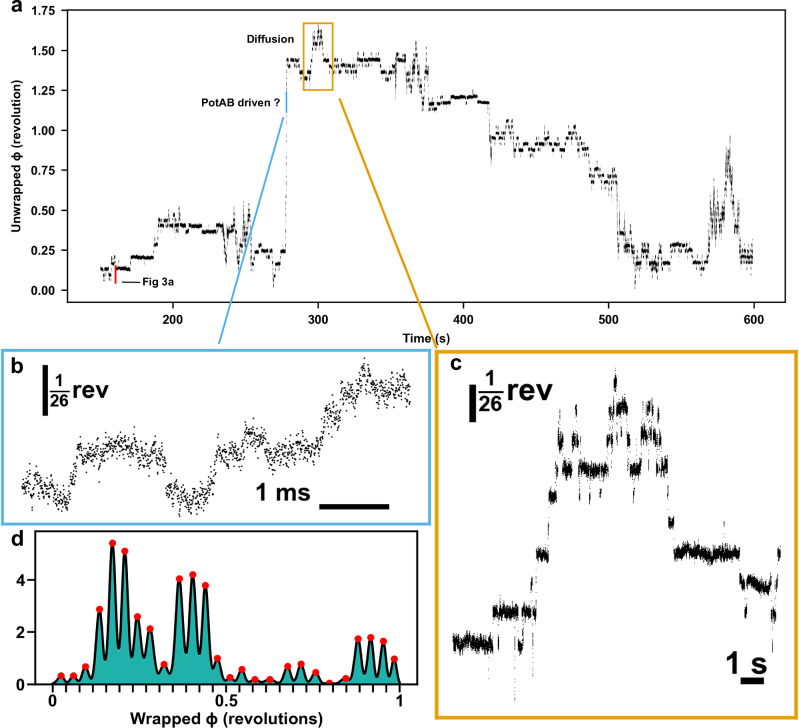
Passive rotation of the flagellar bearing. **a** Unwrapped angle φ versus time of a single motor containing Na^+^-driven PotAB stators, in ~0 mM Na^+^. A 100-points moving average is applied to the data for visibility. **b** Magnification of the blue rectangle, no averaging. **c** Magnification of the amber rectangle. The motor displays discrete jumps between stable states separated by ~1/26 rev. A 100-points moving average is applied to the data. **d** Angular distribution (histogram and Gaussian kernel density estimation) of the whole trace shown in (**a**), 26 local peaks (red) are used to define states. Tick spacing: 1/26th of a revolution.

Figure 2a shows, versus time, the azimuthal angle *φ* of a nanorod attached to a flagellum in a cell with the native MotAB stator units deleted and expressing chimeric sodium units, PotAB^35^. We selected only nanorods like this one, with a rotational axis parallel to the optical axis, ensuring that the polar angle *θ* remains nearly constant so that *φ* accurately tracks the motor’s angular position. After replacing the buffer (40 mM NaCl, 40 mM KCl) with a sodium-free solution (~0 mM NaCl, 80 mM KCl) at *t* = 0, the motor rapidly slows down from ~600 Hz to a few Hz. A few minutes later, it transitions into diffusional motion (see Fig. S8), interrupted by a single unidirectional revolution at *t* = 290 s (Fig. 2a, b), likely caused by the transient reactivation of a single stator unit energized by residual trace sodium. Closer inspection reveals that both diffusion and unidirectional rotation consist of jumps between 26 discrete metastable states (Fig. 2b–d). We confirmed that this behaviour was independent of the stator units by repeating this experiment on four motors of cells lacking them entirely (ΔMotAB), observing similar results in all cases. Our analysis was based on 960 million position samples collected over 64 min from six different bearings, representing two distinct *E. coli* strains: one expressing the sodium-dependent PotAB and the other lacking any torque-generating stator units. Supplementary Movie 1 provides an overview of an experiment on another PotAB motor, capturing the transition from ~100 Hz rotation (in 5 mM NaCl) to passive diffusive rotation of the bearing.

### Transition times between the main stable states stretch over five orders of magnitude

**Fig. 3:**
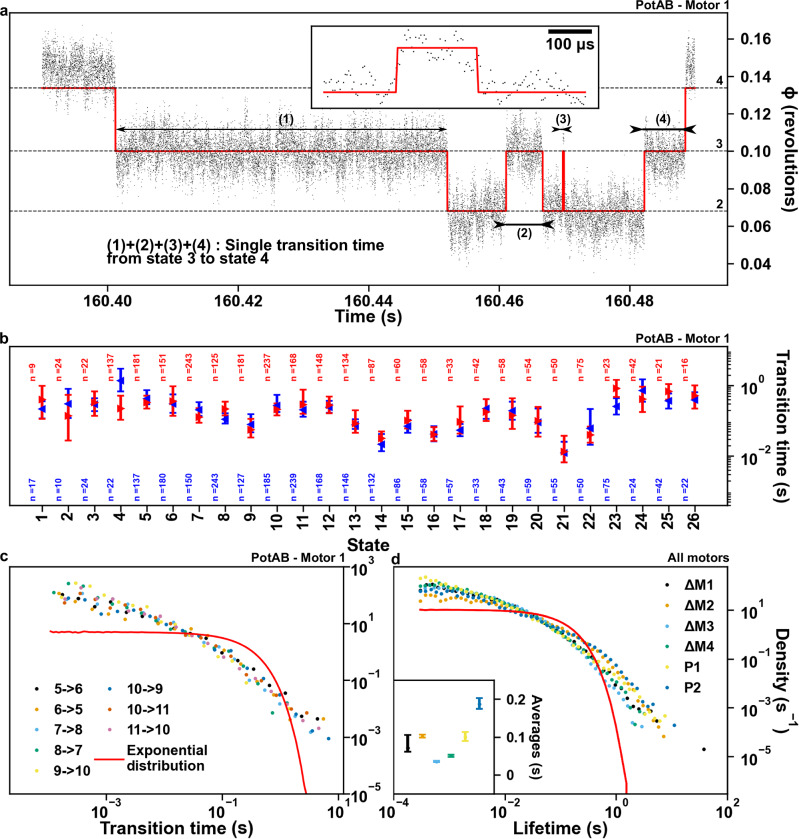
Transitions times between the main bearing states. **a** Magnified view of the red rectangle in Fig. 2a superimposed with the result of step fitting using the states defined by peaks in the histogram of Fig. 2d as prior for the state position. Dwell times of order ~0.05 s (main figure) and ~100 μs (inset, event (3) in main figure) are all clearly resolved. See “Methods” (“Transition times and lifetimes”) for explanation of (1), (2), (3), (4), **b** Average transition times out of each state, i, (blue: state i to state i − 1, red: i to i + 1). Error bars on the mean are 95% confidence intervals obtained by bootstrapping 100 times each transition time distribution. “*n*” indicates the number of samples of each transition. **c** Time distributions of the eight best sampled transitions from states labelled in Fig. 2d to a neighboring state. Red: exponential distribution with the same mean as the distribution of times from state 10 to 11. **d** Distributions of all lifetimes of all steps for all bearings, either with no stator proteins (ΔMotAB) or with chimeric Na^+^ stators (PotAB) but sodium removed. Here, all transitions are pooled. Inset: average lifetimes for each bearing. Error bars on the mean are 95% confidence intervals obtained by bootstrapping 100 times, each time distribution. Labels are abbreviations of the same labels in Fig. 4a.

All six bearings that we analyzed displayed angular accumulation points (Fig. S9–11 and Supplementary Movie S2–S7), separated by ^*1*^*/*_*N*_ rev with *N* = 27.1 ± 1.2. In some bearings a secondary separation *N* = 58 ± 9 was also seen (Fig. S12). Among those six bearings, the example in Fig. 2 (labelled “PotAB Motor 1”), had exceptionally stable state positions across the entire 470 s record, allowing us to define transitions between fixed global states consistently. Figure 3a shows the section of data marked by a red box in Fig. 2a, superimposed with the identification of the states as defined in the histogram of Fig. 2d. Figure 3b shows the average and bootstrapped errors of all transition times to or from each state (see “Methods”). For a homogeneous Poisson process (no memory), the average transition time is the reciprocal of the constant transition rate, and transition times are exponentially distributed (red line in Fig. 3c). By contrast, here, each of the 8 best-sampled transitions showed a stretched time distribution ranging from 200 μs to several seconds. The average over all transition times for this bearing was 0.20 ± 0.02 s. The fastest average to or from any given state was 0.01 s, and the slowest 1.4 s, a variation which most likely represents variable barrier heights. For bearings where global states were not consistently defined throughout the recording, a local step-finding algorithm was applied with a minimum cut-off at ^1^/_40_ rev to focus on the dominant ~^1^/_26_ rev transitions while ignoring secondary transitions. Pooled step data were used to measure individual lifetimes, defined as the time elapsed between successive steps, regardless of step destination. The distribution of these pooled lifetimes is shown in Fig. 3d and again deviates from an exponential distribution. The average lifetime was 0.08 ± 0.004 s with values ranging from 0.04 ± 0.002 s for the fastest bearing to 0.19 ± 0.017 s for the slowest (errors by bootstrapping the list of lifetimes).

If the probabilities of moving to state (*i* + 1) and (*i* − 1) were equal, the average lifetime would be half the average transition time. This relationship was confirmed for PotAB Motor 1, where the average lifetime was 0.10 ± 0.01 s. For the five bearings where global states could not be consistently defined, transition times were thus estimated as twice the measured lifetimes, yielding values of 0.08 ± 0.004 s for the fastest bearing and 0.38 ± 0.034 s for the slowest. Under the simplification that the gold nanorod, hook and bearing form a rigid body with viscous drag coefficient dominated by the gold nanorod, we used Kramers’ formula to estimate an effective barrier height Δ*U* from the transition times and independent estimates of the gold nanorod’s drag coefficient (Supplementary Note S7). A conservative range for Δ*U*, 11–16 k_B_T, is obtained by calculating the lower bound of Δ*U* using the upper limit for drag and the fastest transition time, and vice-versa for the upper bound of Δ*U*. If we assume instead that the variations in transition time are due to variations in drag, the bounds for Δ*U* are reduced to 12.5–14 k_B_T.

### Anomalous diffusion on multiple timescales

**Fig. 4:**
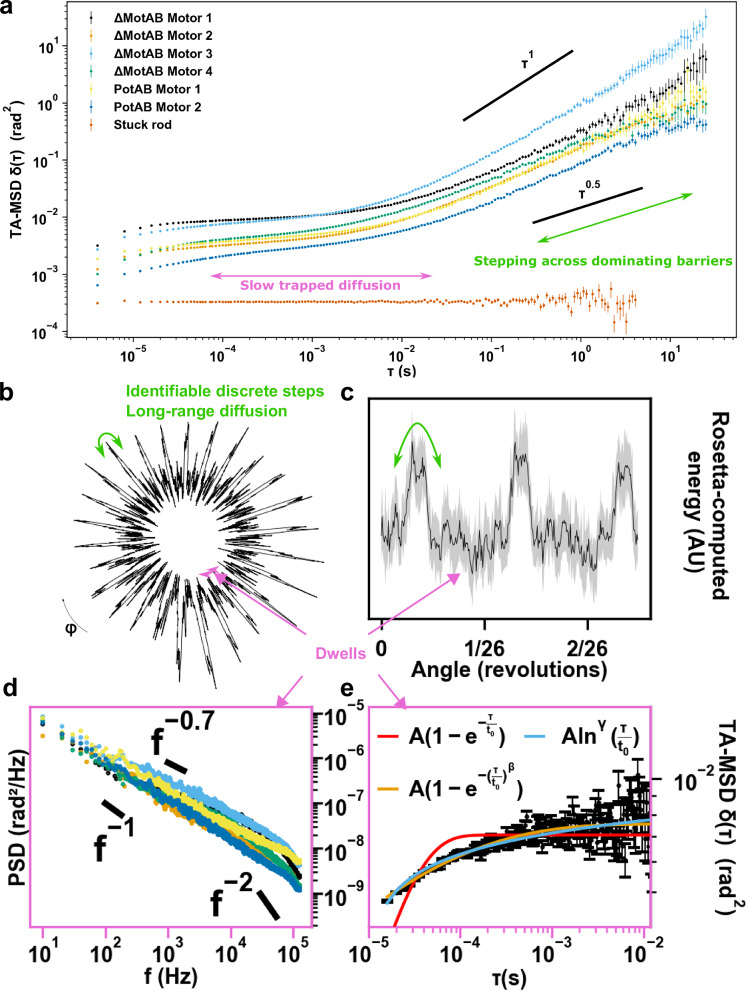
Anomalous diffusion of the flagellar bearing. **a** Time-averaged mean square displacement (MSD) of the angle *ϕ* as a function of elapsed time *τ*, for six passively rotating bearings recorded for more than 400 s each, along with a gold nanorod fixed to the glass surface. At short times, the motion exhibits slow anomalous trapped diffusion, while at longer times, faster diffusion is observed. Each point represents the average of all non-overlapping MSD values computed over full trajectories, with error bars indicating standard errors of the mean. *n* > 100,000,000 data points for each bearing. **b** Energy landscape of the LP-ring / rod interface calculated via Rosetta using the cryo-EM structure from Salmonella (PDB 7CGO, (Tan et al. 2021), see “Methods” for details) **c** Zoom on the Rosetta-computed energy landscape *vs* relative angle between the LP-ring and the rod interface. Mean (black) ± S.D. (grey) over 15 independent trajectories. **d** Power spectral density (PSD) of the angular fluctuations for six individual 1 s dwells from six different bearings. PSD are computed using Welch’s method (0.1 s Hann windows) and are smoothed at high frequencies by averaging over neighbouring points. Scaling regimes reveal approximate power-law behaviours with exponents close to −0.7, −1, and −2. **e** Time-av**e**raged mean-square displacement (TA-MSD) of an individual 1 s dwell (no steps) from **Δ**MotAB – Motor3, fitted with exponential, stretched-exponential, and logarithmic relaxation. Error bars correspond to ${{{\rm{\sigma }}}}/\sqrt{{N}_{s}}$, where s is the standard deviation of the ${N}_{s}$ independent MSD values for each lag. 250,000 > *N*_*s*_ > 2500 for all data points.

Figure 4a shows the time-averaged Mean Squared Displacement (MSD) of the angle *ϕ* as a function of elapsed time *τ* for six passively rotating bearings, each recorded for over 400 s. Additionally, data from a gold nanorod fixed to the glass surface are included as a control. At short timescales the MSD increases slowly with *τ* and cannot be fitted by an exponential relaxation (Fig. S13a–f). These features indicate anomalous diffusion - in contrast, a standard overdamped Langevin equation in a smooth periodic potential with well-defined curvature would exhibit an initial exponential relaxation followed by a linear increase in MSD with a slope of 1 at longer times.

To better understand our results, we simulated the interaction between the rod and the bearing using Rosetta^26^, based on the cryo-EM structure from Salmonella enterica (PDB 7CGO; see “Methods” for details), which has a flagellum very similar to *E. coli*. The predicted energy landscape, shown in Fig. 4b, c, has 26 non-identical, highly anharmonic low-energy states separated by barriers of varying heights. The varying barrier heights are consistent with the varying transition times (Fig. 3b), and the anharmonic potentials in each state are one possible contributor to the observed sub-diffusive behaviour at short times.

Figure 4d, e show further statistical analysis of the angular movement within six individual dwells—pauses in the angular signal exceeding 0.1 s—selected from six different bearings. While there is significant variability in the signals across different bearings, all cases exhibit sub diffusive behaviour characterized by non-Lorentzian power spectral densities (PSD) varying as *τ*^*α*^ with −1 <*α* < −0.7 (Fig. 4d), except above the instrumental APD cut-off (~125 kHz). The MSDs (Figs. 4e and S13a–f) fit very badly to an exponential relaxation model but reasonably well to stretched exponential or logarithmic models, and the process remains Gaussian across all timescales (Fig. S13g, h).

### Transition rates and dwell positions vary with time

**Fig. 5:**
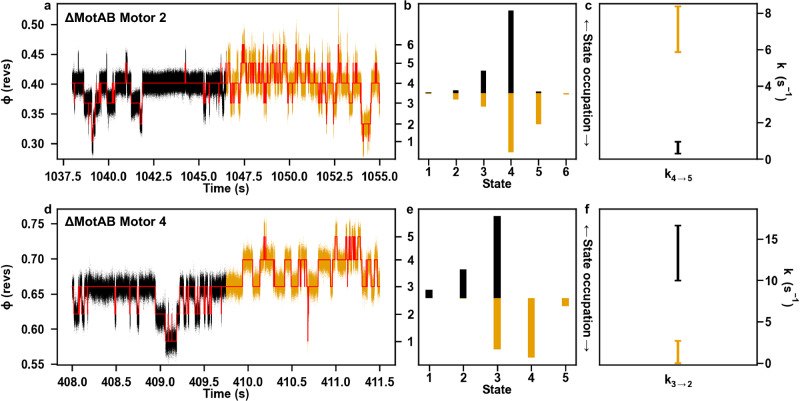
Heterogeneity of the diffusion dynamics. **a** Angle *vs* time for a passively rotating bearing showing an abrupt change in the transition dynamics, marked by different colors. Black, amber: raw data. Red: step-filtered. **b** State occupation (proportion of points from (**a**) belonging to each state) for each of the six discrete states observed in *a*. for each part of the time trace, as indicated by matching colors. The occupation of amber states is displayed as positive downwards for better visibility. **c** Transition rates from state 4 to state 5 for each half of *a*. ${k}_{4\to 5}={n}_{4\to 5}/{T}_{4}$ where ${n}_{4\to 5}$ is the number of detected steps for state 4 to 5 and ${T}_{4}$ the total time spent in state 4. Errors are computed as $\sqrt{{n}_{4\to 5}}/{T}_{4}$. *n*_*4→5*_ = 4 (black), 32 (orange). **d**–**f**. Same as a,b,c with another example from another bearing. *n*_*3→2*_ = 16 (black), 1 (orange).

**Fig. 6:**
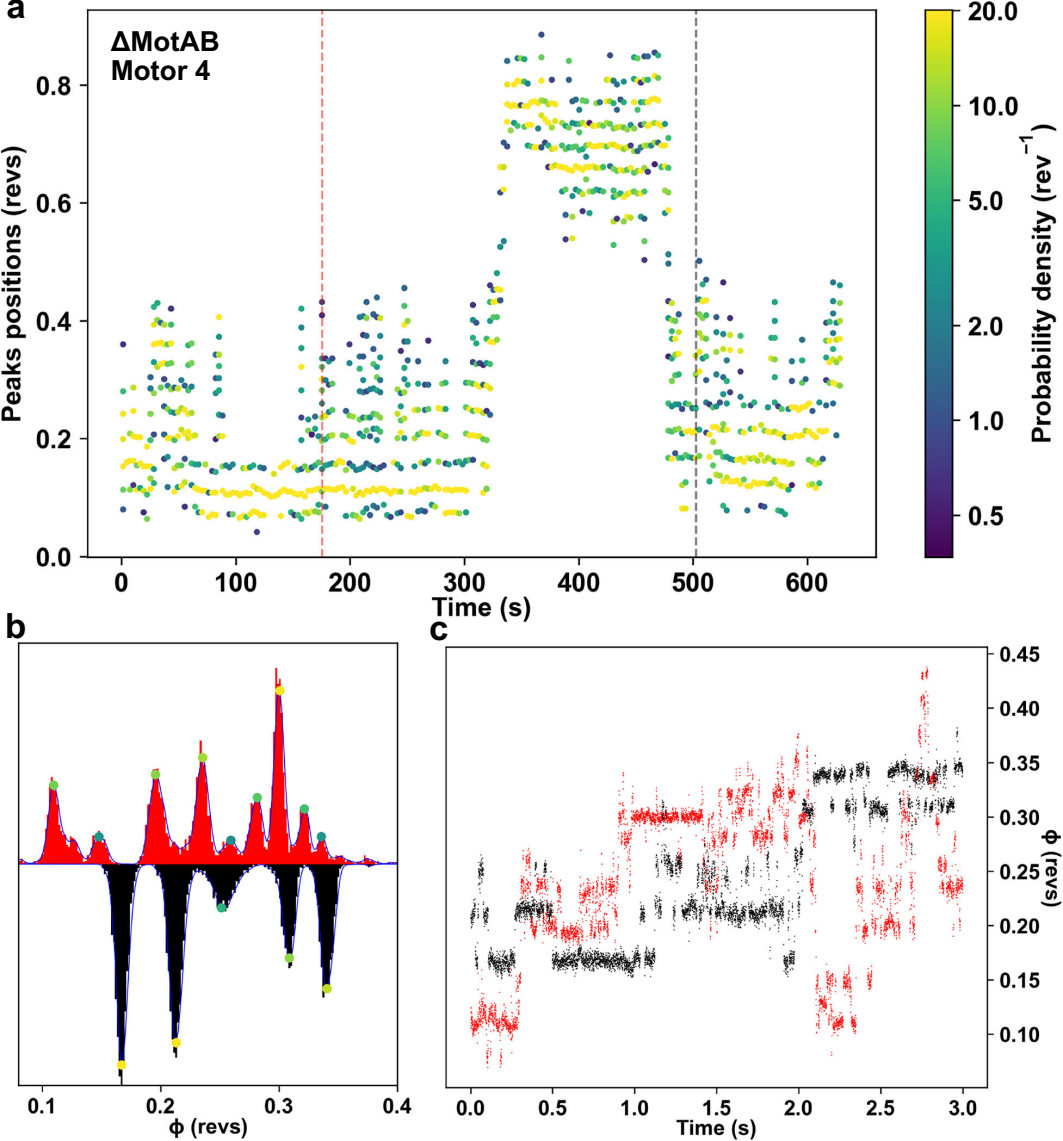
Variation of dwell angles over time. **a** Evolution of the position of peaks in angle histograms of successive non-overlapping 3 s windows for ΔMotAB Motor 4. Color bar: probability density (rev^−1^) at each peak. Low peaks (dark blue) are more prone to errors. **b** Angular distributions (histogram and Gaussian kernel density estimation) and peaks for 2 windows starting at times indicated by vertical lines in matching colours in (**g**). **c**. Angle vs time trace of the windows indicated in matching colours in (**a**, **b**). A 100-points moving average has been applied to the data.

To get better insight into the stretched distributions of transition times, we looked for dynamical heterogeneities in our records. We found two different types, illustrated in Figs. 5 and 6: both the rates of transitions between the main states and the locations of the states changed on timescales of several seconds to minutes. Figure 5a shows raw angle versus time for a 20 s portion of data from one bearing, overlaid with step fits as in Fig. 3a to the 6 main states identified in this portion. The dynamics of the system changed at 1046 s, marked in the figure by the colour change. The second most occupied state changed from 3 to 5 (Fig. 5b), and the transition rate 4 → 5 increased more than 5-fold (Fig. 5c). This rate change is statistically significant at a level *P* ~ 0.0000000005: if the entire trace represented a single Poisson process, the probability of no more than the observed 6 transitions in the black part of the curve, given the lower bound of the estimated rate in the orange part of the trace (*k* = 7.0 s^−1^), would be 1.3 × 10^−12^. Figure 5d–f show another similar example from another bearing.

Figure 6 shows an example of the second type of change. Figure 6a shows the positions of peaks in the dwell-time distributions over successive 3 second intervals for the bearing of Fig. 5d–f. Figure 6b shows the histograms and Fig. 6c the angular trace of the bearing for two intervals separated by more than five minutes and identified by black and red dashed lines in Fig. 6a. Both intervals sample the same angular range (0.1–0.4 rev), but the later interval (black) shows fewer and different states, showing that the local energy landscape has changed. Similar shifts in rates and states were found in all bearings observed (Supplementary Movies S2–7).

Supplementary Note S8 describes a simple lattice simulation based on the arrangement of proteins in the rod. It predicts the observed bearing variability as arising from variation in the defects represented by non-identical LP-ring repeating units and/or by slight shifts in the alignment of rod and LP-ring.

## Discussion

We measured the passive rotation of the bearings of six individual *E. coli* bacterial flagella with ~10 µs resolution over tens of minutes. As in active rotation, we saw 26–28 dominant dwell angles, consistent with the known symmetry of the LP-ring. Our gold nanorod polarization setup, which extends the dynamic range by several orders of magnitude compared to previous methods, enabled us to record thousands of transitions between these dwell states per bearing. Each bearing shows anomalous diffusion and structural dynamics over 4–5 orders of magnitude in timescale, characteristic of a system with a very large number of metastable configurations. While we cannot exclude the possibility that external factors such as hook dynamics or interactions between intracellular and cytoplasmic flagellar components may contribute to the observed dynamics, the observed ~26-fold symmetry is strong evidence that the bearing is the dominant contributor.

We estimated that the average energy barrier height between states Δ*U* is likely to be between 12.5 and 14 k_B_T. The corresponding equivalent torque *ΔU/δϕ* = ~200 pN nm (where *δϕ* is the angle between adjacent energy minima) is several times smaller than the torque generated by the flagellar motor, and therefore the bearing is smooth enough not to impede motor rotation. 200 pN nm is of similar magnitude to the estimated stall torque per torque-generating stator unit^7^, inviting the speculation that stator unit torque and the energy barrier of the bearing may have coevolved to ensure that one unit can overcome the bearing’s barriers.

We found that transition times associated with the main barriers span several orders of magnitude, deviating greatly from Poisson statistics (Fig. 3b–d). This deviation persisted even when analysing transitions over individual barriers, indicating that the pooling of transitions across heterogeneous barriers cannot be its sole cause. We hypothesize that this stretching may arise from the temporal pooling of transition times—specifically, from transitions that are locally exponentially distributed but occur at rates that evolve over time. Figure 3b illustrates that transition rates out of a particular state are better correlated than those across a given barrier, in this particular bearing. For example, states 14 and 21 appear to be particularly stable, with low outgoing transition rates in both directions. We have no explanation for this observation that particularly stable states appear to be more likely than particularly high or low transition barriers. Examining the data in greater detail at longer timescales (>1 s), we observed shifts in both kinetics and angular occupation within single bearings (Figs. 5 and 6). This reveals a form of structural plasticity that remained undetectable using standard approaches.

The stretched transition distributions could also be due to random trapping in long-tail distributed traps. This hypothesis is supported by the fast dynamics of the bearing within individual dwells located between the main barriers, which displays no characteristic time (Fig. 4d, e). At those scales, the mean square displacement (MSD) can be approximated by a stretched exponential or logarithmic dependence on time lag *τ*, consistent with systems exhibiting long-tailed energy landscapes^36^. Molecular processes characterized by multiple timescales have been observed in a wide range of cases; examples include the diffusion of particles in crowded and heterogeneous environments such as the cytoplasm^37–39^, protein folding in response to transient perturbations^40–42^, spectroscopic studies of populations of globular proteins^43^, spatial fluctuations in single complexes^44^, DNA hairpins^45^—but never before in a single, well-folded large molecular machine. To relate previous findings to the flagellar bearing, we note that our measured rotation angle is a 1D projection of the multidimensional landscape of a very large protein complex. Any angular path likely corresponds to many possible paths in the whole coordinate space and thus to a variety of transition times, all dependent on fast rearrangements of small protein domains—particularly close to the tight bearing interface^5^.

Our Rosetta simulations (Fig. 4b, c) demonstrate that sensitivity to small features at the atomically-tight sliding interface of the flagellar bearing results in a rough interaction potential, that may in part explain the observed heterogeneities and anomalous diffusion. Current Rosetta based energy scoring algorithms cannot efficiently account for combinatorial explosion of the conformational space, which prohibits modelling of either static or dynamic heterogeneity. Instead, Supplementary Note S8 describes a simple simulation based on the bearing symmetries which predicts the observed dwell angle periodicities in actively rotating BFMs (Fig. S14) as consequences of rough features in the bearing interface and non-identical LP-ring repeating units. Bearing heterogeneities arise by simulating variation in the defects represented by non-identical LP-ring repeating units and/or by slight shifts in the alignment of rod and LP-ring. For example, the observed shifting and splitting of dwell angles over time in a single bearing (Fig. 6) might be explained by changing conformations of individual LP-ring or rod monomers.

Overall, these findings suggest that even well-structured, simple, symmetric molecular machines like the flagellar bearing exhibit rich internal dynamics, persisting even at long timescales. Given the large number of interacting proteins (>75), such behaviour may not be unexpected and may be a characteristic of all large protein complexes. Whether these dynamic energy barriers serve a functional role in the BFM—and rotary motors more generally—by enabling better adaptation to varying torque, proton motive force, and ionic conditions, or whether they persist simply due to weak evolutionary pressure against them, remains an open question.

## Methods

### Attachment of gold nanorods to the hook of the bacterial flagellar motor

100 µL of overnight cultures of bacterial strains bearing AviTag mutations on FlgE^33^ are inoculated in 10 mL Tryptone Broth (10 g NaCl, 5 g Difco Tryptone) + 100 nM D-Biotin and grown at 30 °C until they reach OD 0.4. 200 µL is then harvested and rinsed three times by successive centrifugations (2.5 min at 5000 × *g*) with 200 µL motility buffer (40 mM NaCl, 40 mM KCl, 10 mM K_2_HPO_4_/KH_2_PO_4_, pH 7.1). 5 µL of neutravidin solution (90 mM in water) is added to the cells and incubated 20 min with agitation at room temperature. The cells are then rinsed 4 times with 200 µL motility buffer to remove excess neutravidin. They are centrifugated a 5th time and resuspended in 15 µL of motility buffer + 5 mg/mL BSA. 0.5 µL of biotinylated gold nanorods (40 × 40 × 68 nm, Nanopartz C12-40-600-TB-DIH-50−1) is quickly added, and the mixture agitated for 1 min before transfer to centrifugal filtering columns with 0.22 μm pores (Millipore UFC30GV0S), and 150 µL of motility buffer + BSA are then added. To remove unattached gold, filtering columns are centrifugated 35 s at 5000 × *g* (just enough to pass the solution but keeping the membrane wet). 200 µL of motility buffer + BSA are added again, and the filtering procedure repeated four times. For the last repeat, we do not centrifugate but instead vortex the column for one minute to detach the bacteria from the membrane. The solution above the filter is then retrieved, transferred to an Eppendorf tube, and centrifugated 2:30 min at 5000 × *g*, a smaller pellet with a slightly darker color indicates of gold attachment. The pellet is resuspended in 50 µL motility buffer without BSA for later use.

### Microscopy setup

A Helium-Neon laser (12 mW, 633 nm, Melles Griot 25-LHP-991-230) is focused onto the back focal plane of an oil-immersion objective (100X Nikon Plan Fluorite Oil Immersion Objective, 1.3 NA, DIC) using a biconvex lens. A telescope made of two plano-convex lenses and two dielectric mirrors is added upstream to precisely adjust the focusing point of the laser. A linear polarizer and a *λ*/4 plate are added to circularize the excitation light. We ensure darkfield by drilling a hole (~1 mm diameter) in a dielectric mirror at 45° to the optical axis, between the objective and the *λ*/4 plate, so that light reflected by the objective, coverglass, and slide returns through the hole into the excitation path while light scattered by gold nanorods is mostly reflected into the emission path. The emission path consists of beam-splitters which separate the signal into four polarizations: 0°, 90°, 45°, and 135°. A first non-polarizing beam-splitter (Edmund Optics #35-963) reflects half of the light towards the camera. A second identical non-polarizing beam-splitter separates the light between two paths. On the first path, a polarizing beam splitter with vertical axis separates the 0° and 90° polarizations. On the second path, a polarizing beam splitter with an axis at 45° separates the 45° and 135° polarizations. A linear polarizer is added on each exit face of the polarizing beam splitters to remove leaks from s- and p- polarizations from the p- and s- faces, respectively. Importantly, we use a phase compensator (in our case a Berek’s variable waveplate) to retrieve 45° and 135° polarizations: with the emission path in a horizonal plane, each dielectric mirror adds an uncharacterized phase shift between its s- and p- polarizations (0° and 90°). This does not affect the intensity of the 0° and 90° polarizations but mixes the 45° and 135° together. The Berek compensator (Newport 5540M) reverses the accumulated phase shift, un-mixing the 45° and 135° polarizations. The light in each polarization channel is then focused onto the capture area of one of four different avalanche photodiodes modules (Hamamatsu C5460-01). Supplementary Method S1 shows further details of our system.

### Cell strains

Strains MTB16 and MTB24 are derived from YS34^4^, itself deriving from RP4939 (ΔCheY), with the following modification: ΔMotAB, ΔFimA, and ΔFliC. MTB24 expresses the plasmid pYS13^46^, which encodes PotAB under IPTG induction.

The hook protein FlgE of MTB16 and MTB24 contains an AviTag sequence (GLNDIFEAQKIEWHE) inserted between residues I221 and A222, “site C” as described by Brown et al.^33^. The AviTag is endogenously biotinylated by BirA.

### Excitation path

We position the drilled mirror on a translation stage, the hole in the mirror being roughly aligned with the vertical optical axis. We then mount the objective on a threaded plate 2 cm above the mirror: it remains fixed throughout the experiment, focusing is performed by moving the sample vertically. The *λ*/4 plate and the linear polarizer (or a polarizing beamsplitter) are placed below the drilled mirror. The focusing plano-convex lens is placed at its focal length from the back of the objective. A coverslip containing a dried solution of gold nanorods and micrometric beads is imaged on a camera using a lens of short focal length (5 cm) focused to infinity, and the sample moved vertically until beads appear sharp on the camera. Then the three following steps are repeated until convergence: (1) The laser beam is walked using two mirrors until it emerges vertically from the objective, AND the area excited by the laser appears in the center of the field of view on the camera. (2) The lateral position of the drilled mirror is adjusted to maximize the intensity of the excited area and minimize scattered light at the mirror. (3) The position of the second lens of the telescope is adjusted to collimate the laser beam emerging from the objective. 1) and 3) are judged by observing the location and size of the laser spot on the ceiling.

### Emission path

All polarizing and non-polarizing cubes are positioned at the same height as the corresponding APD’s and carefully rotated—using the reflection of an alignment laser—so that their axis is perpendicular to direction of propagation of the light. A field of view containing a single excited nanorod is then used for fine tuning and the two following steps are repeated until convergence, to ensure that the gold rod is centered in the field of view and its image in the APD detector: 1. the orientation of the second non-polarizing cube is slightly tuned to maximize the signal in the first APD 2. the position of the nano-rod in the field of view is tuned using the nano-positioners to maximize the signal in the first APD. Then, the mirrors reflecting light to the three other APD are fine-tuned to maximize the signal in each detector. Note that to be able to correct their phase-shifts with the phase compensator, all dichroic mirrors should be placed in parts of the optical path where light scattered by the nanorod is collimated, since the induced phase shift is very sensitive to the angle of incidence. The Berek compensator is aligned the following way. We first set its retardance to 0 and adjust its orientation so that the reflected beam propagates back along the incident beam. We then adjust the orientation of the plate so that when changing the retardance, the 0° and 90° APD signals remain unchanged. Finally, we used rotationally diffusing nanorods non-specifically attached to the glass to adjust the retardance of the plate to maximize the amplitude of variation of the 45° and 135° channels during rotation.

### Data acquisition

We recorded the voltage of each APD module (Hamamatsu C5460-01) at 250 kHz in differential mode using a multi-channel analog to digital converter (National Instruments PCI-6143) connected to the computer through a programmable chassis (National Instruments PXI-1071). The APD modules are connected to the converter through coaxial cables and a controller dock (National Instruments BNC-2090A). 100 kΩ resistors are soldered onto the R31, R32, R28, R27, R23, R24, R20, R19 slots of the controller dock, corresponding to the path between each analog input (+ and −) and the ground of each channel. This allows loading down the APD module output to the recommended range of 200 kΩ as well as using floating sources: we power the APDs with two 12 V batteries in series to avoid power-supply noise and earth loops in the circuit. APDs are insulated from the optical table. Black cardboard walls around them are coated with aluminum foil to shield the modules from electromagnetic noise (coming, for example, from the laser power units). The chassis is connected to the computer through a PCIe remote control interface card (51-924-001) on the computer side and PXI Remote Control Interface module (41-924-001) on the chassis side. The voltage of each APD module is recorded and streamed to file using a home-made LabVIEW script.

### Flow cell preparation

A clean coverslip was first plasma-cleaned and assembled with a glass slide into a flow cell. The chamber was then incubated 5 min with a solution of 0.1 M acetic acid and 0.015% chitosan (Sigma-Aldrich 448869) to promote bacterial attachment. The chitosan solution was thoroughly rinsed with motility buffer. After rinsing, cells with nanorods attached to hooks were introduced in motility buffer and allowed to adhere. Non-attached cells were subsequently removed by gentle rinsing before imaging. Imaging was performed at 21 °C in zero-sodium motility buffer (0 mM NaCl, 80 mM KCl) except where otherwise specified.

### Cell selection

The full 200 µm field of view is illuminated with a green LED (Thorlabs M530L3). We bring each bacterium one by one, with a manual nano-positioner, to the much smaller field of view excited by the laser (Gaussian field of half-width *σ* = 1.4 µm). The maximum intensity of the laser excitation, calculated as ${\,P}_{\max }=\frac{{P}_{{tot}}}{2{{{\rm{\pi }}}}{{{{\rm{\sigma }}}}}^{2}}$, where ${{{{\rm{P}}}}}_{{{{\rm{tot}}}}}$ is the full power measured with a power meter placed above the sample, is tuned to ~200 W/cm^2^. A bright red point-spread-function is clearly distinguishable on the camera when a gold nanorod is attached to the bacteria. We display live the anisotropy of the polarization signal ($x=\frac{{{{\rm{I}}}}0-{{{\rm{I}}}}90}{{{{\rm{I}}}}0+{{{\rm{I}}}}90}$ versus $y=\frac{{{{\rm{I}}}}45-{{{\rm{I}}}}135}{{{{\rm{I}}}}45+{{{\rm{I}}}}135}$) from the APDs in successive 0.5–1 s blocks, and the power spectral density (PSD) of $x+{{{\rm{i}}}}y$. The PSD of the combined anisotropy of a rotating rod attached to an active BFM displays a clear peak in frequency, indicating that the rotation is periodic. Displaying the anisotropies also allows us to differentiate between different orientations of the rotation axis and angle of rod attachment, and thus to scan over several rods per minute to find those with desirable orientations. Two similar overlapping circles in the space of anisotropies indicate a rod whose rotation axis is close to the optical axis—one circle corresponds to *ϕ* in [0,π], the other to *ϕ* in [π,2π]. When the rotation axis is further from the optical axis, two separate loops appear with different radii. We select the first type of trajectory (<10% of all rods) to avoid points of low total intensity (rods near vertical, *θ* ~ 0), where the scattering cross-section is smallest and much light is scattered perpendicularly to the optical axis and doesn’t enter the objective. At those points, the optical noise is thus larger and makes the analysis harder to perform, including the possibility of losing the corresponding sections of rod trajectories. By contrast, a rotation axis close to the optical axis allows us to consider the optical *ϕ* azimuthal angle of the rod to be equal to the angle describing the rotation of the motor, i.e., our variable of interest. These selection criteria reduce the throughput of the experiment in terms of cells. However, the throughput in terms of total samples and recording time is large due to the high sample rate and absence of photobleaching of the gold.

### Orientation reconstitution

We reconstitute the orientation of individual nanorods from the polarization signals using previously published analytical formulae for ideal dipolar emitters and perfect optics (Supplementary Method S2). The intensity of each of the four polarizations focused onto APDs is measured at an acquisition frequency of 250 kHz and corrected for imperfections of polarizing beamsplitters and APDs (Supplementary Note S1). Simple simulations based on ray optics suggest that the hole in the mirror does not affect much the inference of nanorod orientation, even though it removes part of the light scattered by the rod (Supplementary Note S2). There are two degeneracies affecting the orientation inference: the transformations *ϕ* → π + *ϕ* and *θ* → -θ leave the polarization signal unchanged. The first is not problematic if *θ* is far from 0 (rod orientation far from the optical axis) because we can unwrap *ϕ* by continuity. The *θ* degeneracy is not a problem either if the axis of rotation is close to the optical axis, so that most of the rotation of the rod is encoded in *ϕ*. We note that the degeneracies could be partially lifted by recording asymmetry in the scattered intensities in the back focal plane, which we have not attempted. To estimate the measurement noise due to the optics, we attach rods to the surface, infer *ϕ*, and inspect the power density function of the signal. The *ϕ* signal of such a rod over a 1 Hz–125 kHz measurement bandwidth displays a standard deviation from ~1° to ~0.4° at a working APD voltage, summed over the four APD’s, of ~1 to ~12 V, respectively (Fig. S3). Summed APD voltages for bearing measurements ranged from ~1 to ~11 V.

Estimates of the heating due to light absorption by nanorods (Supplementary Note S4) and the torque exerted by circularly polarized light (Supplementary Note S5) indicate that both are negligible. We also measured the rotational drag of ten rotating rods loosely attached to a glass surface (Supplementary Note S6).

### Molecular simulation of the interaction potential

We include the code for the Rosetta simulations at https://github.com/alexiscourbet/Bacterial-flagellum-energy-landscapes, along with the images referenced below. The structure of the bacterial flagellar motor-hook complex was retrieved from the Protein Data Bank (PDB: 7CGO, 7CGO_full1.png) and truncated to account only for the molecular interactions at the interface between the stator and rotor (7CGO_trunc*.png). The rotational energy landscape was generated by rotating rod and LP-ring components relative to each other around an axis close to the near-symmetry axis of the LP-ring, sampling every 0.1065088757396449704142011834° (rotate_rotor_stator.py) to produce 3380 new starting structures ( = 360/26/13/10, which accounts for the symmetry of the rotor and stator in the sampling and prevents aliasing artifacts), one for each rotation angle (/example_sampled_rotations/*.pdb). Rosetta energies (Total_score) after repacking all residues (repack.xml, PackRotamersMover) were then computed for the whole rod and LP ring complex, with 15 repeat trajectories for each angle. Inspection of the protein-protein interface of rotamer minimized structures between stator/rotor reveals low energy configurations and intricate hydrogen bonded interactions (7CGO_interfcaehbind1.png). The rotational energy landscape can be efficiently visualized as a polar plot showing the mean over repeats (Fig. 4b), with 26 “spikes” corresponding to high energy barriers.

### Data visualization and analysis

Large data files of tens of millions of points are smoothly visualized using a home-made program written in Python, which itself uses the visualization interface pyqtgraph (www.pyqtgraph.org).

### Power spectral densities

Power spectral densities (PSDs) are calculated using the Welch method from the scipy.signal module. A Hann window is applied with a 50% overlap.

### Identification of global states

The process begins with a smoothing operation applied to the unwrapped angles using a finite impulse response (FIR) window of size 100. Then a Gaussian kernel density estimation (KDE) of bandwidth 0.002 rev is applied to the wrapped angles. Peaks in the KDE curve are identified based on a prominence threshold of 0.5 rev^−1^ (red points in Fig. 2d), corresponding to global states that represent significant, recurring angular dwells. For most cells, we found that the global states are themselves not stable over the whole recording. They are thus calculated by applying the KDE estimation over 3 s windows. The positions of the identified peaks over the whole recording are shown as a function of time in kymographs such as in Figs. 6a and S9–11.

### Identification of transition between states

The process begins with the identification of global states as described above. Then, a preliminary set of state transitions is determined using a moving step-fit^47^ algorithm, with the threshold set to limit false positive rate to fewer than 0.0001 for long dwells. The states between detected steps are subsequently aligned to the global states by minimizing the distance between them. If the global states cannot be clearly defined for a particular motor, the global step identification is omitted, and all steps shorter than 10° are discarded (a threshold significantly, but not too far, below the 13.6° average of the dominant 1/26 rev transitions that we are aiming to quantify).

### Transition times and lifetimes

Once the time trace has been reduced to a list of transitions between discrete states, we define the transition time from A to B as the time spent in A between two A → B events, and we define the life time of A as the time spent in A between the event leading to A and the event leaving A. For example, in Fig. 3a “(1)” indicates one lifetime in state A = 3, while the sum of “(1)”,“(2)”,“(3)” and “(4)” is a transition time from A = 3 → B = 4.

### Reporting summary

Further information on research design is available in the Nature Portfolio Reporting Summary linked to this article.

## Supplementary information

Supplementary Information

Description of Additional Supplementary Files

Supp Movie 1

Supp Movie 2

Supp Movie 3

Supp movie 4

Supp movie 5

Supp movie 6

Supp movie 7

Reporting Summary

Transparent Peer Review file

## Source data

Source Data

## Data Availability

All raw and processed data generated in this study have been deposited in the Zenodo [https://zenodo.org/records/20088460]. Source data are available within the Source Data File. Source data are provided with this paper.

## Code availability

All the code used to process and interpret the data have been published in a GitHub repository linked to Zenodo10.5281/zenodo.20094669.
