# Supplementary material for: Heterogeneity and multi-scale dynamics in the molecular bearing of the bacterial flagellum
Source: Nat Commun. 2026 Jun 12;17:7496. doi: 10.1038/s41467-026-74079-9 (PMC13408457; doi:10.1038/s41467-026-74079-9)
Supplement: Supplementary file 1 — Supplementary Information [file 41467_2026_74079_MOESM1_ESM.pdf]

- **Supplementary Methods S1 to S3**
- **Supplementary Notes S1 to S9**
- **Figures S1-S19**
- **Supplementary References**

Supplementary Method S1: Experimental Setup and Alignment procedure

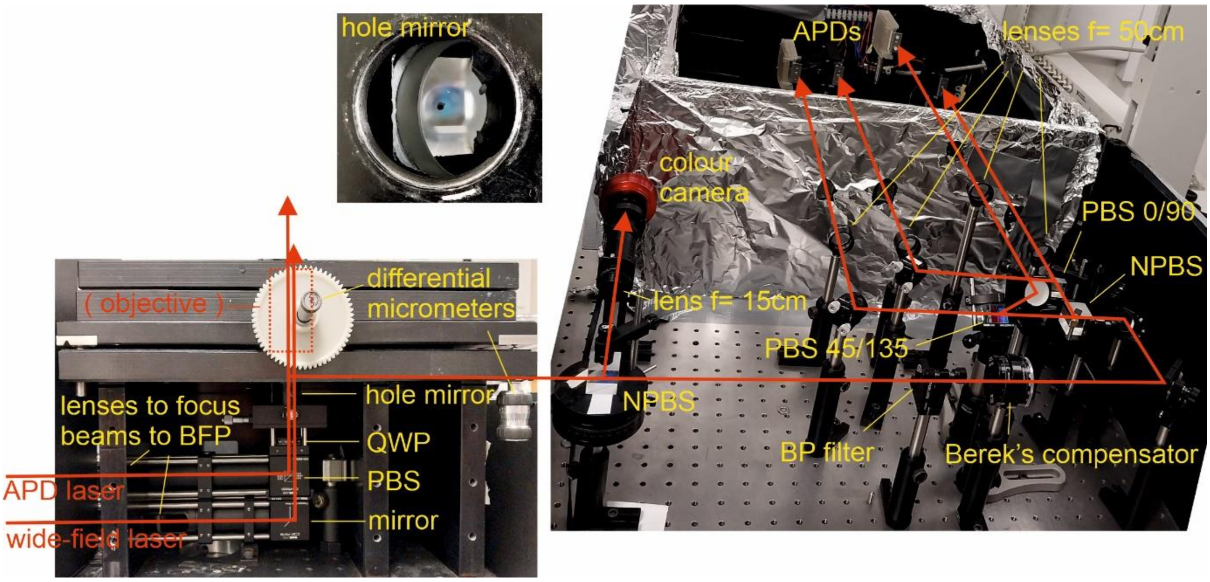

Figure S1: Optical table layout

Main stage configuration

The layout of the experimental system is shown in Fig S1.

The main stage is made of three levels.

The first, lowest level holds a micrometric translation stage supporting a dielectric mirror ("hole mirror") mounted at 45° to the optical axis, with a hole ~1 mm in diameter along the optical axis, drilled by hand using a diamond drill bit (photo shows the hole mirror viewed from above with the objective removed).

The second level holds the fixed objective just above that mirror. The 45° mirror fits into the objective hole of that level, in order to bring it as close as possible of the back focal plane (BFP) of the objective, which is inaccessible inside the objective structure for our typical 100X high NA objective.

The second level plate is also bored horizontally (~ 2 cm diameter). The space between the two first levels that is created by this cylindrical bore allows light reflected by the mirror to be transmitted horizontally into the excitation path.

The third, highest level is a stage made of 3 horizontal plates placed directly in contact with each other. This holds the sample and its vertical and horizontal position can be adjusted with micrometric screws ("differential micrometers").

An opto-mechanical cage is mounted below the first level. It contains a $\lambda/4$ plate ("QWP") and a polarizing beamsplitter ("PBS") reflecting the light from the horizontal excitation path into the objective. This PBS combines the excitation laser beam for the APDs with a wide-field beam. These

beams exit the PBS with orthogonal polarizations, ensuring that both beams are polarized at 45° to the QWP and therefore circularly polarized at the sample, ensuring isotropic excitation of nanorod scattering.

Alignment of the excitation path

A. Preliminary alignment

1. The drilled mirror is removed from the path. This allows the observation of the objective back reflection in the excitation path.
2. The objective is removed to allow a first approximate alignment of the laser. The latter is beam-walked until it goes through the centre of the hole holding the objective and reaches the ceiling of the room above the objective position.
3. The objective is placed back and will remain fixed. The laser is beam-walked with two mirrors until the back-reflection aligns with the beam.
4. The plano-convex lens (f_0 = 30 cm) is added to the excitation path at a distance f_0 +/- 0.5 cm from the objective exit pupil, using the back reflection to adjust its orientation and vertical position. It will then remain fixed.
5. A pair of lenses (f_1 = 10cm, f_2 = 20cm) are used as a telescope (separation $d = f_1+f_2$) to increase the laser beam width. A larger beam results in a larger aperture of the beam converging into the BPF of the objective and thus a larger excitation area in the field of view. Their vertical position and orientation are adjusted using their back-reflections.
6. The position of one of the three lenses, possibly held on a magnetic support or opto-mechanical stage, is adjusted until the beam exiting the objective appears at its smallest on the ceiling of the room (typically ~20 cm diameter). This focusses the laser beam into the back focal plane of the objective.
7. During steps 4-6, if all back-reflections remain aligned with the input laser beam, the laser beam should roughly exit the objective vertically. If it does slightly move laterally, adjust with the mirrors used to perform the beam walk in step 3.
8. Put the drilled mirror back in place. At first its body will appear red, because the laser beam scatters from the walls of the hole. Adjust its position until it becomes as dark as possible (some scattering may remain).
9. Adjust the axis of the λ/4 plate until the light exiting the objective is circularly polarized, or at least homogeneously polarized (the intensity of the light should not depend on the orientation of a linear polarizer placed between the objective and the power meter).

B. Precise alignment of the drilled mirror.

The drilled mirror will now be thoroughly centred with regard to the back focal plane of the objective.

1. Precisely assemble a camera at the back focal plane of a 4f lens setup to use as an imaging tool.
2. Image the back-focal plane of the objective with that setup (where the ceiling of the room appears sharp).
3. Put a paper in contact with the pupil of the objective, add a drop of immersion oil on the paper. Illuminate the full entrance pupil of the objective with a roughly focused LED. The paper serves as diffuser to fill the whole BFP of the objective. The back focal plane should now be a rather homogeneous circle with an unfocused dark central hole.

4. Slightly adjust the axial position of the imaging 4-f setup until the hole of the mirror appears sharp (typically a few mm). The back focal plane of the objective will still appear circular but slightly defocussed.
5. Adjust the lateral position of the mirror with the micrometric screws until the hole is centred compared to the circular back focal plane.

C. Precise alignment of the excitation area

The area of the field of view which is excited by the laser should now be centred so that all polarizations and rays scattered by the excited gold nanorods are captured homogeneously.

1. Assemble a camera at the focal plane of a short focal-length lens (~ 5 cm) to be able to observe the whole field of view of the objective.
2. Place the camera as close as possible to the lateral output of the stage, to image the full uncropped entrance pupil of the objective.
3. Prepare a coverslip with a dried solution of gold nanorods. Add a second coverslip, a thin paper and a drop of water on top. Place the sample on the sample stage.
4. Simultaneously illuminate the field of view from above, for example with a green light, as in B.3, and with the laser.
5. The gold nanorods will now appear in red in the excited area while the rest of the field of view will appear green. Due to the presence of the drilled mirror and the imperfection of the diffusion of the green light, the centre of the field of view might appear darker.
6. Adapt the height of the sample stage so that the gold nanorods are focused.
7. Beam-walk the laser beam until the excitation area lies in the centre of the field of view and the reflection on the sides of the field of view are minimal.

Alignment of the emission path

First, note that no lens should be placed between the objective and the horizontal mirrors preceding the polarizing cubes. Indeed, dichroic mirrors add a phase between s- and p- polarization which will affect the measurement of 45° and 135° polarizations and is strongly DEPENDENT on the incident angle. Any beam reflected before the separation of the polarization should be collimated so that all incident angles on the reflection area are equal.

A. Preliminary alignment

1. Precisely measure the reflection and transmission coefficients of all non-polarizing and polarizing cubes of the setup, for both p- and s- polarized light. They will be used later to compute their effect on the measured signal.
2. Place a first non-polarizing cube path to send half of the light (reflected) to a bright-field camera placed in the back focal plane of a lens of focal length ~15-20 cm.
3. Take a sample with a low density of gold nanorods, and move the sample in the field of view until a single gold nanorod appears in the area excited by the laser. Focus the sample so that the nanorod appears sharp in the camera. Now, in a dark room and with the power of the laser at its highest, you can see a collimated image of the gold nanorod transmitted by the cube. It is circular (typically 5 mm of diameter with a 100X objective) with a hole in its centre, due to the drilled mirror. Use this collimated image to align the components of the emission path.

4. Place the first mirror about 1 meter away from the objective exit; this removes most of the spurious reflections by the objective, which are not collimated.
5. Place the second non-polarizing cube after the mirror.
6. On the reflecting side of the non-polarizing cube, place a polarizing cube to separate 0° and 90° polarizations. On the transmitting side of the non-polarizing cube, place a polarizing cube rotated by 45° to separate the 45° from the 135° polarization.
7. Add four lenses of focal lengths 50 cm and focus by eye the four images of the rod into the four different APD's.
8. Add the Berek compensator and the band-pass filter before the first mirror, making sure that the whole collimated image of the nanorod is centred with regards to their aperture.
9. After having roughly aligned the mirrors and cubes using the collimated image of a rod, add two pinholes aligned with the beam axis. Add a small alignment laser beam going through those pinholes and use the back-reflection of the cubes and lenses to precisely adjust their orientation.

B. Fine adjustment of the mirrors and APD's position.

1. Acquire the voltage of each APD.
2. Roughly position the illuminated gold nanorod in the centre of the excited area.
3. Adjust the position of the first APD until the signal is maximized.
4. Adjust the position of the gold to maximize the signal.
5. Iterate 3. and 4. until convergence. This ensures that the first APD is aligned with the position of maximum excitation in the field of view.
6. Adjust all mirrors reflecting the three other channels into the APD until each APD displays its maximum signal.

C. Adjustment of the Berek compensator.

The Berek compensator corrects the phase shift between s- and p- polarization which is introduced by the dichroic mirrors and affects the measurement of the 45° and 135° polarization.

1. Adjust the orientation wheel of the Berek compensator until rotating the dephasing wheel does not affect either the 0° or the 90° polarization signal. Fix the orientation wheel.
2. Find a freely rotating rod on the glass surface, they are typically non-specifically attached by the tip and display fast Brownian rotation.
3. Rotate the dephasing wheel of the Berek until the amplitude of variation of the 45° and 135° signals scattered by that rod become maximum. Note the position of the dephasing wheel.
4. Repeat step 3 ~10 times for 10 different freely rotating rods, the positions of the dephasing wheel obtained at each step should be very close. Average all measured values and fix the dephasing wheel at that position.

To assess the extent to which the Berek compensator successfully reversed any mixing of the 45° and 135° polarizations, we measured the extinction ratios of collimated, linearly polarized 633 nm laser beams propagating from the location of the objective exit pupil through the optics of each APD channel. Transmission coefficients, relative to the largest transmission observed at the same input polarization, are shown below. Extinction ratios were ~200:1 for 0° and 90° polarizations, ~220:1 for 135° and ~90:1 for 45°

	Relative intensity transmission per APD channel (%)			
Input polarization (°)	0	90	45	135
0	100.0	0.5	49.4	41.8
90	0.5	100.0	49.3	58.5
45	49.2	56.5	100.0	1.1
135	58.9	42.1	0.4	100.0

Supplementary Method S2: Analytical inference of the rod orientation

We use the formulae derived from Fourkas (Opt. Lett. 26, 211-213, 2001), however we symmetrize their expressions as a function of $I_0^{true}, I_{90}^{true}, I_{45}^{true}, I_{135}^{true}$ to use the information of all channels. In the following we drop the *true* index.

Symmetric formulae for the rod angles follow from Eqns. 4a-d in Fourkas' article. We define the polarization anisotropies

$$x = \frac{\mathrm{I}_0 - \mathrm{I}_{90}}{\mathrm{I}_0 + \mathrm{I}_{90}}\ ;\ y = \frac{\mathrm{I}_{45} - \mathrm{I}_{135}}{\mathrm{I}_{45} + \mathrm{I}_{135}}$$

and from them the radius *r* in an *(x, y)* plot

$$r^2 = x^2 + y^2.$$

The symmetric form for φ then follows directly from Eqns. 4a-d and the identity $I_0+I_{90} = I_{45}+I_{135}$ (each term equals the total intensity, represented in a different basis):

$$\phi = \frac{1}{2}\arctan\left(\frac{\mathrm{y}}{\mathrm{x}}\right) = \frac{1}{2}\arctan\left(\frac{I_{45} - I_{135}}{I_0 - I_{90}}\right)$$

.

Similarly, Eqns. 4a-d give

$$r = \frac{C\ sin^2(\theta)}{A + B\ sin^2(\theta)}$$

and thus

$$sin^2(\theta) = \frac{rA}{C - rB}$$

where *A, B, C* are functions of the objective numerical aperture (NA) defined in Fourkas' article.

Simulations (Supplementary Note S2) show that Fourkas' formula for φ is very accurate in the range of rod orientations that we selected for bearing experiments. However we found that corrections were required to recover estimates of ϑ from data, as follows. The symmetric Fourkas formula $\vartheta(r)$ is plotted in Fig. S2 for selected values of NA, illustrating that *r* has a maximum value $r_{max} = C/(A+B) < 1$ when $\vartheta = 90°$, due to capture of dipole radiation in the minority polarization at the periphery of the objective BFP.

Due to degeneracies in the relation between the orientation and polarization signal, the angle ϕ is analytically retrieved in the range [0, π] and θ in the range [0,π/2].

In order to get ϕ in the range [0,2π], we unwrap the signal so as to ensure continuity. This works without ambiguity as long as the nanorod angular trajectory does not come too close to the vertical, in which case we discard the data or only analyse the rotation speed by performing Fourier transforms of the raw intensities or anisotropies. For Fig. 1E we plotted ϑ in the analytically recovered range [0,π/2], neglecting the alternate possible values (π - θ) in the range = [π/2, π].

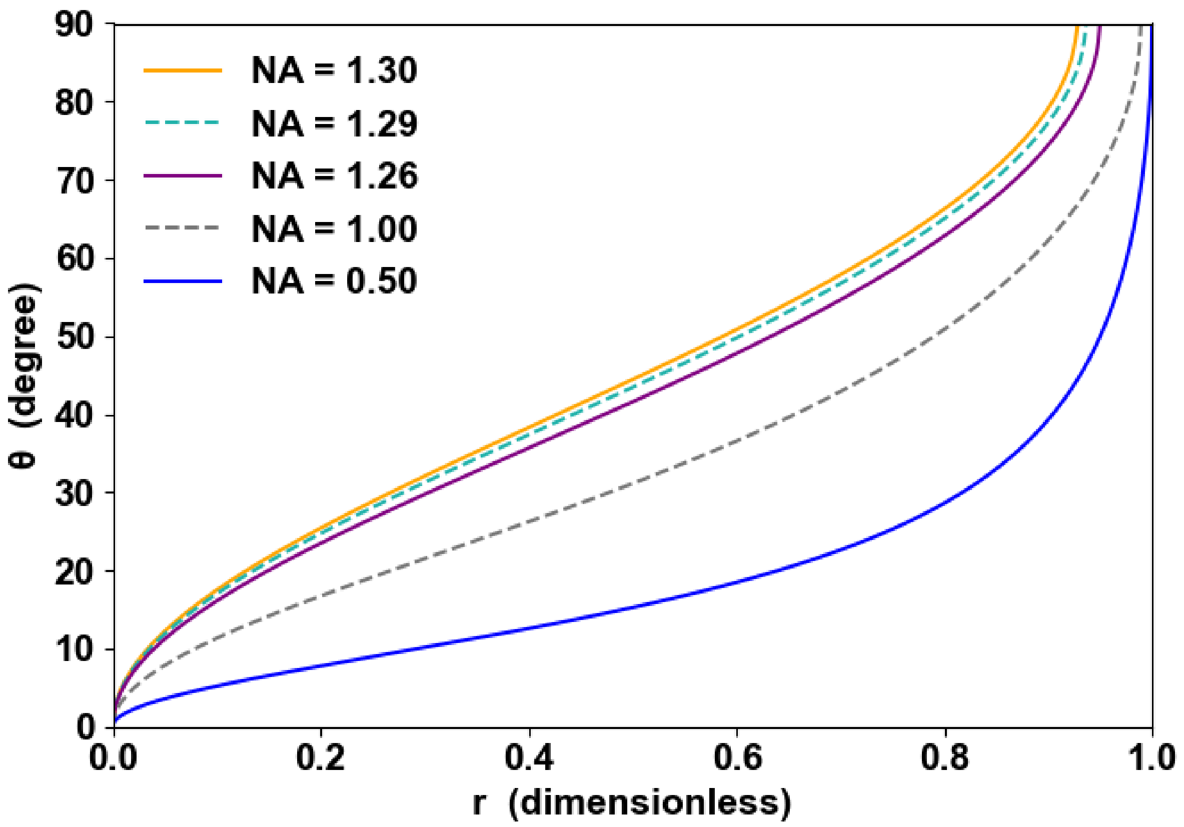

Figure S2: Analytical relationships between rod angle ϑ and magnitude of polarization anisotropy r for different objective NA

Using the nominal NA of our objective, 1.3, the largest experimental values of r gave $sin^2(\theta) > 1$ and thus were incompatible with real ϑ. We assume that the data recorded from our set of 21 different nanorods attached non-specifically to coverslips included data points where the nanorod angle passed through $\vartheta = 90°$, and that instrumental noise or inaccuracies of the Fourkas theory led to experimental values of $r > r_{max}$ at such extrema of ϑ. We therefore used, for all estimates of ϑ, an effective NA = 1.28671 that increased r_{max} to equal r^*, the 99.5th centile of experimental r values for a given nanorod, for the nanorod in our set of 21 where r^* was largest. Any data points where r still exceeded r_{max} after this correction were attributed to instrumental noise, and assigned as $\vartheta = 90°$. In future, for experiments where more accurate recovery of ϑ might be necessary, it might be possible to obtain a more accurate mapping from r to ϑ either via simulations similar to those of Fig S4, or via experimental calibration - using a method that remains to be devised to control nanorod angles. Here however none of our experiments require recovery of ϑ with more accuracy than the procedure described above.

Supplementary Method S3: Measurement of Instrumental Noise

To estimate the measurement noise due to the optics, we attach rods to the surface, infer φ as described in Supplementary Method 2, and inspect the power density function of the φ signal (Fig. S3a). The φ signal of such a rod integrated over a 1 Hz - 125 kHz measurement bandwidth displays a standard deviation from ~1° to ~0.4° at a working APD voltage, summed over the four APD's, of ~1 to ~12 V respectively (Fig. S3c).

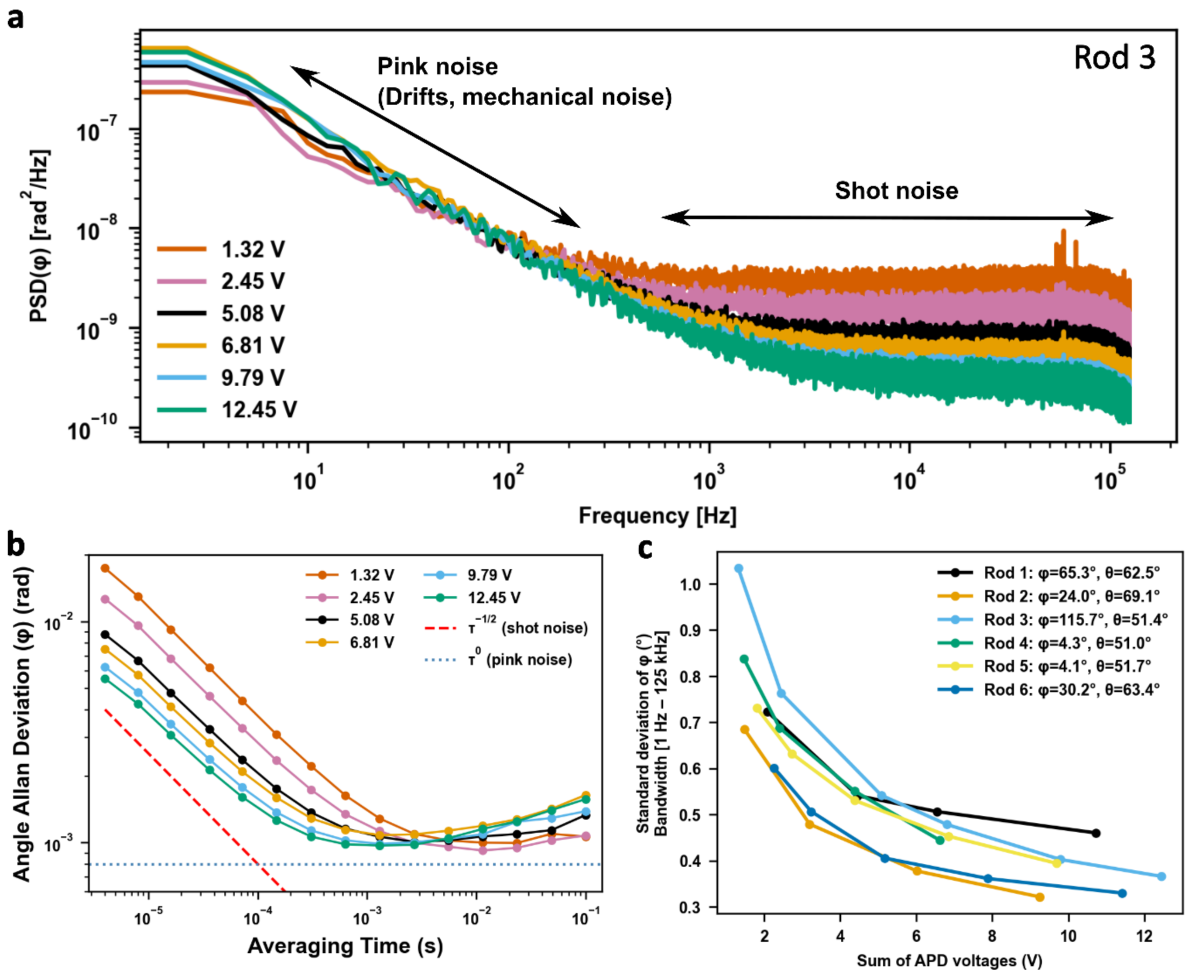

Figure S3: Measurement noise a. Power spectral density of the angular signal of a nanorod immobilized non-specifically on a glass surface, measured at different laser powers. The flat portion at high frequencies corresponds to the shot noise and depends on the light intensity received in the APD. The left portion, with exponent -1, is pink noise due to various drifts in the optical system. b**.** Allan Deviation for the data of *a*, with a $t^{-1/2}$ and t^{0} trend lines for comparison. c**.** Integrated noise in degrees as a function of the captured light intensity, modulated by changing the laser power, for the nanorod of *a* and 5 other similar rods. The labels indicate the average orientation of the rod.

Supplementary Note S1: Optical corrections due to cubes' imperfections

Each polarizing and non-polarizing cube has non ideal properties which affect the reconstitution of the polarization signal. To avoid any leak of the wrong polarization from each face of the polarizing cube, we glued linear polarizers on the exit face of each cube, aligned with the exit polarization corresponding to that face.

Then, the transmission of each polarization is carefully measured through the whole optical path by repetitive measurements and then averaging. We call t_0 the intensity transmission coefficient of a linearly polarized light through the whole 0° path and t_{90} for the whole 90° path.

For the 45° and 135° path, the effect of the cubes is made slightly more complicated by the coupling between all polarizations. In particular, even after compensation by the Berek compensator for phase shifts between 0° and 90° components, any differential transmission of these components to the 45/135 polarizing beam cube will alter the 45° and 135° signals. With a and b the *amplitude* transmission coefficient of the 0° and 90° polarization through all optical elements until the 45/135 polarizing beam cube, which are taken as real because their relative phase is cancelled by the Berek compensator, with t_{45} and t_{135} the *intensity* transmission coefficients through the 45/135 polarizing cubes of each polarization, and finally with $\alpha = \frac{a+b}{2}$ and $\beta = \frac{a-b}{2}$, we find that the relation between the true polarization components I^{true} and the measured intensities exiting the series of cubes I^{mes} is:

$$\overbrace{\begin{bmatrix} I_0^{meas} \\ I_{90}^{meas} \\ I_{45}^{meas} \\ I_{135}^{meas} \end{bmatrix}}^{I^{mes}} = \overbrace{\begin{bmatrix} a_0 & & & \\ & a_{90} & & \\ & & a_{45} & \\ & & & a_{135} \end{bmatrix}}^{D} \overbrace{\begin{bmatrix} t_0 & & & \\ & t_{90} & & \\ -\alpha\beta t_{45} & \alpha\beta t_{45} & \alpha^2 t_{45} & \beta^2 t_{45} \\ -\alpha\beta t_{135} & \alpha\beta t_{135} & \beta^2 t_{135} & \alpha^2 t_{135} \end{bmatrix}}^{A} \overbrace{\begin{bmatrix} I_0 \\ I_{90} \\ I_{45} \\ I_{135} \end{bmatrix}}^{I^{true}}$$

The matrix **A** represents the imperfection of the cubes while the matrix D accounts for the differential sensitivity of the APD's.

Then we compute I^{true} from I^{mes} by inverting this relation:

$$I^{true} = A^{-1} D^{-1} I^{mes}$$

While **A** is measured experimentally and corresponds to the cube properties, **D** is optimized by fixing a_0 to 1 and by minimizing the quantity $(I_0^{true} + I_{90}^{true} - I_{45}^{true} - I_{135}^{true})^2$ over a measurement of a nanorod in focus. This optimisation ensures that $I_0^{true} + I_{90}^{true}$ is as close as possible to $I_{45}^{true} + I_{135}^{true}$, which is a physical constraint. Note that this post-processing not only corrects the possible APDs' biases but also any error of measurement of the t_i.

Supplementary Note S2: Ray-optics simulation of the effect of a hole in the mirror.

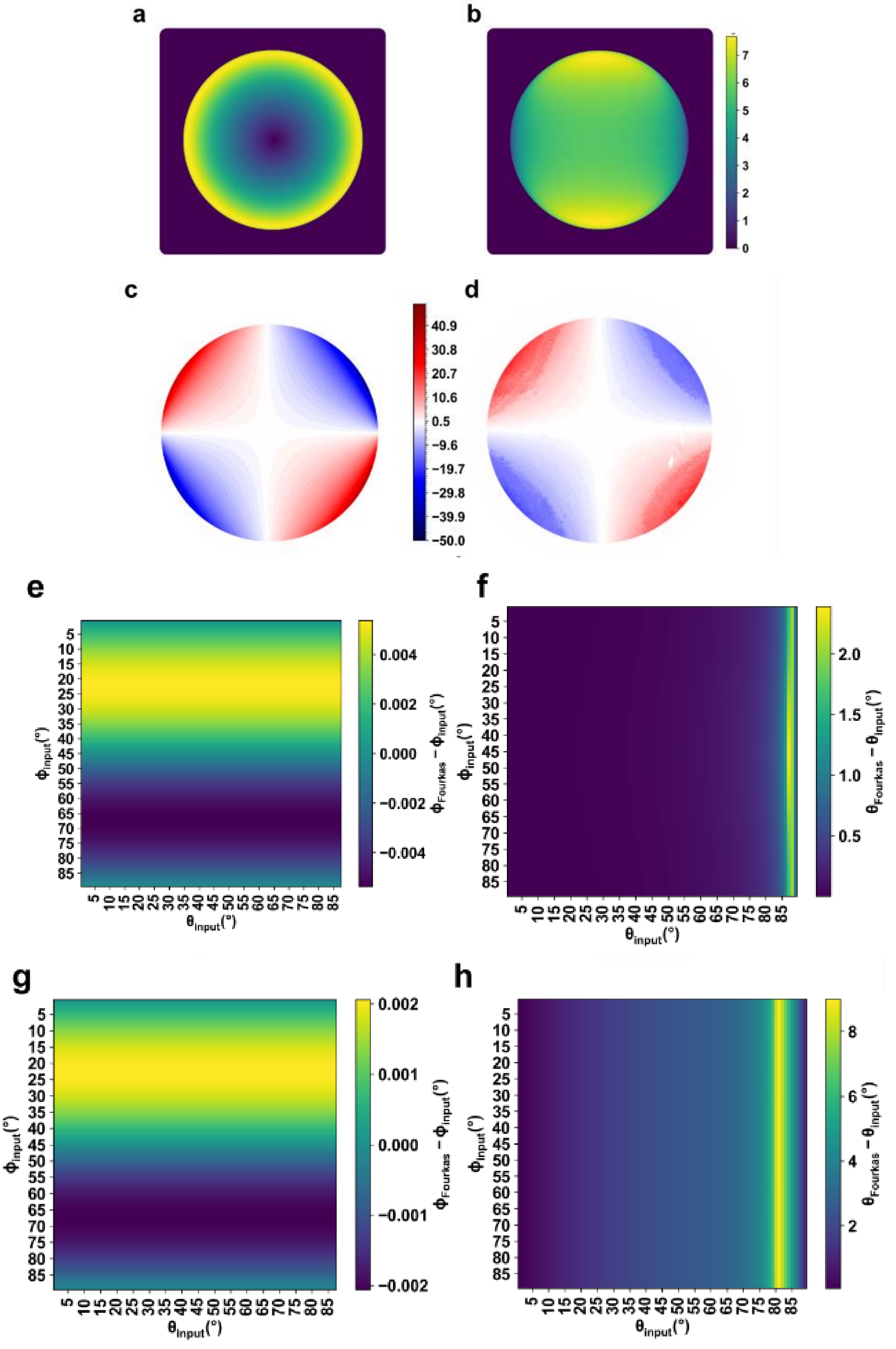

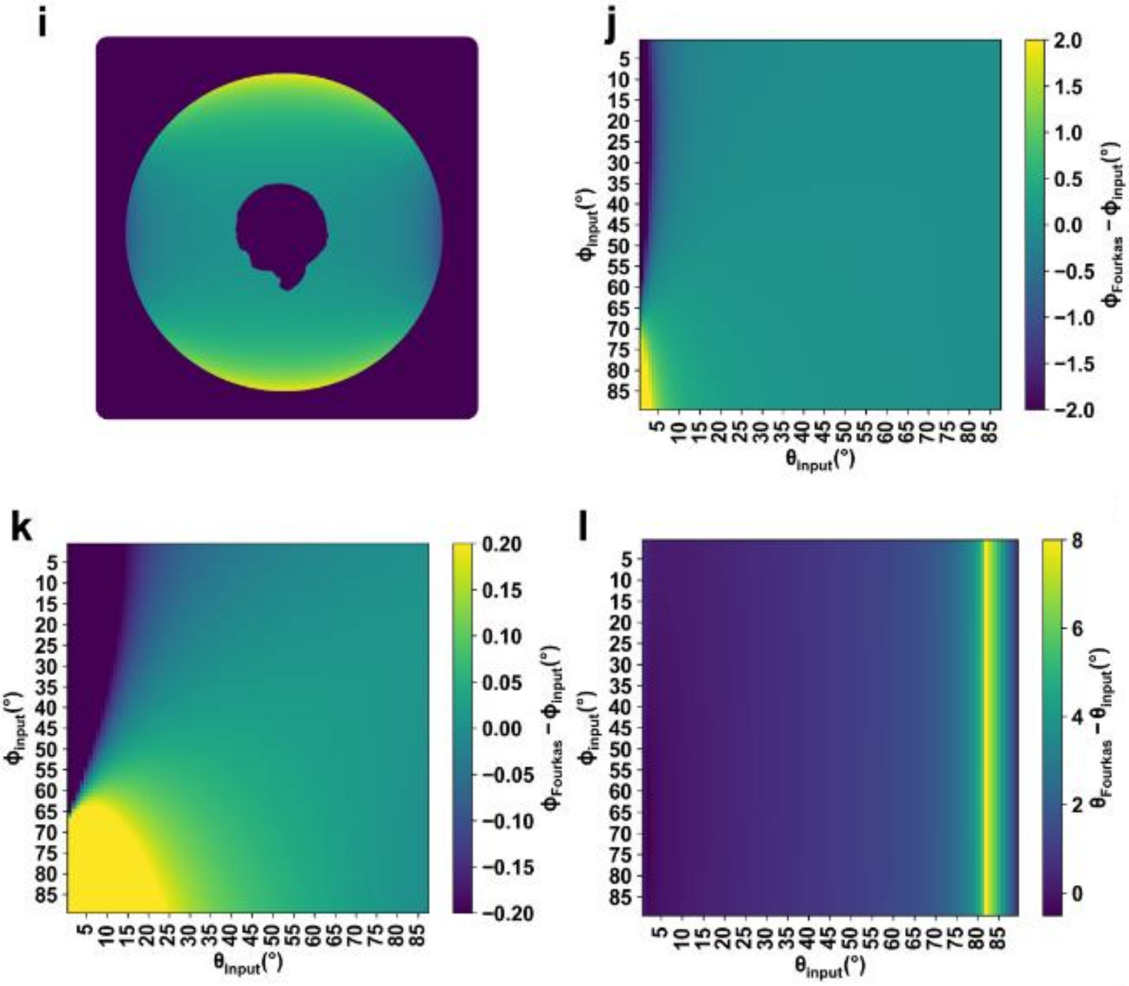

Figure S4: Simulation of the radiation of a dipole in focus as observed in the back-focal plane of our objective. Circles in (a-d, i) cover the full 1.3 NA of the objective back-focal plane. Refractive indices of water and immersion oil =1.33, 1.51 respectively. **a-b.** Intensity profile in the back focal plane (arbitrary units). **a**: θ = 0°, **b:** θ = 90°. **c**: Simulated orientation (in degrees) of the electric field in b as a function of the position in the back focal plane. **d.** Polarization direction measured in the back focal plane of the objective by putting a linear polarization filter in focus. **e.** Difference between φ used as input in the simulation and the retrieved φ after applying Fourkas formula to the integrated intensities in the back focal plane, as a function of φ and θ used as simulation input. **f.** Same as e. but for θ. **g-h.** Same as e-f but adding in the simulation the effect of refraction on polarization at the water/glass interface. **i.** Same as b. but superimposing the measured hole in the mirror to the BFP. All data in this figure were taken with this particular mirror and hole, which was modelled in all simulations. **j-k-l**. Same as g-h in the case of the hole in the back focal plane. **j** and **k** only differ by the angle range used in the colour bar. Deviations are less than 1 degree except in θ very near θ= 90° (horizontal rods) and in ϕ very near θ= 0° (vertical rods).

Since we cannot orient at will a gold nanorod, we do not have a simple experimental method to verify the accuracy of the orientation reconstitution from the four polarization channels. However, to get an intuition of how the hole in the mirror might impact the signal reconstitutions we ran simple ray optics simulations corresponding to the analytical method of Fourkas. We use the far-field dipole radiation formula $\vec{E} \propto \vec{r} \times \vec{D} \times \vec{r}$, where **D** is the orientation of the rod and **r** the unit vector

corresponding of a ray. We then attribute each ray to a position in the back focal plane (BFP). We add a mask in the BFP corresponding to the drilled hole in the mirror (measured by imaging the mirror plane) and integrate the four polarizations over the remaining BFP. Then we apply Fourkas' formula over those integrated polarizations and check that we retrieve the orientation of the originally defined **D(θ, φ).** Figure S4 shows the result of the simulation of the dipole radiation as inferred in the back focal plane of the objective. It also shows the difference between the input orientations ϕ and θ, and the corresponding inferred orientation after integrating the intensities in the four polarizations in the BFP and applying on them the symmetrized Fourkas' formula indicated in Supplementary Method S2. The difference lies well below one degree (~0.001°), except in the case of a rod very close to the horizontal (θ ~ 90°), which proves the correctness of the simulation and its agreement with Fourkas' analytical formula. We then add Fresnel refraction at the water-glass interface and show that it doesn't have a very strong effect. We finally superimpose a mask corresponding to the true shape of the hole in our mirror onto the BFP before integrating. The simulation shows small errors compared to the noise of typical magnitude of the angular data observed in our data. Overall, while this is not an experimental proof, the simulations suggest that the hole in the mirror will have a negligible effect on the measurement and inference of the rod orientation.

Supplementary Note S3: Experimental calibration of recovered nanorod angle

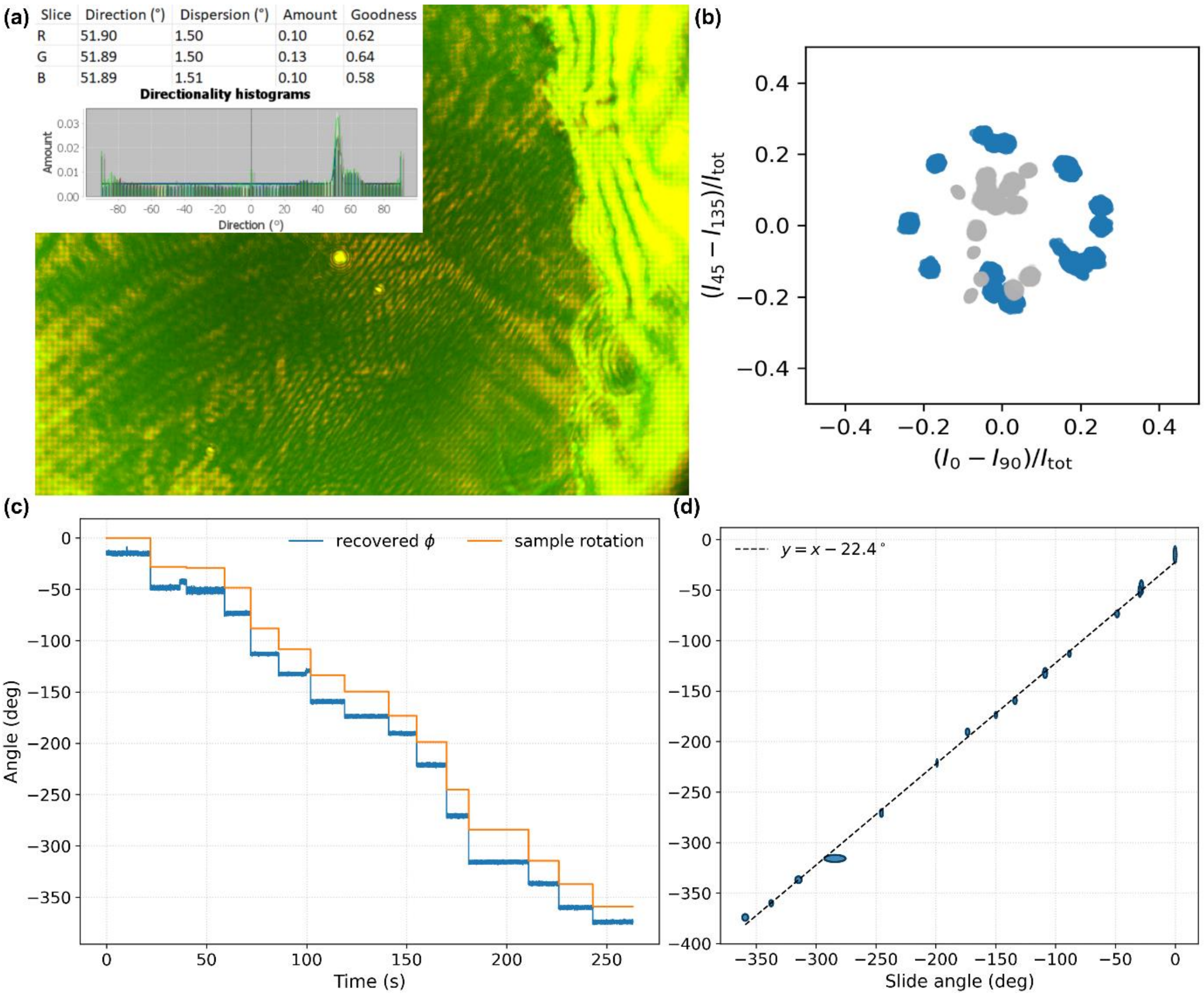

Figure S5: Calibration of recovered rod angle. a. Image of a gold nanorod attached to a coverglass marked to allow location and re-centering of the same rod after manual rotation of the sample. For these images the sample was illuminated with LED and wide-field laser and over-exposed to reveal features that allow the orientation of the entire sample to be determined. Inset: output from the "Directionality" function of ImageJ that was used to estimate sample rotation angle from each image. 14 such images were taken, one for each angle. **b.** In the polarization anisotropy plane, data from the fixed nanorod of **a** (blue) form a clear double-circular trajectory, consistent with rotation about the optical axis, with scatter that is probably due to imperfect re-centering and re-focussing of the nanorod after each rotation and/or asymmetries in the sample slide and coverslip. A non-nanorod scattering object (grey) shows only scatter. Wide-field and LED illumination were absent for nanorod angle recordings. **c.** Concatenated plot of angles for the nanorod of **a** versus recording time. The azimuthal angle φ recovered using the Fourkas reconstruction formula (blue) tracks the applied sample rotation (orange) closely. **d.** Recovered φ versus sample rotation angle is well described by a linear relation with unit slope. The offset 22.2° is the angle φ at which this particular nanorod was stuck, relative to the orientation of the main features of the coverslip that are used to measure the rotation of the image. Error ellipses show uncertainty in the slide rotation inferred from the ImageJ analysis and the full range covered by the recovered angle signal.

To assess the accuracy of nanorod azimuthal angle φ recovered from the four polarization channels with the hole mirror using the analytical method of Fourkas, we performed an experimental calibration based on controlled rotation of a fixed nanorod. A gold nanorod was immobilized non-specifically on a glass coverslip that had been marked with ink and scratches from a diamond pen, allowing the same nanorod to be relocated after manual rotation of the sample to a range of angles. After each rotation, the nanorod was re-centred and re-focussed as accurately as possible within the APD excitation region and a short polarization time series was recorded.

The true nanorod angle relative to its starting point (φ; ϑ remains constant) was estimated by measuring rotation of the entire sample using the “Directionality” function in ImageJ to recover the orientation of prominent features in images of the nanorod and its surroundings (Fig. S5a, c). Recovered nanorod angle was obtained using Fourkas’ formula, exactly as for nanorods attached to bacterial flagella (Fig. S5b-d). Recovered angles were very close to true angles. Errors due to imperfect re-centering and re-focussing of the nanorod after each rotation and/or asymmetries in the sample slide and coverslip are not negligible, as indicated by the anisotropy plot for a nanorod and another scattering object (Fig. S5b, blue, grey respectively) – these likely dominate the observed deviations from perfect angle recovery in this calibration experiment.

Supplementary Note S4: Estimation of heating due to the nanorod absorption

The nanorods used in this study have diameter of 40 nm and a length of 68 nm. We can define their effective radius $R_{eff} = \left(\frac{3V}{4\pi}\right)^{\frac{1}{3}} \simeq 30\,nm$ and their aspect ratio $\frac{L}{D} = 1.7$. For those dimensions, Park et al.[1] have measured that the absorption cross-section of gold nanorods is of the same order of magnitude as the scattering cross-section, *i.e.* that at a given laser power, the nanorods will absorb as much heat from the laser as they scatter.

We can estimate the power scattered by the rod in our conditions. Roughly half of the light scattered by the rod goes into the objective, and half of that light goes to the APD's (the other to the camera). We work at a typical voltage, summed of the 4 APD's, of 4V, so that given the photoelectric sensitivity of the APD of $\epsilon = 1 \times 10^8 V/W$, we can estimate the total power scattered, and similarly absorbed, by one rod to be $P_{abs} = 0.15\mu W$.

To simplify, we model a gold nanorod as a sphere of radius a = 30 nm. In that case, the steady-state temperature profile around it is a solution of the 3D Poisson equation $\nabla^2 T = 0$, thus is equal to $T(r) = T_{+\infty} + T_e \frac{a}{r}$. The total thermal flux, which corresponds in the steady state to the absorbed light is thus $4\pi T_e a\lambda = P_{abs}$, with $\lambda = 0.6\, W/m.K$ the thermal conductivity of water. Thus $T_e = \frac{P_{abs}}{4\pi\lambda a} \simeq 0.75\, K$.

Thus, the heating due to the nanorod absorption cross-section does not seem to be critical. However, one should be aware that either an increased absorption coefficient (which could be caused by a smaller aspect-ratio of an individual rod), or a slightly larger laser power would bring the temperature increase to a range of a few Kelvins. Caution should be exerted while choosing those parameters.

We assessed laser heating experimentally (Fig. S6). We measured the rotational diffusion of gold nanorods non-specifically attached to a glass coverslip as a function of excitation power. Significant heating would reduce the viscosity of water and thus increase the rotational diffusion coefficient and correspondingly the diffusion parameter

$$b = \frac{k_B T}{2\pi^2\, \eta(T)\, \alpha}$$

where η is viscosity and α is the geometric viscous drag factor for each nanorod depending on its size and orientation (the diffusion coefficient D = $2\pi^2\, b$). For each nanorod and excitation power we pooled several 10 s segments and computed a median value of *b* by fitting the powerspectrum of φ as

$$S_\varphi(f) - S_0 = {}^{b}/_{f^2}$$

Where S_0 is the shot noise and *f* the frequency (Fig. S6b, c). Laser induced heating would increase *b* via both the direct dependence on *T* and the reduction in viscosity. The latter factor is dominant, with ~10% decrease in viscosity for a 5 °C temperature rise at room temp. There was no systematic decrease in *b* as laser power was reduced nearly 10-fold from 180 µW (a typical value in our flagellar experiments is 40 µW), indicating minimal heating at 180 µW.

To place a conservative upper bound on heating, we normalized each rod by its own low-power value b_0 (= *b* at 20 µW) and fitted a linear dependence of b/b_0 on power using the 60 and 180 µW points. The upper 95% confidence bound of this trend gives an upper limit $\Delta(b/b_0) \leq 0.02$ from 20 to 180µW (Fig. S6d).

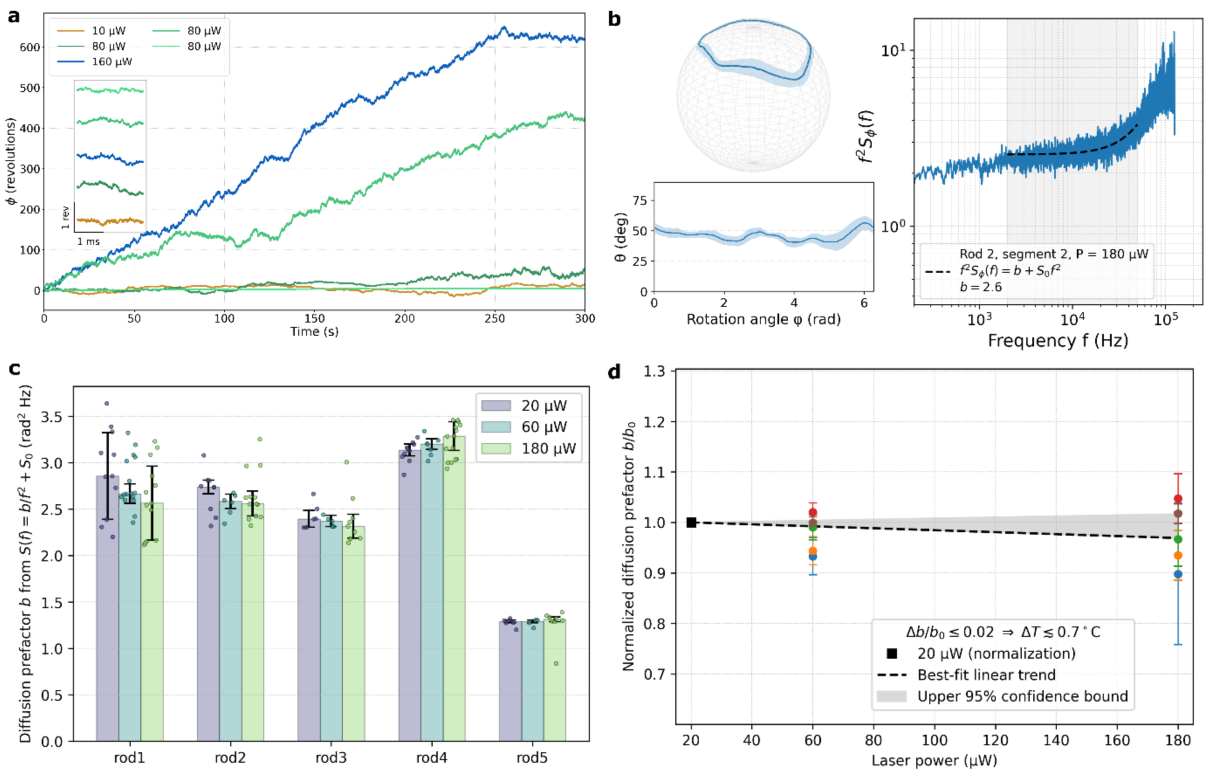

Figure S6: Estimation of possible laser heating via Brownian Motion of rotating nanorods loosely surface-attached. **a.** Long traces of the azimuthal angle ϕ of a streptavidin gold nanorod ("rod 0") non-specifically stuck to the coverglass, recorded at three laser powers. 160µW is at the top of the range used for the bearing experiments. At high power optical torque drives rotation at a few Hz. Repeats at 80 µW differ substantially, indicating slow changes in attachment. To avoid such long-term drift, for rods 1–5 we used faster power switching (several ~10s segments at each power). **b.** Brownian rotation of rod 2 over a single 10s segment at 180 µW. Left: reconstructed orientation on the unit sphere and the corresponding ϑ–ϕ relationship; mean (dark blue) ± SD (light blue) of ϑ for each 3.6° bin of ϕ. Right: power spectral density of ϕ multiplied by frequency-squared, versus frequency; running average of 10 consecutive points (blue) and best fit to $b + S_0F^2$ over the range 2 kHz-50 kHz (black). Up to ~10 kHz the plot is approximately flat, consistent with free Brownian rotation. At higher frequencies a rising trend reflects the shot-noise floor S_0; at low frequencies a downward trend likely reflects slow variations in rod–surface coupling. **c.** The diffusion parameter b, estimated from fits such as b for 5 different nanorods at three laser powers. Bars indicate the mean value of *b* across segments for each rod and power, and error bars show the median absolute deviation. **d.** Normalized diffusion parameter b/b_0, where b_0 is the 20 µW value for each nanorod from c. The dashed line is a linear fit to the normalized data; the grey region shows the upper 95% confidence bound of the fit. This yields a conservative upper bound $\Delta(b/b_0) \leq 0.02$, corresponding (via the temperature dependence of water viscosity) to $\Delta T \leq 0.7$ °C between 20 and 180 µW.

Given $(b/b_0) = T/\eta(T)$

$$\frac{d}{dT}\left(\ln\left(\frac{b}{b_0}\right)\right) = \frac{1}{T} - \frac{d}{dT}(\ln \eta)$$

Near room temperature the second term dominates and $\frac{d}{dT}\left(\ln\left(\frac{b}{b_0}\right)\right)$ = 0.023 K^{-1}. Therefore our upper limit of $\Delta(b/b_0) \leq 0.02$ corresponds to a maximum temperature rise of ~0.7 K, comparable to the values < 1 K calculated theoretically, above.

Supplementary Note S5: Estimation of torque exerted by the circularly polarized light

The optical torque exerted at the plasmon resonance for slightly larger rods has been previously estimated to lie around $10\ \mathrm{pN.\mu m}/(W.\mu\, m^{-2})^2$. Our laser beam in focus has a Gaussian shape of deviation σ = 1.4 μm. The typical laser power used in our experiment (as measured with a power meter placed above the glass slide) is 40 μW. Thus, the maximum light intensity at the centre of the beam reaches 3 μW/μm^2. Using the estimation above, this corresponds to a torque of 0.03 pN.nm, which is negligible in comparison with the typical torque per stator of the bacterial flagellar motor of 200 pN.nm [3]. Nonetheless, based on the rotational drag measured from the rotation of nanorods non-specifically attached to the glass (Fig. S7), we estimate that circularly polarized light should bias the rotation of unhindered rods by approximately 10 revolutions per second. This is somewhat greater than the bias observed in non-specifically attached rods exploring the full angular space, which is a few revolutions per second (e.g. Fig. S6a) .

Supplementary Note S6: Rotational drag of rods non-specifically linked to a glass surface.

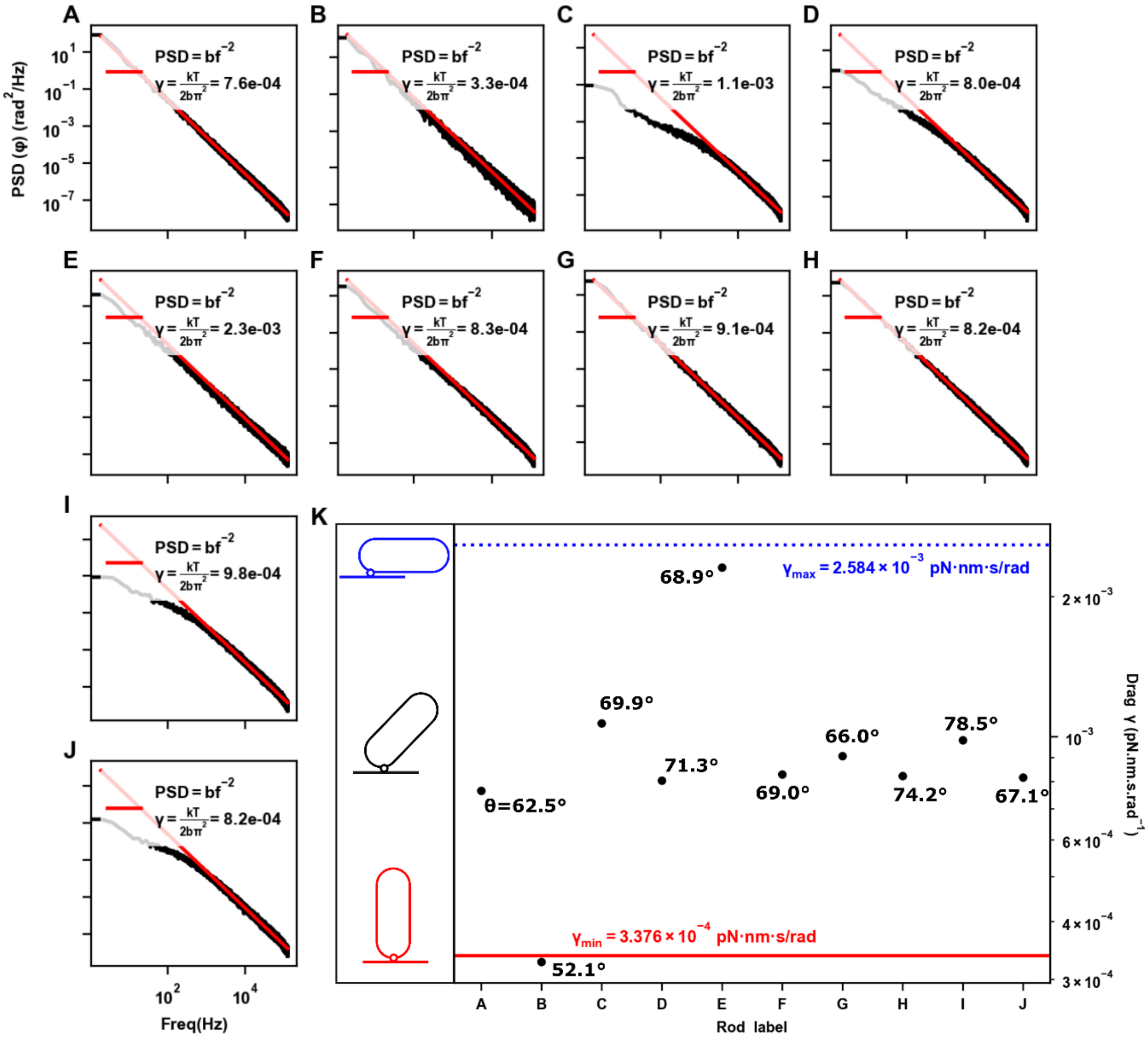

Figure S7: Power spectral density and inferred rotational drag of 10 loosely surface-attached rotating rods. a–j: Power spectral density of angular motion for each rod, with $b \times f^{-2}$ fits shown at high frequencies. Axis ticks are consistent across all panels and labelled in j. **k**: Summary of inferred rotational drag coefficients, which fall between theoretical estimates of the upper and lower bounds, calculated using formulae from Macromolecules 42, 16, 6290–6299 (2009) with corrections for the proximity of the surface from Brenner, H. Appl. sci. Res. 13, 81–120 (1964). The theoretical bounds were calculated using the rod geometries shown in corresponding colours, left. Lower limit (red): nanorod long axis vertical, attached by 5 nm linker (streptavidin coated nanorods), rotating about its long axis. Upper limit (blue): nanorod long axis horizontal, attached by 5 nm linker at the extreme end of its cylindrical portion, rotating about an axis perpendicular to the surface passing through the linker. Rotating nanorods typically attach with intermediate geometries and drag coefficients (black, ϑ in the range ~50-80°). The outlying drag coefficients of rods B and E are not attributable to their orientation, but may reflect variations in rod dimensions or surface attachment.

We measured the rotational drag of ten gold nanorods which were undergoing Brownian motion while being non-specifically attached to the glass surface. We expected that the PSD of ϕ would decrease as $\frac{1}{f^2}$, with f the frequency. We show their power spectrum density in Fig. S7. Over the ten gold nanorods, six display such an expected spectrum while four others seem partially trapped at a preferred angular position and only display the -2 exponent at high frequency, a region which we use to infer the drag. We find that drag values accumulate around $8.10^{-4}\,pN.nm.s.rad^{-1}$, however with a large variability, some rods displaying drags as large as $1.10^{-2}\,pN.nm.s.rad^{-1}$. Series development of the rotational drag based on boundary element computations[4,5]predict the rotational drag of our 68x40 nm rods to be in the range $\gamma_{\perp} = (3.3 - 26)\ x\ 10^{-4}\,pN.nm.s.rad^{-1}$, which is the same order of magnitude as our experimental values (Fig. S7). The major contributor to the variation in drag coefficient is likely to be rod orientation (Fig. S7). Electron Microscopy of similar nanorods from the same manufacturer revealed some variability in rod dimensions, which will also contribute to variable drag coefficients. As a comparison, previous studies used 60 nm[6] and 100 nm[7] gold nanospheres, which have total rotational drag coefficients similar to those measured here (typically from 0.001 to 0.005 $pN.nm.s.rad^{-1}$).

Supplementary Note S7: Estimation of Barrier Heights Using Transition Times and Drag Coefficients

Kramers' formula for transition rates over energy barriers in a smooth parabolic potential, mirrored at the peak of the barrier, is a widely used tool in biophysics to estimate energy barrier heights from transition times and drag coefficients. In our study, we estimated the rotational drag coefficient for gold nanorods by combining theoretical approximations and experimental measurements of nanorods attached non-specifically to a glass surface (Supplementary Note S6). The rotational drag was found to be approximately $\gamma = 8.10^{-4}\,pN.nm.s.rad^{-1}$albeit with significant variability due to differences in nanorod shapes and orientations.

Kramer's formula[8] for a mirrored parabolic potential where two minima are separated by a distance δ is

$t = \frac{\sqrt{\pi}\gamma\ \delta^2 \sqrt{T}}{4\,U^{3/2}}\ e^{U/(k_B T)}$ (see reference above, p 239; we express the curvature of the potential in the original, ω, in terms of the barrier height U (Q in the original) and the separation between states δ, and modernize the notation of temperature and viscous drag coefficient).

For the typical transition time 0.1s found in this study between states separated by δ = 1/26 rev, we find an energy barrier of 14 k_BT. If we now take the largest drag coefficient measured on a freely rotating rod ($\gamma = 1.10^{-2}\,pN.nm.s.rad^{-1}$), we find an energy barrier of 11 k_BT.

Despite its utility, several limitations apply to the use of Kramers' formula in estimating barrier heights. Kramers' formula assumes a smooth parabolic potential near the barrier. This approximation greatly simplifies the relationship between the barrier height and the transition times. However, the exact shape of the energy landscape can significantly alter the dynamics. For instance, a squeezed barrier—where the potential narrows near the top—can increase transition rates by reducing the time spent near the barrier. Such deviations from the assumed parabolic shape lead to estimates that reflect an effective or equivalent barrier height rather than the true microscopic energy barrier. Furthermore, in a landscape accurately described by Kramers' model, transition times are expected to follow an exponential distribution. Our experimental data, however, exhibit a clear deviation from this distribution. This discrepancy indicates that the true energy landscape underlying the transitions is more complex than the idealized Kramers potential.

Supplementary Figures S8-S14 show Extended Data…

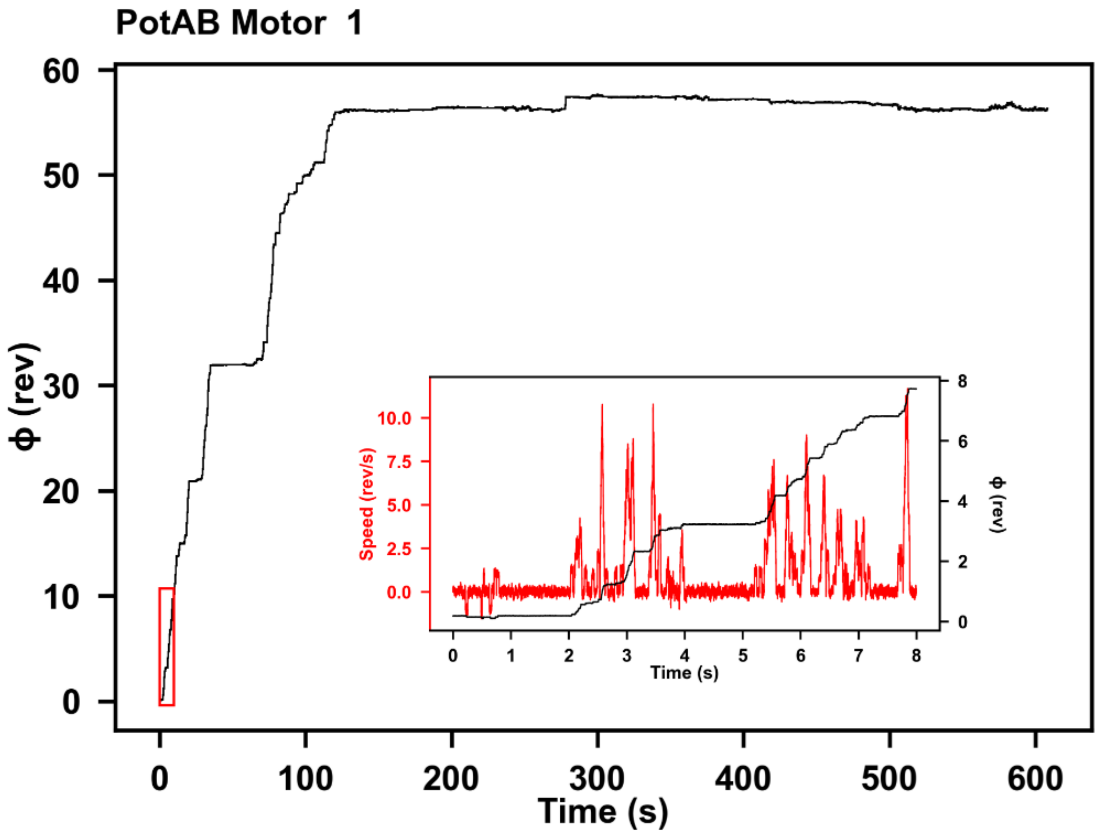

Figure S8: Angular trajectory of PotAB Motor 1 following buffer exchange (~0 mM NaCl). Inset: Zoomed-in view showing angular position (black) and speed (red), with speed derived as the time derivative of angle and smoothed over a 40-ms running window.

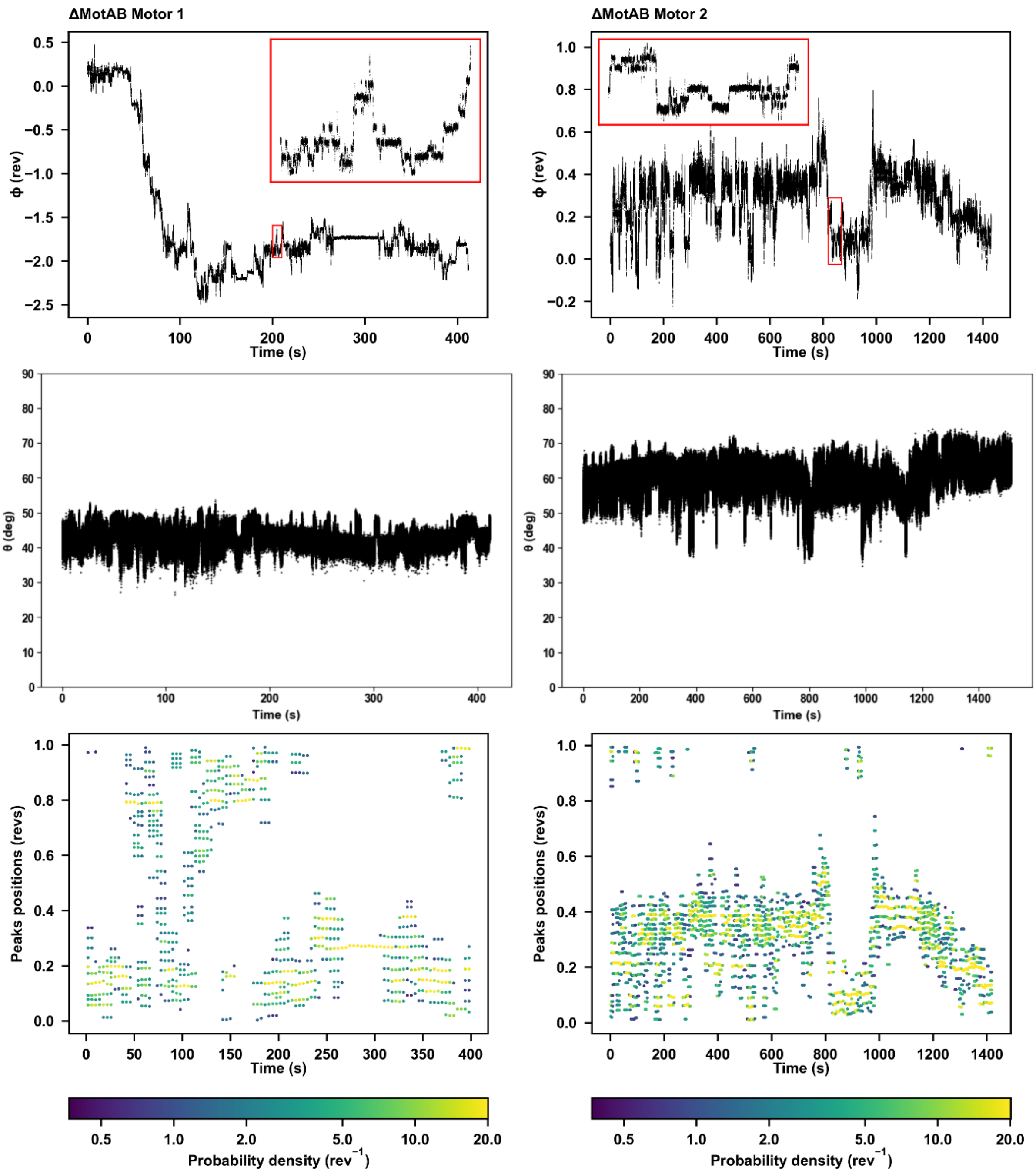

Figure S9: Diffusion dynamics of ΔMotAB Motors 1 and 2. Top: Motor angle φ versus time trace. **Middle:** Nanorod angle ϑ versus time. **Bottom:** Evolution of peak positions in angular histograms computed over overlapping 10 s windows, shifted every 4 s. Colour bar indicates the probability density (rev^{-1}) at each peak. Lower peaks (dark blue) are more error-prone. See also supplementary videos S4, S5.

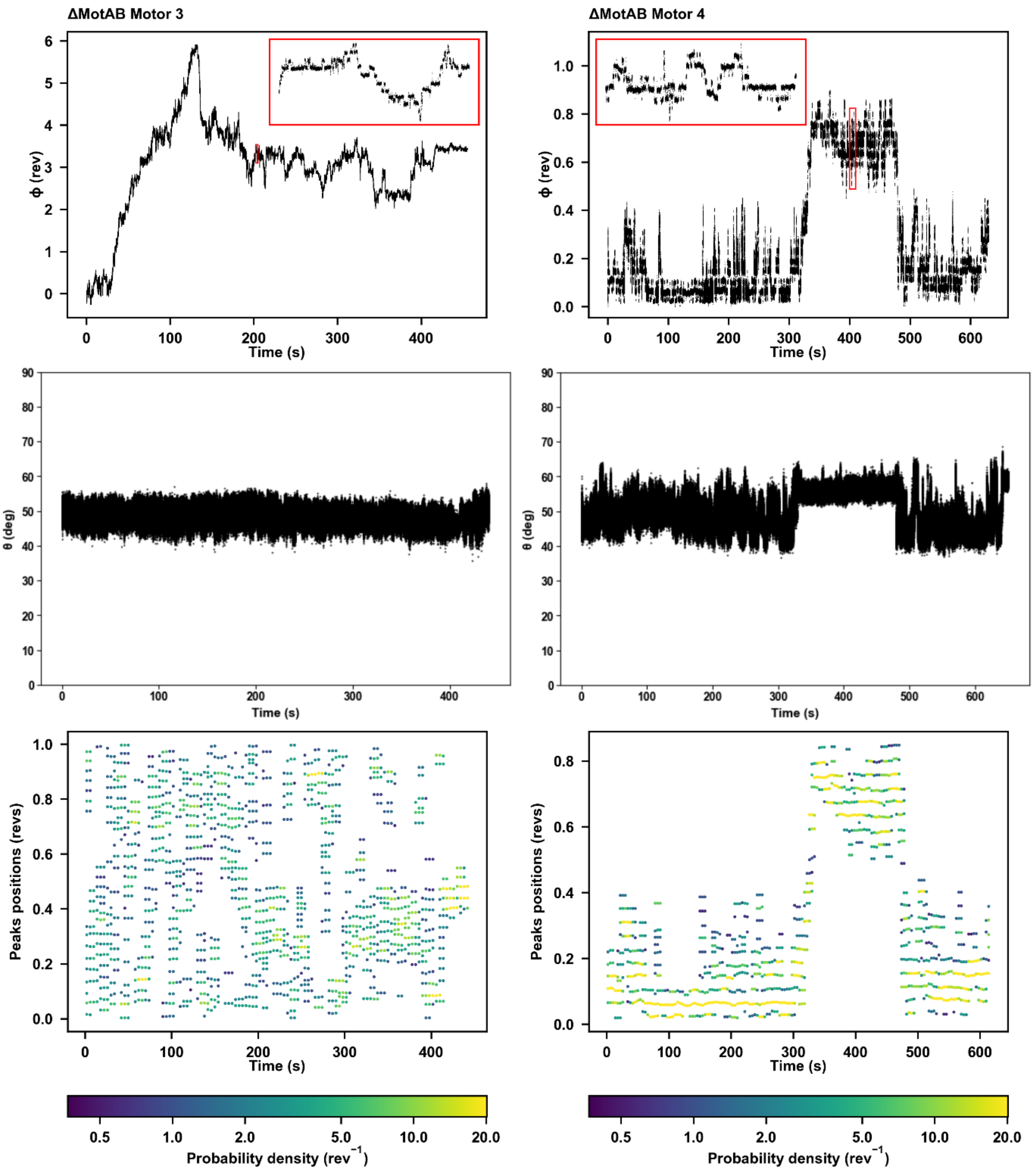

Figure S10: Diffusion dynamics of ΔMotAB Motors 3 and 4. Top: Motor angle φ versus time trace. **Middle:** Nanorod angle ϑ versus time. **Bottom:** Evolution of peak positions in angular histograms computed over overlapping 10 s windows, shifted every 4 s. Colour bar indicates the probability density (rev^{-1}) at each peak. Lower peaks (dark blue) are more error-prone. See also supplementary videos S6, S7.

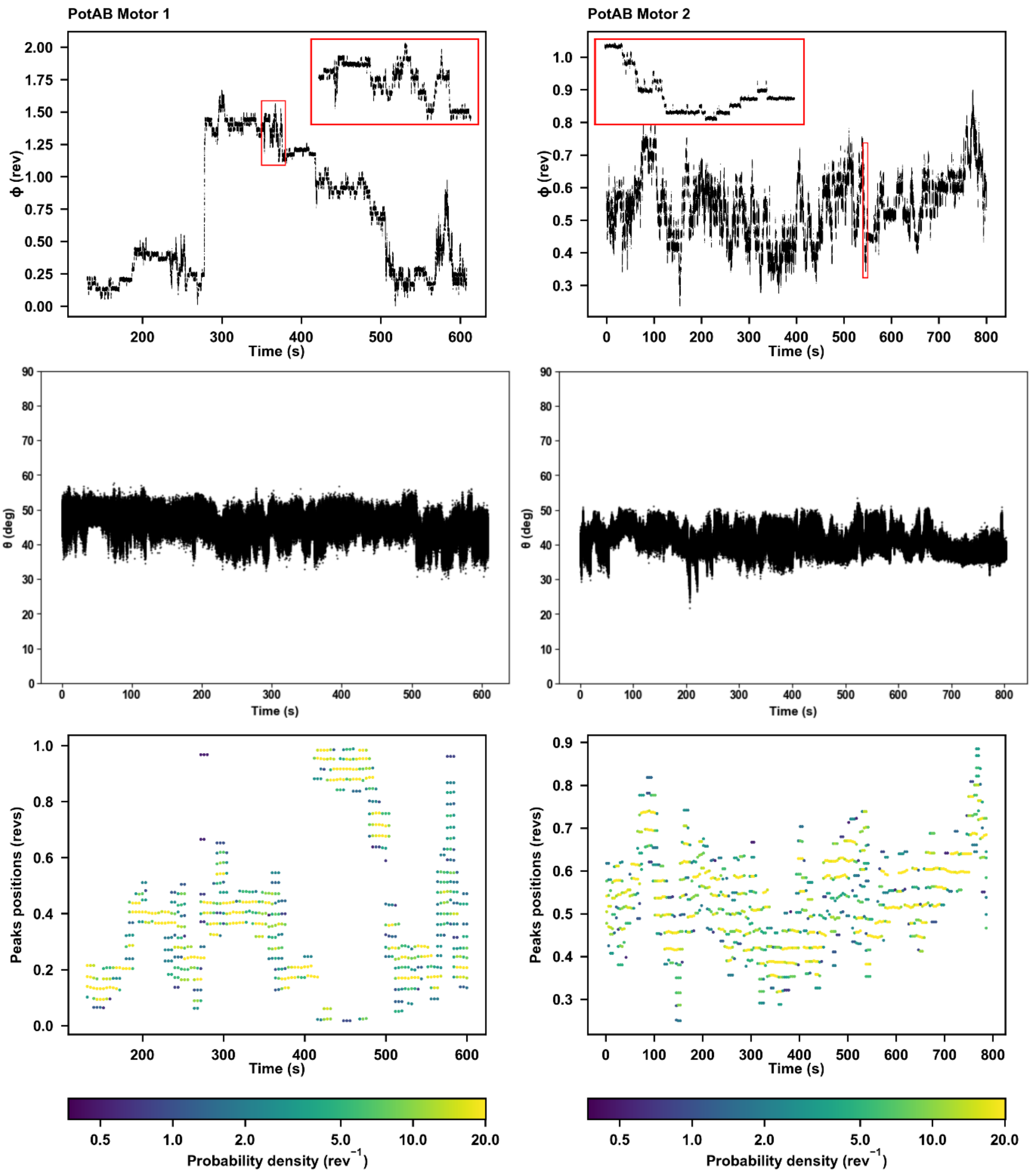

Figure S11: Diffusion dynamics of PotAB Motors 1 and 2. Top: Motor angle φ versus time trace. **Middle:** Nanorod angle ϑ versus time. **Bottom:** Evolution of peak positions in angular histograms computed over overlapping 10 s windows, shifted every 4 s. Colour bar indicates the probability density (rev^{-1}) at each peak. Lower peaks (dark blue) are more error-prone. See also supplementary videos S1, S2.

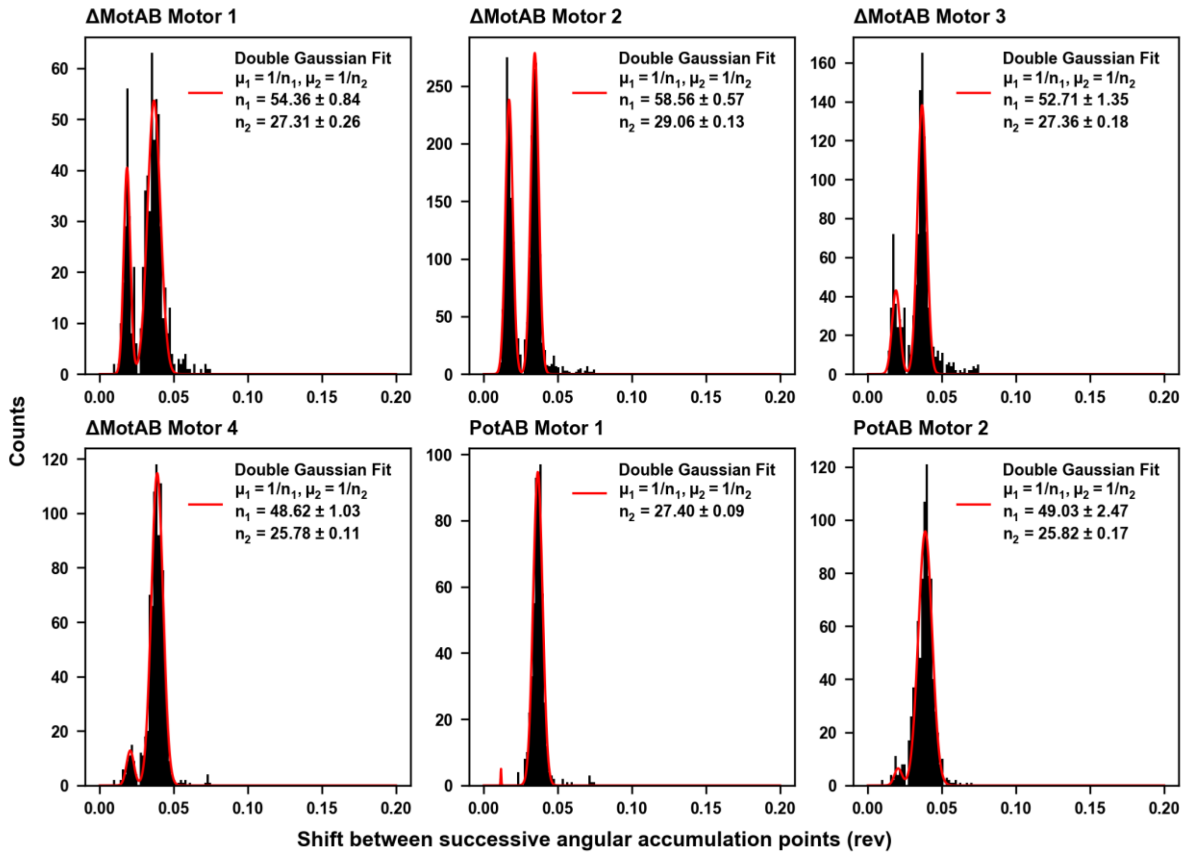

Figure S12: Distribution of angular shifts between successive accumulation peaks for individual motors. The histograms show the distribution of angular displacements between consecutive peak positions identified in angular histograms (see Fig. S9-11). In addition to the dominant peak separation close to 1/26 rev, several motors show a peak separation half this size, corresponding to smaller steps which are ignored by the step-finding analysis. Each panel corresponds to a single motor. Red lines represent double Gaussian fits with inverse peak positions (μ_1 = 1/n_1, μ_2 = 1/n_2) and associated fitting parameters.

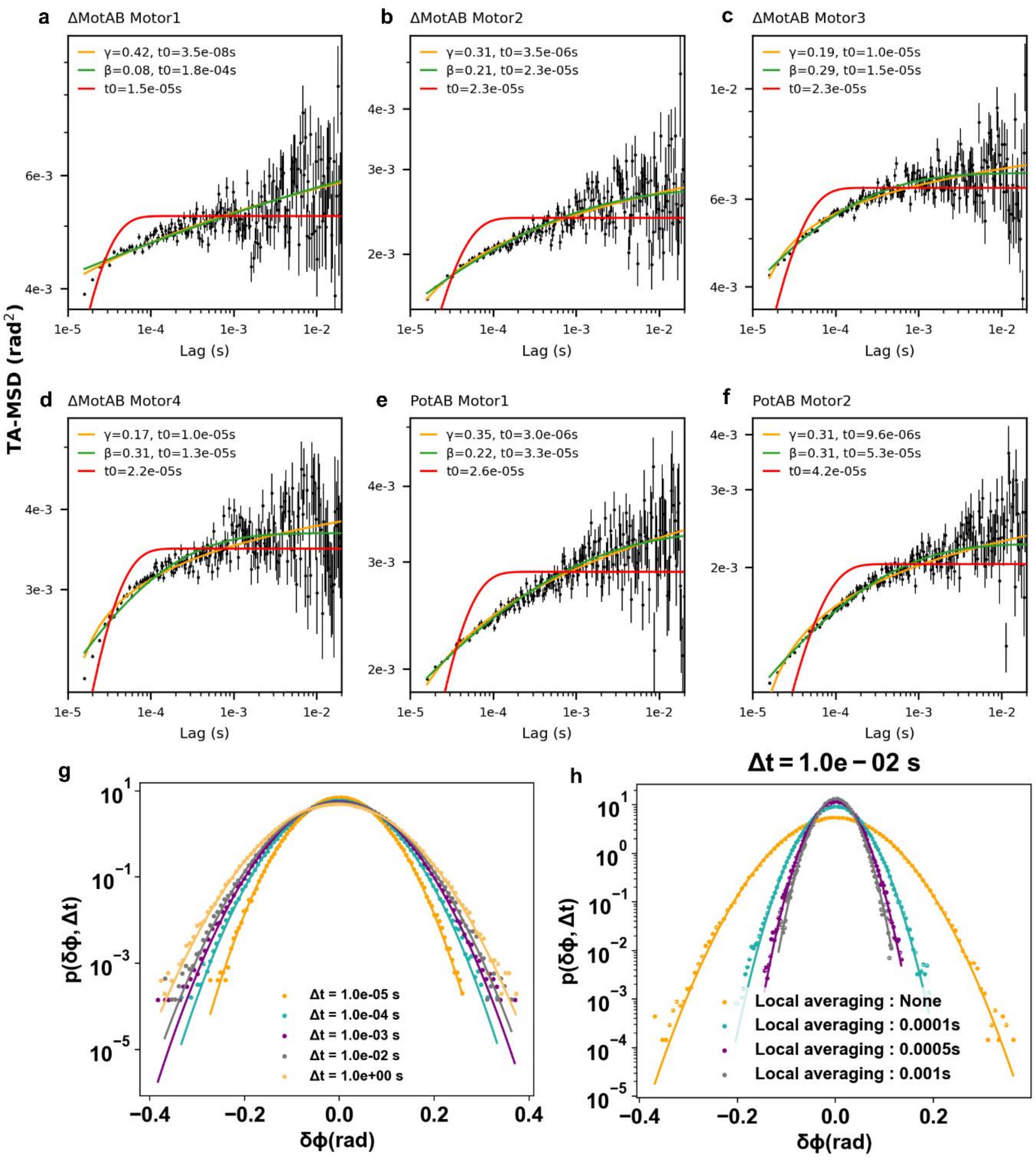

Figure S13: Angular fluctuation analysis during 1-second dwells. a–f: Time-averaged mean-square displacement (TA-MSD) during individual 1-second dwells (no steps), one per motor, fitted with exponential (red, $Ae^{-\frac{t}{t_0}}$), stretched-exponential (green, $Ae^{-\left(\frac{t}{t_0}\right)^{\beta}}$), and stretched-logarithmic (orange, $Alog^{-\left(\frac{t}{t_0}\right)^{\gamma}}$) relaxation. Error bars correspond to $\sigma/\sqrt{N_s}$, where σ is the standard deviation of the N_s independent MSD values for each lag. **g:** Probability distributions $p(\delta\phi, \Delta t)$ of angular displacements at increasing lag times Δt, showing Gaussian behavior across timescales (from the dwell analysed in e.). **h:** Angular displacement distributions at fixed lag time (Δt =0.01) under different levels of local averaging to remove high-frequency noise; Gaussian nature is preserved.

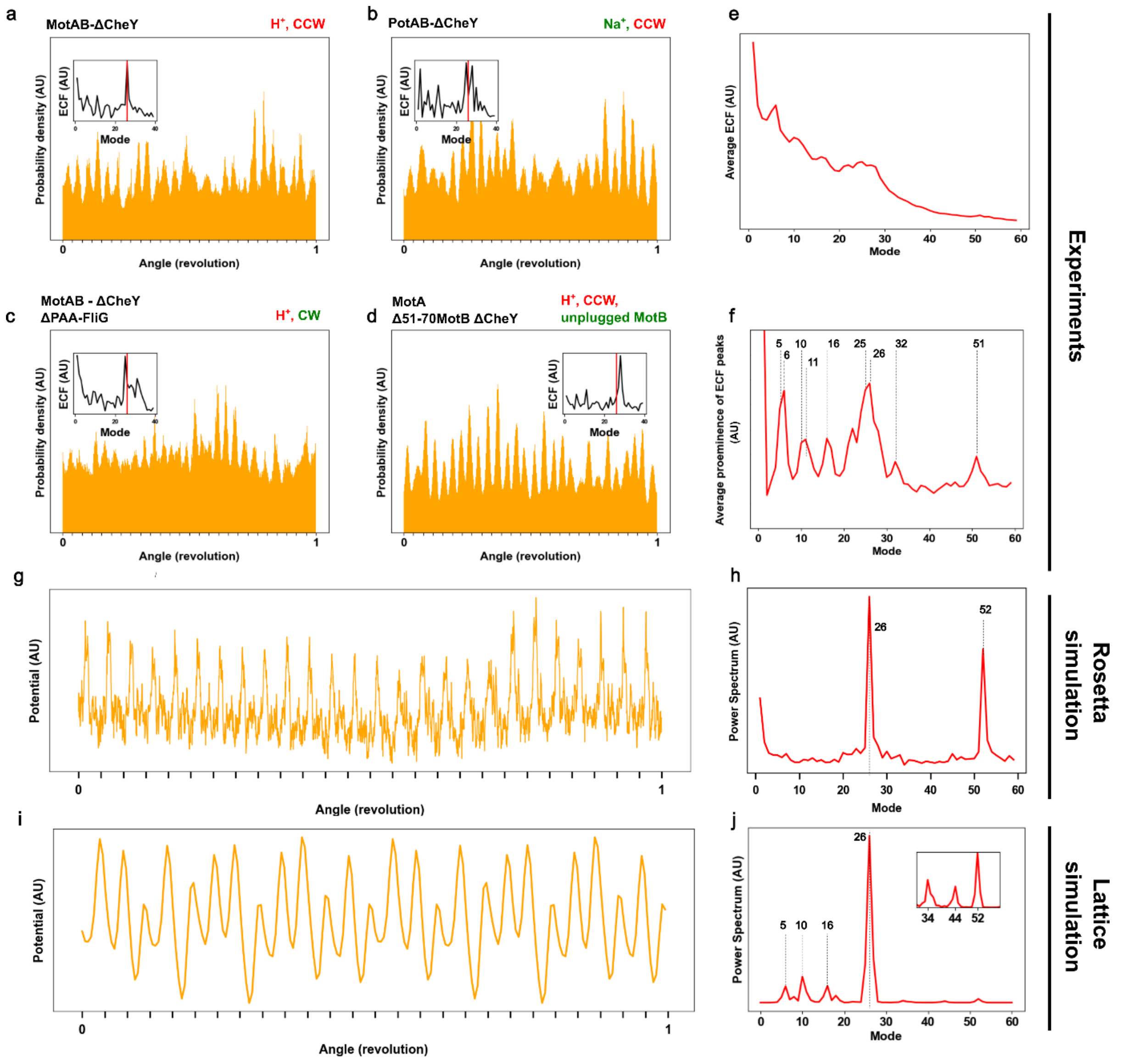

Figure S14: a-d. Histograms of the rotation angle ϕ averaged over 10s for four different motors of different strains. The variability of the histograms cannot be attributed to a particular mutant as within one strain they display similar variabilities. Insets: empirical characteristic function (ECF) showing the main periodicities in dwell angle. The red line shows the mode 26. CW: clockwise. CCW: counterclockwise. **e.** Average ECF over all non-overlapping 0.4 s windows (10^5 data points) over 18 motors from different strains. The variation of background in the ECF from file to file smoothens the peaks. **f.** Average prominence of each ECF mode over the same windows as in e. We define the prominence as the power of a particular mode divided by the minimum power over that mode and its four nearest neighbours. **g.** Energy landscape of the LP-ring / rod interface calculated via Rosetta using the cryo-EM structure from Salmonella (PDB 7CGO, see Methods for details). **h.** Amplitude of the Fourier transform of G. **i.** Energy landscape simulated using a simplified lattice-model of the cryo-EM structure (Supplementary Note S8). **j.** Amplitude of the Fourier transform of i.

Supplementary Note S8: Simplified lattice model of the Flagellar Bearing.

We describe a simple simulation that makes the following predictions.

1. Most of the observed ECF peaks in actively rotating motors (Fig. S14) arise from the symmetry mismatch between the 26-fold LP-ring and helical flagellar rod.
2. To reproduce the observed ECF peaks, the simulated interaction potential between each rod monomer and each repeating unit of the LP-ring must include features many times smaller than a single rod monomer, and all 26 LP-ring repeating units cannot be identical.
3. The observed variability arises by simulating variation in the "defects" represented by non-identical rod or LP-ring repeating units and/or by slight shifts in the alignment of rod and LP-ring.

Fig. S14f identifies periodicities in flagellar rotation of 5, 6, 10, 11, 16, 32 and 51 in addition to the dominant periodicity around 26-fold. 5, 6 and 11 match the 5-, 6- and 11-start helices that characterize the protein lattices that span the distal components of the bacterial flagellum - from the export apparatus at the base of the rod, all the way to the tip of the filament. In particular, the flagellar bearing consists of the cylindrical rod which is a polymer built upon this helical lattice, rotating inside the 26-fold LP-ring which makes tight contact with the rod around one particular circular narrowing of the inside face of the LP-ring[9] (Fig. S15).

We made a simple model of the interaction potential of the bearing to explore whether the symmetry mismatch between the rod and LP-ring might be responsible for the observed periodicities in rotation.

Model

The model places a rod monomer at each lattice site, approximately in the pattern of the Cryo-EM structure. Each rod monomer is represented (in cylindrical 2D co-ordinates representing the circumference and axis of the flagellar rod) by a small number of Gaussian potentials of interaction with each LP-ring repeating unit. Multiple Gaussians allow rod monomers to be asymmetric, and the inclusion of narrow gaussians permits "sharp", short-range, features to be modelled (Fig. S16).

Each LP-ring repeating unit contributes a 1-dimensional z-section of the rod monomer potential to the total bearing rotation potential (Fig. S17, top), at a z value corresponding to the axial position of the LP-ring (Fig. S16, purple line). This is motivated by the structure which shows a very narrow "collar" where the LP-ring is in atomic contact with the rod. The total bearing rotation potential (Fig. S17, middle) is the sum of all 26 individual LP-ring repeating unit contributions, each phase shifted by 1/26 of a rev. (The 26-fold LP-ring periodicity can be replaced by other values to model different LP-ring symmetries.)

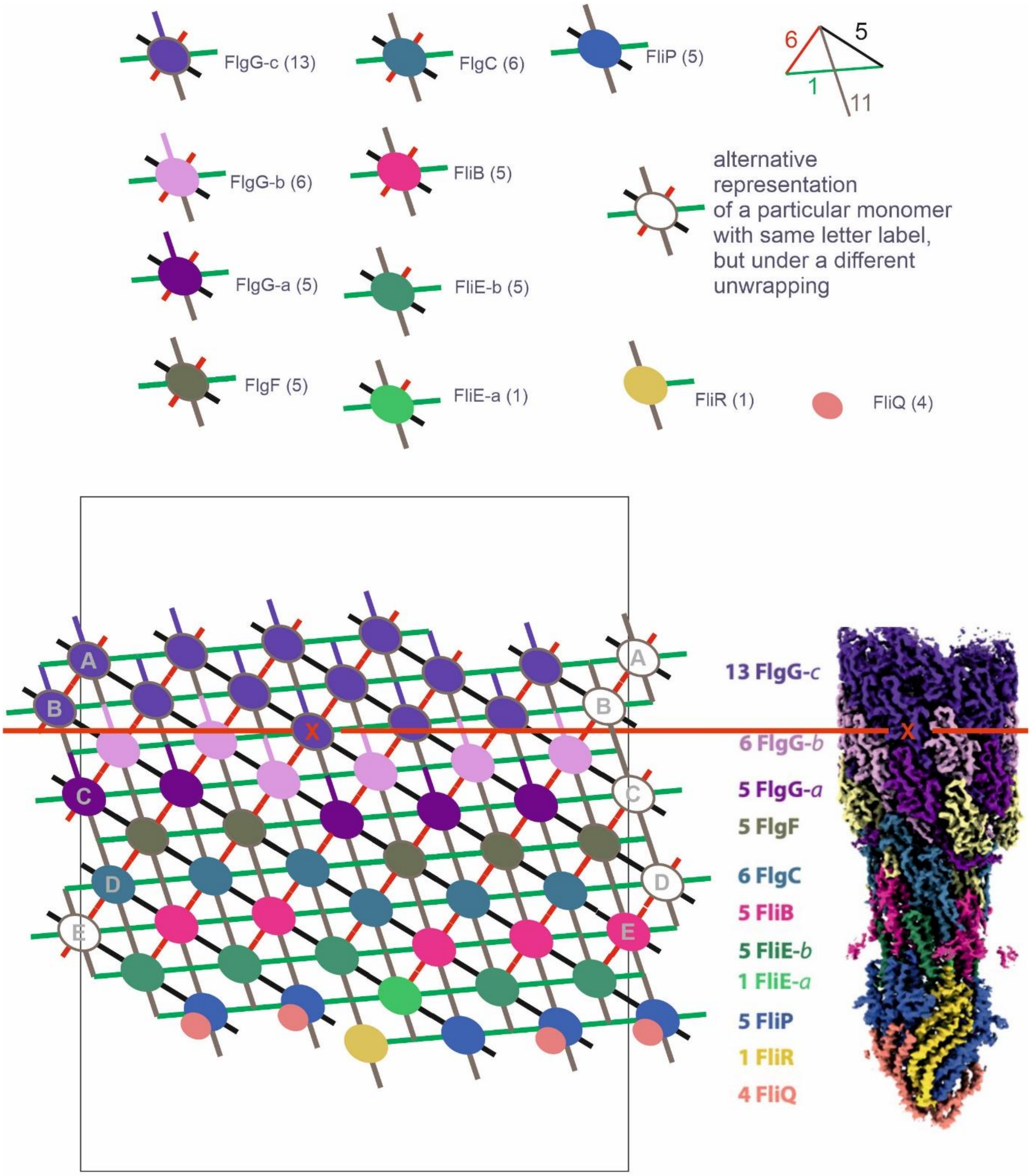

Figure S15: Representation of the monomer lattice of the flagellar rod, as seen in the structure of ([9]). The grey rectangle indicates the rod axis and monomers labelled A-E are duplicated either side of the seam along which the rod has been unrolled. Contacts between monomers on the 1, 5, 6 and 11 start helices are indicated by oblique coloured lines. The horizontal red line indicates the ring where the rod makes close contact with the encircling LP-ring in the flagellar bearing. The lowest FliG-c monomer is marked with a red "X" to illustrate the correspondence between the lattice and the rod structure.

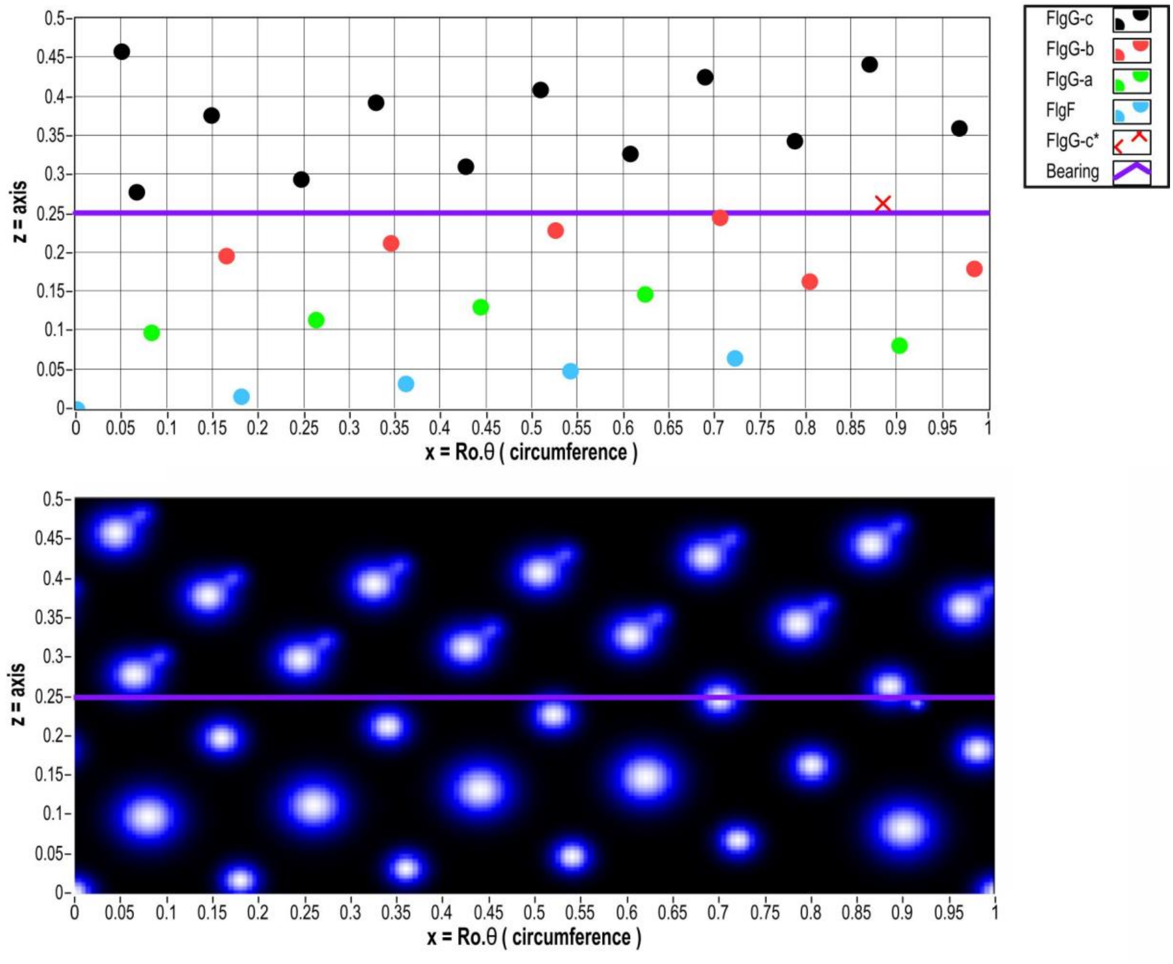

Figure S16: Model of the monomer lattice of the flagellar rod (top) and a typical model interaction potential between the rod and a single LP-ring monomer (bottom). The 4 different distal rod proteins are represented by lattice points of different colour and can have different sets of Gaussian potentials. A unique rod monomer (the first of type FlgG-c), potentially representing a defect or mis-folding, is indicated by a cross in the lattice (top). In this example, the unique monomer is at the axial position of the tight constriction in the LP-ring – the bearing interface (purple horizontal lines) – and has a sharp repulsive feature in its potential (bottom).

Results

Fig. S17 shows the monomer potential, the total bearing potential and its Fourier power spectrum, for the rod potential and LP-ring axial position of Fig. S16, in the case where all 26 LP-ring repeating units are identical. The peak periodicities at 6, 10, 16, and 26 are all observed in the experimental data, as is a peak near 52 which we attribute in the model to a harmonic of the dominant 26-fold periodicity. The experimental peaks observed at 5, 11 and 32 are weak in this case.

Fig. S18 shows the effects of defects in the rod lattice and LP-ring. Removing the sharpest feature of the rod potential, present in Fig. S16 due to a unique “defective” rod monomer, greatly reduces the 26-fold peak (top). Adding a defect in the LP-ring, modelled here by reducing 5-fold the strength of the interaction potential with one of the 26 LP-ring repeating units, enhances the 5, 6, 10 and 11-fold periodicities (middle). These effects are additive (bottom). This is to be expected: a period-1 defect in the LP-ring probes the helical lattice of the rod, which would otherwise be smoothed out by the non-commensurate 26-fold LP-ring symmetry. Similarly, sharp features on the rod lattice are period-1 probes of the 26-fold LP-ring – they do not average because each rod lattice site has a unique position in relation to the tight constricting ring in the bearing.

Fig. S19 Illustrates that any sharp features at the bearing interface will generate 26-fold periodicity in the total potential – here they are present in all rod monomers of the majority type (FlgG-b) at the constricting ring. The details of the potential are extremely sensitive to the exact positions and shapes of these sharp features. However, a small peak at 34 (close to the experimentally observed 32-fold peak) appears under most parameter sets of the model.

We were unable to identify parameters that substantially enhance the peak at 16, but many parameters sets remove it or shift it to 18.

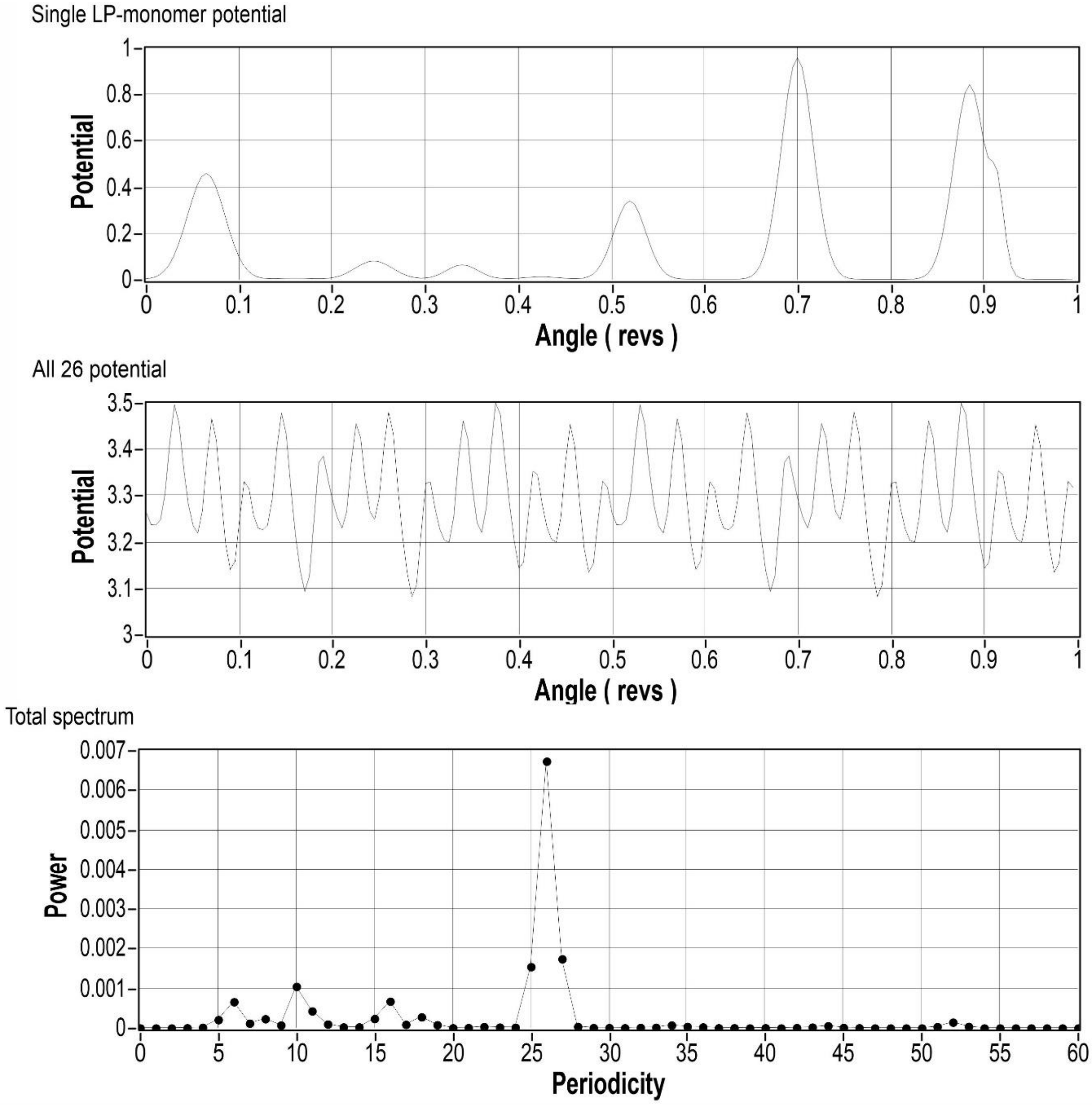

Figure S17: Single LP-unit potential (top), the total bearing potential (middle) and its Fourier power spectrum (bottom) for the rod potential and LP-ring axial position of Fig. S16, in the case where all 26 LP-ring repeating units are identical. Units of potential and power are arbitrary.

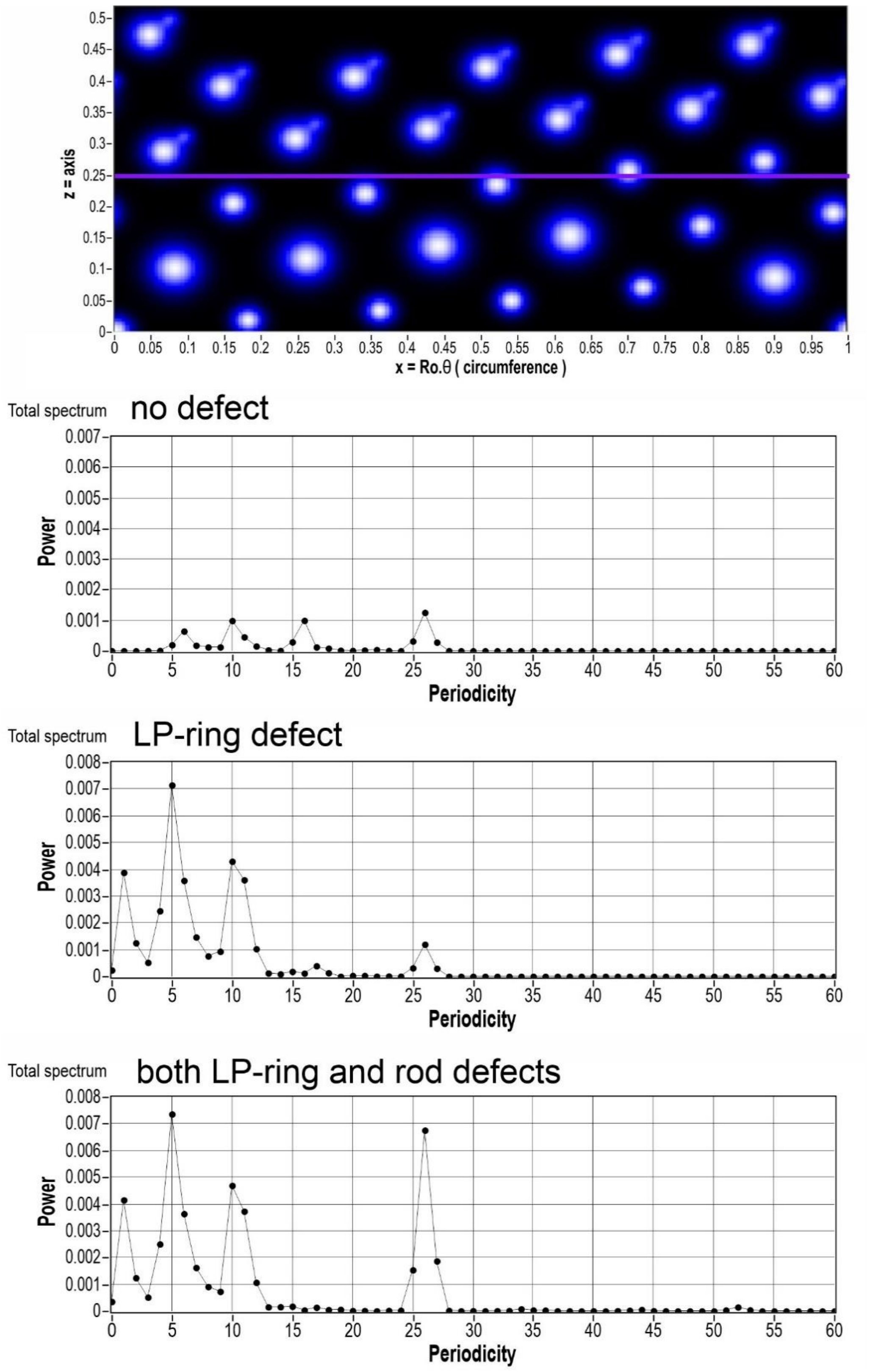

Figure S18: Removing the sharp feature in the rod potential (in Fig. S16, a defect in the unique rod monomer) greatly reduces the 26-fold periodicity (top). Adding a defect in the LP-ring enhances the 5- and 11-fold periodicities (middle, bottom).

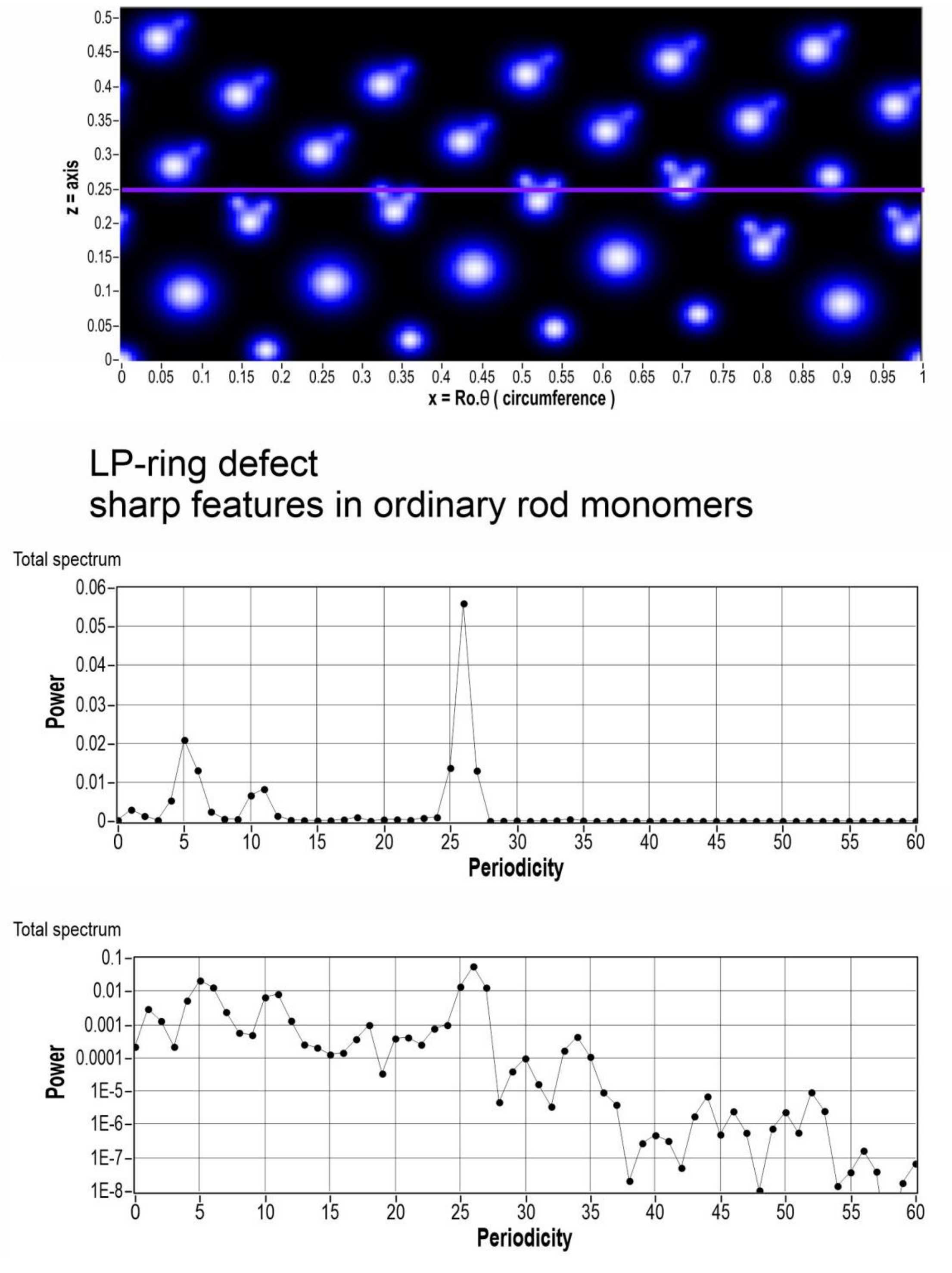

Figure S19: With a defect present in the LP-ring, sharp features in the rod potential present in all monomers (rather than only defective monomers as in Fig. S16 and 17), also enhance 26-fold periodicity (top). A small peak at 34-fold also emerges in such cases, visible on a logarithmic scale (bottom).

Supplementary Note S9: Symmetry breaking in the Rosetta simulation

The energy barriers computed by Rosetta (Figures 4b,c) show angular correlations, similar to the correlated variability in transition times observed in Figure 3b. We checked if Rosetta would converge to different results depending on the initial conditions, which would indicate a rough multi-dimensional system with many local stable states and could explain the slow diffusion that we observe within dwells. On the contrary, the Rosetta results yielded very low deviation over many runs, suggesting a consistent and well-defined 1D energy landscape, and thereby failing to provide any evidence for disorder.

An ideal Rosetta potential should be 26-fold symmetric: perfect 1/26 rev rotation of perfectly 26-fold symmetric LP-ring should yield an exactly equivalent starting point for Rosetta trajectories representing the potential at angles separated by 1/26 rev, and therefore the same energy for these angles, and therefore a 26-fold symmetric potential. But our simulated Rosetta potential breaks 26-fold symmetry, instead it better fits the experimental data: some barriers are bigger than others and barriers show angular correlations. Detailed examination of the Cryo-EM structure used as the starting point in the Rosetta simulation shows that the LP-ring simulated is NOT perfectly 26-fold symmetric. That these small deviations from the ideal 26-fold symmetry generate the large asymmetries seen in Figures 4b,c illustrates that bearing potentials generated from Rosetta trajectories are very sensitive to initial conditions. We speculate that this sensitivity arises at least in part from the atomically tight bearing interface, where even small side-chain movements could lead to relatively large changes in interaction energy.

Supplementary references

1. Park, K., Biswas, S., Kanel, S., Nepal, D. & Vaia, R. A. Engineering the Optical Properties of Gold Nanorods: Independent Tuning of Surface Plasmon Energy, Extinction Coefficient, and Scattering Cross Section. *J. Phys. Chem. C* **118**, 5918–5926 (2014).
2. Andrén, D. *et al.* Probing Photothermal Effects on Optically Trapped Gold Nanorods by Simultaneous Plasmon Spectroscopy and Brownian Dynamics Analysis. *ACS Nano* **11**, 10053–10061 (2017).
3. Mandadapu, K. K., Nirody, J. A., Berry, R. M. & Oster, G. Mechanics of torque generation in the bacterial flagellar motor. *Proc. Natl. Acad. Sci.* **112**, E4381–E4389 (2015).
4. Lee, A. & Cognet, L. Length measurement of single-walled carbon nanotubes from translational diffusion and intensity fluctuations. *J. Appl. Phys.* **128**, 224301 (2020).
5. Aragon, S. R. & Flamik, D. High Precision Transport Properties of Cylinders by the Boundary Element Method. *Macromolecules* **42**, 6290–6299 (2009).

6. Yuan, J. & Berg, H. C. Resurrection of the flagellar rotary motor near zero load. *Proc. Natl. Acad. Sci.* **105**, 1182–1185 (2008).
7. Nord, A. L., Sowa, Y., Steel, B. C., Lo, C.-J. & Berry, R. M. Speed of the bacterial flagellar motor near zero load depends on the number of stator units. *Proc. Natl. Acad. Sci.* **114**, 11603–11608 (2017).
8. Kramers, H. A. Brownian motion in a field of force and the diffusion model of chemical reactions. *Physica* **7**, 284–304 (1940).
9. Johnson, S. *et al.* Molecular structure of the intact bacterial flagellar basal body. *Nat. Microbiol.* **6**, 712–721 (2021).
