# Supplementary material for: Heterogeneity and multi-scale dynamics in the molecular bearing of the bacterial flagellum
Source: Nat Commun. 2026 Jun 12;17:7496. doi: 10.1038/s41467-026-74079-9 (PMC13408457; doi:10.1038/s41467-026-74079-9)
Supplement: Supplementary file 2 — Description of Additional Supplementary Files [file 41467_2026_74079_MOESM2_ESM.pdf]

Title: Supplementary Movie 1

Description: Rotation of a PotAB motor as captured by our experiment. The motor is initially rotating at ~100 Hz in 5 mM NaCl and then goes into diffusion motion a few minutes after removing the sodium. Top left: Raw polarization data as a function of time. Top right: representation on the unit sphere of the inferred orientation (θ,ϕ) of the gold nanorod. Bottom left: Inferred angle ϕ as a function of time. Bottom right: Representation of ϕ on a unit circle.

Title: Supplementary Movie S2 to S7

Description: Dynamic representation of all the data presented in this work. Top: Positions of the preferred angular positions of the motor in each window. Middle: Windowed histograms of the angle ϕ. Bottom: Enlarged view of the time trace of ϕ. The red region corresponds to the window used to perform the histograms. **S2**: PotAB – Motor1. **S3**: PotAB – Motor 2. **S4**: MotAB – Motor 1. **S5**: MotAB – Motor 2. S6**:** MotAB – Motor 3. **S7**: MotAB – Motor 4.
