# Supplementary material for: Heterogeneity and multi-scale dynamics in the molecular bearing of the bacterial flagellum
Source: Nat Commun. 2026 Jun 12;17:7496. doi: 10.1038/s41467-026-74079-9 (PMC13408457; doi:10.1038/s41467-026-74079-9)
Supplement: Supplementary file 10 — Reporting Summary [file 41467_2026_74079_MOESM10_ESM.pdf]

nature portfolio

Corresponding author(s): Richard M Berry

Last updated by author(s): Jun 11, 2025

Reporting Summary

Nature Portfolio wishes to improve the reproducibility of the work that we publish. This form provides structure for consistency and transparency in reporting. For further information on Nature Portfolio policies, see our Editorial Policies and the Editorial Policy Checklist.

Statistics

For all statistical analyses, confirm that the following items are present in the figure legend, table legend, main text, or Methods section.

n/a	Confirmed	
☐	☒	The exact sample size (*n*) for each experimental group/condition, given as a discrete number and unit of measurement
☐	☒	A statement on whether measurements were taken from distinct samples or whether the same sample was measured repeatedly
☐	☒	The statistical test(s) used AND whether they are one- or two-sided<br>*Only common tests should be described solely by name; describe more complex techniques in the Methods section.*
☒	☐	A description of all covariates tested
☐	☒	A description of any assumptions or corrections, such as tests of normality and adjustment for multiple comparisons
☐	☒	A full description of the statistical parameters including central tendency (e.g. means) or other basic estimates (e.g. regression coefficient) AND variation (e.g. standard deviation) or associated estimates of uncertainty (e.g. confidence intervals)
☐	☒	For null hypothesis testing, the test statistic (e.g. *F*, *t*, *r*) with confidence intervals, effect sizes, degrees of freedom and *P* value noted<br>*Give P values as exact values whenever suitable.*
☒	☐	For Bayesian analysis, information on the choice of priors and Markov chain Monte Carlo settings
☒	☐	For hierarchical and complex designs, identification of the appropriate level for tests and full reporting of outcomes
☒	☐	Estimates of effect sizes (e.g. Cohen's *d*, Pearson's *r*), indicating how they were calculated

Our web collection on statistics for biologists contains articles on many of the points above.

Software and code

Policy information about availability of computer code

Data collection	Custom Code was written in LabVIEW. It can be provided upon request.
Data analysis	Custom Code was written in Python. All the code used to process and interpret the data have been published in a Github repository linked to Zenodo https://doi.org/10.5281/zenodo.20094669.

For manuscripts utilizing custom algorithms or software that are central to the research but not yet described in published literature, software must be made available to editors and reviewers. We strongly encourage code deposition in a community repository (e.g. GitHub). See the Nature Portfolio guidelines for submitting code & software for further information.

Data

Policy information about availability of data

All manuscripts must include a data availability statement. This statement should provide the following information, where applicable:

- Accession codes, unique identifiers, or web links for publicly available datasets
- A description of any restrictions on data availability
- For clinical datasets or third party data, please ensure that the statement adheres to our policy

All raw and processed data generated in this study have been deposited in the Zenodo database under accession code https://zenodo.org/records/20088460 .

Research involving human participants, their data, or biological material

Policy information about studies with human participants or human data. See also policy information about sex, gender (identity/presentation), and sexual orientation and race, ethnicity and racism.

Reporting on sex and gender	N.A.
Reporting on race, ethnicity, or other socially relevant groupings	N.A.
Population characteristics	N.A.
Recruitment	N.A.
Ethics oversight	N.A.

Note that full information on the approval of the study protocol must also be provided in the manuscript.

Field-specific reporting

Please select the one below that is the best fit for your research. If you are not sure, read the appropriate sections before making your selection.

- [x] Life sciences
- [ ] Behavioural & social sciences
- [ ] Ecological, evolutionary & environmental sciences

For a reference copy of the document with all sections, see nature.com/documents/nr-reporting-summary-flat.pdf

Life sciences study design

All studies must disclose on these points even when the disclosure is negative.

Sample size	A grand total of 960,000,000 data points were sampled from 6 different flagellar bearings of 2 different bacterial strains. This is vastly more that is typical in the field of single-molecule biology, and requried the development of new statistical analysis tools.
Data exclusions	Data were excluded where gold nanorods were not rotating around an axis close to the optical axis, as this would compromise the primary variable measured, bearing angle
Replication	6 different flagellar bearings of 2 different bacterial strains were sampled. All were very obviously non-identitical, which is the one result of the paper. Yet all replicated the key features identified in the paper - thermally driven rotation and dynamic and static heterogeneity. Two bearings (> 300,000,000 data samples) would have been sufficient to demonstrate this.
Randomization	This is not relevant. 2 clones of bacteria were used, one of each E. coli strain. Members of each clone are nominally indentical and individual cells were selected for measurability of the bearing rotation angle. The paper discovered that despite this each individual flagellar bearing was different in its detailed rotation pattern.
Blinding	Blinding is not relevant. Individual bacterial are "blind" and nominally identical. The 2 strains are easily distinguishable and required different experimental protocols, requiring the experimenter to know their identity.

Reporting for specific materials, systems and methods

We require information from authors about some types of materials, experimental systems and methods used in many studies. Here, indicate whether each material, system or method listed is relevant to your study. If you are not sure if a list item applies to your research, read the appropriate section before selecting a response.

Materials & experimental systems

n/a	Involved in the study	
x		Antibodies
x		Eukaryotic cell lines
x		Palaeontology and archaeology
	x	Animals and other organisms
x		Clinical data
x		Dual use research of concern
x		Plants

Methods

n/a	Involved in the study	
x		ChIP-seq
x		Flow cytometry
x		MRI-based neuroimaging

Animals and other research organisms

Policy information about studies involving animals; ARRIVE guidelines recommended for reporting animal research, and Sex and Gender in Research

Laboratory animals	N.A.
Wild animals	N.A.
Reporting on sex	N.A.
Field-collected samples	N.A.
Ethics oversight	N.A.

Note that full information on the approval of the study protocol must also be provided in the manuscript.

Plants

Seed stocks	N.A.
Novel plant genotypes	N.A.
Authentication	N.A.
