# Supplementary material for: Heterogeneity and multi-scale dynamics in the molecular bearing of the bacterial flagellum
Source: Nat Commun. 2026 Jun 12;17:7496. doi: 10.1038/s41467-026-74079-9 (PMC13408457; doi:10.1038/s41467-026-74079-9)
Supplement: Supplementary file 11 — Transparent Peer Review file [file 41467_2026_74079_MOESM11_ESM.pdf]

nature portfolio

Peer Review File

Heterogeneity and multi-scale dynamics in the molecular bearing of the bacterial flagellum

Corresponding Author: Professor Richard Berry

This file contains all reviewer reports in order by version, followed by all author rebuttals in order by version.

Version 0:

Reviewer comments:

Reviewer #1

(Remarks to the Author)
I found this paper highly fascinating. The four-channel polarization measurement of scattered light from a gold nanorod firmly attached to the hook enabled the authors to analyze discrete 26-step transitions within a single revolution of the bacterial flagellar motor, with unprecedented temporal and angular resolution. These results, together with subsequent thermodynamic analysis, revealed heterogeneous energy landscapes of passively rotating mechanisms—specifically, the circularly aligned bearing and rod components. Their Rosetta-based simulation partially explains the heterogeneous energy landscape of the 26-fold barriers, although the reason for temporal changes remains an open question.
I would like to raise several questions and comments, both from the perspective of a general reader and regarding the technical aspects of the polarization measurements.

Q1.
The authors' analysis of stepwise rotations is based on the assumption that the origins of these steps lie solely within the bearing–rod mechanisms. However, since their improved polarization measurement has the potential to detect much smaller steps in addition to the 26 steps, it seems reasonable to consider possible contributions from other rotor–stator interactions, particularly in explaining the puzzling results in Fig. 3h,i. I understand that the torque-generating components are unlikely to be involved, as the comparison of passive rotations between motors with PotAB and those of mutants lacking torque-generating components showed no detectable differences in the presented data and analyses—suggesting that torque-generating components do not influence stepwise rotation. Nevertheless, I encourage the authors to comment on this point in the Discussion.

Q2.
It is clear that the atomic structures of the bearing components and those of the rod exhibit asymmetric profiles along their circular interaction faces. Yet both the in situ measurements and the analysis shown in Fig. 2b, as well as the Rosetta simulations, show no signs of asymmetric transitions between CW and CCW rotation. Is this absence due to technical limitations of the measurements or of the simulations? It is puzzling that structural asymmetry in the bearing–rod alignment produces symmetric transitions. I would appreciate the authors' clarification of this point in the Discussion.

Technical Questions and Comments on Methods
The following comments concern technical aspects of the polarization measurement. Using a microscopic system with a 45° dielectric mirror for polarization analysis is inherently challenging, but the authors' methods appear to include the necessary calibrations and corrections, particularly to compensate for distortions introduced by the mirror.

C1.
Large distortions occur along the 45°/135° polarization axes. The authors corrected these signals using a Berek compensator. After correction, what is the extinction factor for the 45°/135° polarizations, and how does it compare with extinction values at 0°/90°? This information would be very useful for researchers facing the same issue.

C2.
In Fig. 1e and Supplementary Video 1, the authors present movies showing changes in the azimuthal angle φ and polar angle θ during motor rotation. Fourkas's formula provides absolute orientation for φ, but not for θ, as the formula does not assign signs (i.e., hemispheres in the movie presentation) to θ. This angular degeneracy in determining θ should be

explicitly noted in Fig. 1 and in the Supplementary Information.

C3.
For analysis, the authors state that they selected datasets in which the rotation axis of the nanorod was nearly normal to the focal plane of the microscope. This is a reasonable filtering step to avoid data from rods with tilted rotation axes. The selection was based on the amplitude of θ, which increases as the tilt grows larger. I suggest that the authors present time-dependent plots of θ during gold nanorod rotation and provide quantitative criteria for dataset selection based on θ. Moreover, because of the ambiguity in the sign of θ, selection based solely on θ may not fully eliminate datasets with tilt away from the optical axis.

C4.
The authors state in the Introduction that their system achieves ~0.5° resolution on a timescale as short as 10 μs, but they do not provide supporting evidence in the text. I infer that this estimate comes from Extended Data Fig. 1b, where the SD of φ depends on the scattered light intensity detected by the APD. However, I could not find the APD voltage used for measurements at the highest time resolution (10 μs). The text should include a reference or justification for this estimation.

C5.
Drilling a perfectly circular hole in a 45° mirror with rotational symmetry about the optical axis is technically challenging. Any irregularity in the hole's shape could compromise uniform sensitivity in determining absolute polarization orientation. To assess such effects, isotropic emitters such as small fluorescent molecules (with negligible rotational correlation times) could be used under polarization-neutral excitation. Although the authors provide simulation results in Extended Data Fig. 2i–l, these may not be sufficient to fully evaluate the hole's effect. I recommend including a clear explanation of why any irregularity in the hole shape does not impair uniform sensitivity across the measurement range.

C6.
Please provide the numerical aperture values (or iris diameters) used in the simulations at the back focal plane in Extended Data Fig. 2. This information would help readers assess the relative size of the hole compared with the aperture of the objective lens.

Reviewer #2

(Remarks to the Author)
The paper by Rieu et al. uses polarization readout of gold nanorods attached to the hook to track flagellar angular motion with ~0.5° angular resolution at 250 kHz, enabling long, continuous single-motor records. Using statorless (ΔMotAB) and Na+-depleted PotAB conditions, the authors resolve ~26 angular states, non-Poisson transition times, anomalous diffusion within dwells, and a slow drift of preferred angles. The authors connect these behaviors to the Rosetta-computed energy landscape of the LP-ring/rod interface. This is an ambitious study, the instrumentation and dataset resolution are quite impressive. However, some interpretational leaps and analysis choices need firmer grounding to firmly attribute the observed dynamics to the bearing.

Major concerns:

- The manuscript assigns the observed angular states to the LP-ring/rod interface but offers no direct evidence for this claim. Other sources (hook mechanics, probe coupling, cell-body motion, or residual stator activity) could, in principle, contribute to the observed signatures. Convincing attribution can be made through orthogonal perturbations that specifically target LP-ring interactions (e.g., targeted FlgH/I variants), while holding other components constant. As presented, the 26-fold symmetry is suggestive but not definitive.

- Another approach to selectively target the LP-ring could involve media that specifically influence periplasmic pH and/or ion strength, which would be expected to influence the Rod-LP-ring interface while leaving the cytoplasmic elements untouched.

- Reproducibility and sample size are a concern. Only one out of six bearings showed stable state positions (line 119). Beyond that, data from only five additional motors are reported. With such limited sampling, it is hard to know whether the behavior reported here can be generalized.

Other concerns:

Approach, Analysis and Reporting:
- The authors should comment on the fact that their experiments are conducted in E. coli while the Rosetta analysis is based on Salmonella structures.

- What is the reason for choosing to discard all steps less than specifically 10 degrees (line 411)? Does changing this threshold change the results of analysis?

- It is unclear as to why the transition times for the five bearings where global states could not be consistently defined were taken to be twice the measured lifetimes (line 141).

- Can the authors report the size and heterogeneity of the gold nanorods and whether this is expected to influence the outcome of the study?

- The authors note that they excluded any cells for which the axis or rotation was close to parallel to the optical axis. They should report how many cells were excluded and whether included cells differ from the population in terms of the probe load/attachment geometry. Could the observed dynamics and heterogeneity correlate with these parameters?

- Rod drag coefficient is estimated from rods loosely attached to the surface. The rotational diffusion of such rods is expected to be affected by boundary interactions. I would expect that rods farther from the surface, such as those attached to the hook, would have a lower drag coefficient. Thus, the barrier height could be much smaller than what is estimated and reported in the summary.

- Orientation reconstruction is entirely based on Fourkas' formula and has not been independently tested or calibrated. Ideally, calibration should be performed by running the procedure with a known rotating scatterer (e.g., an immobilized rod mounted on a piezo rotary stage).

Interpretation:
- If the bearing does have structural plasticity (line 212), how do we know that the energetics described here are the same as when a motor is driven by multiple stators?

- How do the authors know that the motors are diffusing freely and that any stators are no longer engaged after Na+ is removed? After all, their data in Fig. 1 show a clear signature of transient activation of a stator. It is conceivable a stator that remained engaged but only generated rotation when trace sodium was available.

- In delta motAB M1, M3, M4 (Extended data figs 5 and 6), why do the traces show prolonged, mostly unidirectional rotation lasting ~10-100 seconds? This would be highly unexpected under passive diffusion. The authors should quantify the directional bias of rotational diffusion to test whether the motion is symmetric or preferentially CCW/CW.

- Lines 199-204: While a stator can generate 200 pN nm, the torque per stator unit changes with external load, therefore the torque generated by a stator and the average energy barrier is not a simple comparison. Presumably, a motor would not be experiencing zero load conditions very often, and would most commonly experience the load of a flagellar bundle. Under these conditions, one unit would not need to overcome the barriers, we expect more than one stator unit to be recruited to the motor. Additionally, stator units generate different amounts of torque depending on direction of rotation, and thus would have different interactions with the bearing's energy barrier. The statement that stator unit torque coevolved with the energy barrier of the bearing lacks evidence and requires more nuanced discussion on the function of stators.

- In the abstract the authors note: "These findings raise the question of whether heterogeneous dynamics are a universal feature of large protein machines." It is unclear what supports this claim. I did not see a discussion in the main text linking their observations to other molecular machines, so the basis for suggesting universality should either be explained explicitly or the claim toned down.

Presentation:
- The figures feel overly dense, with many panels and insets packed together (e.g., Fig. 2). This makes it hard to follow the data in parallel with the text. Splitting some figures into multiple, more focused ones would improve readability and help highlight key results.

- The main text contains extensive methodological detail (e.g., lines 76–80) that interrupts the narrative flow. Moving some of this material to the Methods section would make the paper more accessible.

- Relatedly, the writing overall is a bit dense and would benefit from editing to make the paper more approachable to a general audience. As written, the paper will be easiest to follow for the narrow intersection of scientists interested in flagellar motors and stochastic processes. Smoothing the prose and adding more explanation in some of the more technical passages would broaden the paper's reach.

- A few typographical errors were noted. When writing species names (e.g. L20, L558), the genus name is capitalized but the specific epithet should remain lowercase. In L560 "unwrapped angle f…" likely is meant to be the Greek letter phi.

Reviewer #3

(Remarks to the Author)
The manuscript by Rieu and coworkers reports on a new method to measure the rotation of the bacterial flagellar motor with unprecedented spatiotemporal resolution for angular displacements. The authors developed a high-bandwidth - spanning about seven orders of magnitude in time - polarization microscope by using gold nanorods as probes. The gold nanorods enable a fast response time and long measurement durations. Surprisingly, they found rich dynamics and heterogeneity of both active and passive motors that they convincingly attribute to subtle asymmetries in the almost rotationally symmetric structure of the motor and the different protein lattices in the flagellum. Small fluctuations and changes in structure over time may affect the overall energy landscape leading to the complex dynamics that the authors have measured. Future

experiments will have to show whether other large molecular machines are subjected to similar effects and whether they serve a biological function. The manuscript is very well written and the novel methodology can be applied to wide range of systems. I strongly recommend publication after my minor points below are addressed.

1. Greek symbols for angles in the figure caption of Fig. 1 need to be fixed.
2. Fig. 1d: In my opinion, it would be better to only show 10 ms to see more details of the 4 channels.
3. Extended Data Fig. 1: in addition to the PSD and integrated-noise plots, a plot of the angular Allan deviation as a function of averaging time would be very informative.
4. Extended Data Fig. 2: less but larger labels would be helpful.
5. Extended Data Fig. 3: please indicate the expected rotational drag coefficient based on the AuNR dimensions in the figure. Was the proximity to the surface considered? It may increase the drag coefficient somewhat (maybe 10-20 percent, see e.g. Brenner H Appl. Sci. Res. 13:81–120 (1964)).
6. Fig. 1b: Why is there a PBS underneath the QWP? Please introduce all abbreviations in the caption (BP is missing). A more detailed schematic and list of components in particular of the three levels described in Supp. Method S1 would be helpful for the supplement (including some pictures of this implementation).
7. Heating effects: the authors may consider to measure the drag coefficient of surface attached AuNR as a function of excitation power for a single rod. If there is no significant change that would be a good control for heating effects.
8. l. 104: there is no upper left panel.
9. Methods: l. 325/328 fix the missing Ohm symbol
10. Supplementary Note 6: The equation for Kramer's time: I presume the authors used the equation as stated - if so they should replace the proportional sign with an equal sign. Please also add the missing Boltzmann constants in the equation.
11. Fig. 2a: Does the inset correspond to (3)? In d and i and Fig. 3g, the Delta is missing for the DeltaMOTAB.
12. Extended Data Figure 9: Please increase the figure size. The labels and equations are difficult to read and pixelated.
13. Extended Data Figure 10: labels for c and e are exchanged.
14. Supplementary Note 7: The details of the model remain rather vague and the code is not provided. As stated it is not reproducible.

Version 1:

Reviewer comments:

Reviewer #1

(Remarks to the Author)
#1.1 (Q1)
We agree that torque-generating components are unlikely to be involved. We have expanded our discussion of the possible contributors to the extremely complex behaviour that we observed, including to address specifically the results of Fig 3h,i (now 6 b,c)– the changes over time in the number and location of dwells. (lines 192-7, 240)

(Comment from the reviewer 1)
I agree with the authors’ response and appreciate the additional discussion on contributions from components other than the bearing.

#1.2 (Q2)
In the absence of a thermodynamic driving force there can be no overall asymmetry between CW and CCW transition rates, regardless of the possibility of asymmetric “sawtooth” transition barriers. That would violate microscopic reversibility. In Fig 2b we chose to pair transition times out of a given state in either direction to illustrate the observation that there is generally less asymmetry within such a pair than between pairs. In other words, if for example a CW transition out of a particular state is slow the CCW transition out of that state is also likely to be slow. This indicates that there is more variation between energy minima than maxima: minima affect rates out of a state in both directions equally, maxima pair CCW transitions from one state with CW from the next state. However there are exceptions - e.g. the CW and CCW transition times out of state 4 are ~10-fold different. We might speculate, with little support, that a particularly stable interaction might create a deep minimum (a trap), while the conformational flexibility of the system precludes large variations in barrier height. But it is also possible that these are features peculiar to this particular bearing. We have added a discussion of these questions to the discussion (lines 210-214).

(Comment from the reviewer 1)
I am satisfied with the clarification provided above, as well as the additional discussion included in the revised manuscript.

#1.3 (C1)
We have now measured the extinction ratios for 45°/135°polarizations, and compared them to those at 0°/90°. A report of this has been added to Supplementary Method S1, Alignment of the emission path, C. Extinction ratios were ~200:1 for 0° and 90° polarizations, ~220:1 for 135° and ~90:1 for 45°.

(Comment from the reviewer 1)
The newly added information will be very helpful for readers who wish to adopt or reproduce this experimental approach.

#1.4 (C2)
There are TWO degeneracies, one in each of φ and θ, as described in the section "Orientation reconstitution" along with our treatment of them. We have added clarification of the treatment of the θ degeneracy to the legend of Fig. 1 (line 579) and to Supplementary Method S2.

(Comment from the reviewer 1)
It is indeed correct that the degeneracy in recovering the orientation of the nanorod applies to both θ and φ. I appreciate the clarification added to the legend of Fig. 1 and to Supplementary Method S2.

#1.5. (C3)
As explained in Methods, we did NOT select based on the amplitude of θ, but rather on the similarity of the two loops in the anisotropy plot which we now illustrate (Supplementary Method S2) to be a double covered mapping of the theta/phi space. This selects for rotation axes close to the optical axis, but the angle between the nanorod long axis and the rotation axis, which is equal to θ in our chosen nanorods where the rotation axis coincides with the optical axis, remains variable and is NOT selected for. In practice we typically chose nanorods with θ in the range 40-60° (now shown in Extended Data Figs 7-9), avoiding the region around θ = π/2 (90°) where the degeneracy might be relevant. We note however that in this region the recovery of φ is particularly insensitive to the θ degeneracy, which would therefore have minimal effect on our measurements of motor angle. We have added time-dependent plots of θ for all bearings to Extended Data Figures 7-9. We also discovered and used an improved form of the analytical expression for θ, and we changed Supplementary Method S2 to describe this new procedure.

(Comment from the reviewer 1)
I have read the authors' explanation and the corresponding revisions in the manuscript, and I find their arguments convincing. It is also good to see that the authors have identified an improved analytical expression for θ.

#1.6 (C4)
We have added a supporting reference to Extended Data Fig. 1 and the APD voltages used for bearing measurements to the Methods section (line 386-9).

(Comment from the reviewer 1)
Providing this technical information is helpful and appropriate.

#1.7 (C5)
We have clarified in the legend to Extended Data Fig. 2 that the simulation used the actual measured hole, including irregularities. We believe that our simulations quantify adequately the scale of the effect of the hole upon the recovered angles.

(Comment from the reviewer 1)
I understand the authors' explanation that the hole size and its irregular shape do not significantly affect the measurements.

#1.8 (C6)
We have added this information to the legend to Extended Data Fig. 2.

(Comment from the reviewer 1)
The additional information will be useful for readers interested in the microscopic measurement system.

Reviewer #2

(Remarks to the Author)
In response to the comments from the three reviewers, the authors have made substantial and meaningful revisions to the manuscript. These changes adequately address my main concerns. I have a few remaining comments below; however, I note that while these are relatively minor, and I do not view additional revisions as necessary for publication.

In their response to #2.7 the authors say "It is not possible that this selection of well-oriented rods and rotation axes could have any effect on the observed dynamics and heterogeneity."

I don't really understand why this is the case but maybe I am missing something.

In their response to #2.14, the authors write "Because this is the first ever single-molecule investigation of a large protein complex …, the current evidence is that ALL such complexes that have been observed show them. Therefore it is reasonable to hypothesize that they are universal, pending further evidence."

This is a personal view, but hypothesizing universality from observations of a single system seems like a stretch. The authors are certainly within their rights to raise this as a question, but I am not convinced it warrants a prominent place in the abstract.

Reviewer #3

(Remarks to the Author)
The reviewers have addressed all of my concerns. I fully support the manuscript's publication.

Open Access This Peer Review File is licensed under a Creative Commons Attribution 4.0 International License, which permits use, sharing, adaptation, distribution and reproduction in any medium or format, as long as you give appropriate credit to the original author(s) and the source, provide a link to the Creative Commons license, and indicate if changes were made.
In cases where reviewers are anonymous, credit should be given to 'Anonymous Referee' and the source.
The images or other third party material in this Peer Review File are included in the article’s Creative Commons license, unless indicated otherwise in a credit line to the material. If material is not included in the article’s Creative Commons license and your intended use is not permitted by statutory regulation or exceeds the permitted use, you will need to obtain permission directly from the copyright holder.
To view a copy of this license, visit https://creativecommons.org/licenses/by/4.0/

We thank the reviewers for their detailed reading of the manuscript and their generally very positive comments. Point-by-point responses to the reviewers' comments are listed below. All changes in the revised manuscript are highlighted in red and linked by "comments" to the corresponding reviewers' comments, and their locations are included in the list below.

REVIEWER COMMENTS

Reviewer #1 (Remarks to the Author):

> ***I found this paper highly fascinating.*** *The four-channel polarization measurement of scattered light from a gold nanorod firmly attached to the hook enabled the authors to analyze discrete 26-step transitions within a single revolution of the bacterial flagellar motor, with unprecedented temporal and angular resolution. These results, together with subsequent thermodynamic analysis, revealed heterogeneous energy landscapes of passively rotating mechanisms—specifically, the circularly aligned bearing and rod components. Their Rosetta-based simulation partially explains the heterogeneous energy landscape of the 26-fold barriers, although the reason for temporal changes remains an open question.*
> *I would like to raise several questions and comments, both from the perspective of a general reader and regarding the technical aspects of the polarization measurements.*

Detailed responses to comments below.

> *#1.1 (Q1)*
>
> *The authors' analysis of stepwise rotations is based on the assumption that the origins of these steps lie solely within the bearing–rod mechanisms. However, since their improved polarization measurement has the potential to detect much smaller steps in addition to the 26 steps, it seems reasonable to consider possible contributions from other rotor–stator interactions, particularly in explaining the puzzling results in Fig. 3h,I . I understand that the torque-generating components are unlikely to be involved, as the comparison of passive rotations between motors with PotAB and those of mutants lacking torque-generating components showed no detectable differences in the presented data and analyses—suggesting that torque-generating components do not influence stepwise rotation. Nevertheless, I encourage the authors to comment on this point in the Discussion.*

We agree that torque-generating components are unlikely to be involved. We have expanded our discussion of the possible contributors to the extremely complex behaviour that we observed, including to address specifically the results of Fig 3h,i (now 6 b,c)– the changes over time in the number and location of dwells. (lines 192-7, 240)

> *#1.2 (Q2)*
>
> *It is clear that the atomic structures of the bearing components and those of the rod exhibit asymmetric profiles along their circular interaction faces. Yet both the in situ measurements and the analysis shown in Fig. 2b, as well as the Rosetta simulations, show no signs of asymmetric transitions between CW and CCW rotation. Is this absence due to technical limitations of the measurements or of the simulations? It is puzzling that structural asymmetry in the bearing–rod alignment produces symmetric transitions. I would appreciate the authors' clarification of this point in the Discussion.*

In the absence of a thermodynamic driving force there can be no overall asymmetry between CW and CCW transition rates, regardless of the possibility of asymmetric “sawtooth” transition barriers. That would violate microscopic reversibility. In Fig 2b we chose to pair transition times out of a given state in either direction to illustrate the observation that there is generally less asymmetry within such a pair than between pairs. In other words, if for example a CW transition out of a particular state is slow the CCW transition out of that state is also likely to be slow. This indicates that there is more variation between energy minima than maxima: minima affect rates out of a state in both directions equally, maxima pair CCW transitions from one state with CW from the next state. However there are exceptions - e.g. the CW and CCW transition times out of state 4 are ~10-fold different. We might speculate, with little support, that a particularly stable interaction might create a deep minimum (a trap), while the conformational flexibility of the system precludes large variations in barrier height. But it is also possible that these are features peculiar to this particular bearing. We have added a discussion of these questions to the discussion (lines 210-214).

Technical Questions and Comments on Methods

The following comments concern technical aspects of the polarization measurement. Using a microscopic system with a 45° dielectric mirror for polarization analysis is inherently challenging, but the authors’ methods appear to include the necessary calibrations and corrections, particularly to compensate for distortions introduced by the mirror.

#1.3 (C1)

Large distortions occur along the 45°/135° polarization axes. The authors corrected these signals using a Berek compensator. After correction, what is the extinction factor for the 45°/135° polarizations, and how does it compare with extinction values at 0°/90°? This information would be very useful for researchers facing the same issue.

We have now measured the extinction ratios for 45°/135° polarizations, and compared them to those at 0°/90°. A report of this has been added to Supplementary Method S1, Alignment of the emission path, C. Extinction ratios were ~200:1 for 0° and 90° polarizations, ~220:1 for 135° and ~90:1 for 45°

#1.4 (C2)

In Fig. 1e and Supplementary Video 1, the authors present movies showing changes in the azimuthal angle φ and polar angle ϑ during motor rotation. Fourkas’s formula provides absolute orientation for φ, but not for ϑ, as the formula does not assign signs (i.e., hemispheres in the movie presentation) to ϑ. This angular degeneracy in determining ϑ should be explicitly noted in Fig. 1 and in the Supplementary Information.

There are TWO degeneracies, one in each of ϕ and θ, as described in the section “Orientation reconstitution” along with our treatment of them. We have added clarification of the treatment of the θ degeneracy to the legend of Fig. 1 (line 579) and to Supplementary Method S2.

We also corrected a typographical error in the description of these degeneracies (line 380).

#1.5. (C3)

For analysis, the authors state that they selected datasets in which the rotation axis of the nanorod was nearly normal to the focal plane of the microscope. This is a reasonable filtering

step to avoid data from rods with tilted rotation axes. The selection was based on the amplitude of ϑ, which increases as the tilt grows larger. I suggest that the authors present time-dependent plots of ϑ during gold nanorod rotation and provide quantitative criteria for dataset selection based on ϑ. Moreover, because of the ambiguity in the sign of ϑ, selection based solely on ϑ may not fully eliminate datasets with tilt away from the optical axis.

As explained in Methods, we did NOT select based on the amplitude of θ, but rather on the similarity of the two loops in the anisotropy plot which we now illustrate (Supplementary Method S2) to be a double covered mapping of the theta/phi space. This selects for rotation axes close to the optical axis, but the angle between the nanorod long axis and the rotation axis, which is equal to θ in our chosen nanorods where the rotation axis coincides with the optical axis, remains variable and is NOT selected for. In practice we typically chose nanorods with θ in the range 40-60° (now shown in Extended Data Figs 7-9), avoiding the region around θ = π/2 (90°) where the degeneracy might be relevant. We note however that in this region the recovery of ϕ is particularly insensitive to the θ degeneracy, which would therefore have minimal effect on our measurements of motor angle.

We have added time-dependent plots of θ for all bearings to Extended Data Figures 7-9. We also discovered and used an improved form of the analytical expression for θ, and we changed Supplementary Method S2 to describe this new procedure.

#1.6 (C4)
The authors state in the Introduction that their system achieves ~0.5° resolution on a timescale as short as 10 μs, but they do not provide supporting evidence in the text. I infer that this estimate comes from Extended Data Fig. 1b, where the SD of φ depends on the scattered light intensity detected by the APD. However, I could not find the APD voltage used for measurements at the highest time resolution (10 μs). The text should include a reference or justification for this estimation.

We have added a supporting reference to Extended Data Fig. 1 and the APD voltages used for bearing measurements to the Methods section (line 386-9).

#1.7 (C5)
Drilling a perfectly circular hole in a 45° mirror with rotational symmetry about the optical axis is technically challenging. Any irregularity in the hole's shape could compromise uniform sensitivity in determining absolute polarization orientation. To assess such effects, isotropic emitters such as small fluorescent molecules (with negligible rotational correlation times) could be used under polarization-neutral excitation. Although the authors provide simulation results in Extended Data Fig. 2i–l, these may not be sufficient to fully evaluate the hole's effect. I recommend including a clear explanation of why any irregularity in the hole shape does not impair uniform sensitivity across the measurement range.

We have clarified in the legend to Extended Data Fig. 2 that the simulation used the actual measured hole, including irregularities. We believe that our simulations quantify adequately the scale of the effect of the hole upon the recovered angles.

We gave considerable thought to possible ways to calibrate the angle recovery, but as stated in Supplementary Note 2 we could not originally discover a satisfactory method to control accurately the angle of a nanorod while measuring it. Nonetheless we have added an attempted calibration of the ϕ angle recovery, with the hole, following comment #2.9. We controlled nanorod angle by rotating the entire sample by hand, with a nanorod immobilized non-specifically to the coverglass. Despite the expected and observed limitations of this method - in particular imperfect re-centering of the nanorod after each rotation and anisotropies in the sample chamber – we found a very close agreement between the recovered angle and the rotation angle of the stage, confirming our calculations that effects of the hole upon recovery of ϕ are indeed negligible in the range of angles that we measured. This calibration experiment is described in Extended Data Figure 3 and Supplementary Note S3.

> *#1.8 (C6)*
>
> *Please provide the numerical aperture values (or iris diameters) used in the simulations at the back focal plane in Extended Data Fig. 2. This information would help readers assess the relative size of the hole compared with the aperture of the objective lens.*

We have added this information to the legend to Extended Data Fig. 2.

<u>Reviewer #2 (Remarks to the Author):</u>

> *The paper by Rieu et al. uses polarization readout of gold nanorods attached to the hook to track flagellar angular motion with ~0.5° angular resolution at 250 kHz, enabling long, continuous single-motor records. Using statorless (ΔMotAB) and Na+-depleted PotAB conditions, the authors resolve ~26 angular states, non-Poisson transition times, anomalous diffusion within dwells, and a slow drift of preferred angles. The authors connect these behaviors to the Rosetta-computed energy landscape of the LP-ring/rod interface. This is an ambitious study, the instrumentation and dataset resolution are quite impressive. However, some interpretational leaps and analysis choices need firmer grounding to firmly attribute the observed dynamics to the bearing.*
>
> ***Major concerns:***
>
> *#2.1*
>
> *- The manuscript assigns the observed angular states to the LP-ring/rod interface but offers no direct evidence for this claim. Other sources (hook mechanics, probe coupling, cell-body motion, or residual stator activity) could, in principle, contribute to the observed signatures. Convincing attribution can be made through orthogonal perturbations that specifically target LP-ring interactions (e.g., targeted FlgH/I variants), while holding other components constant. As presented, the 26-fold symmetry is suggestive but not definitive.*
>
> *- Another approach to selectively target the LP-ring could involve media that specifically influence periplasmic pH and/or ion strength, which would be expected to influence the Rod-LP-ring interface while leaving the cytoplasmic elements untouched.*

We have expanded our discussion of other possible contributions to the observed behaviours, beyond the flagellar bearing - summarised in the response to comment #1.1 from reviewer 1, above. As discussed in the manuscript, we believe that the 26-fold symmetry and the lack of torque-

generating components are clear indications that the bearing is the dominant contributor to the observed dynamics.

The reviewer's suggestions for genetic and biochemical investigations that would attempt to shed light on which structures and biochemical conditions may affect the observed dynamics are valuable suggestions for future work, but well beyond the scope of this already very large manuscript.

#2.2

- Reproducibility and sample size are a concern. Only one out of six bearings showed stable state positions (line 119). Beyond that, data from only five additional motors are reported. With such limited sampling, it is hard to know whether the behavior reported here can be generalized.

While we present data from only 6 bearings, these data consist of more than an hour of recording at 250 kHz, a total of around 1 billion measured bearing angles. This is vastly more than other comparable single-molecule experiments. Our extensive and detailed analysis only begins to scratch the surface of this unprecedented dataset. This manuscript reports the new method, new data analysis tools that were required to analyse its results, and its first key finding which is that all flagellar bearings observed show complex and variable dynamics. We believe that very little would be gained by adding further bearings to the dataset. It is inconceivable that any of them would behave identically to any other, which is the only result that would alter the conclusions of this manuscript. Investigations of mutated bearings, bearings of different species, and bearings under different biochemical conditions are future work.

Other concerns:

#2.3

Approach, Analysis and Reporting:
- The authors should comment on the fact that their experiments are conducted in E. coli while the Rosetta analysis is based on Salmonella structures.

Done. (line 151)

#2.4

What is the reason for choosing to discard all steps less than specifically 10 degrees (line 411)? Does changing this threshold change the results of analysis?

We have added an explanation (line 429) that this is an arbitrary threshold significantly but not too far below the dominant 1/26 rev transitions (13.6 degrees on average) that we are aiming to quantify.

#2.5

- It is unclear as to why the transition times for the five bearings where global states could not be consistently defined were taken to be twice the measured lifetimes (line 141).

This is explained in the immediately preceding sentences:

“If the probabilities of moving to state (i+1) and (i-1) were equal, the average lifetime would be half the average transition time. This relationship was confirmed for PotAB Motor 1…”

#2.6

- Can the authors report the size and heterogeneity of the gold nanorods and whether this is expected to influence the outcome of the study?

We have added the nanorod dimensions to the Methods Section (line 259). EM revealed some variation in size and shape of nanorods, which may add to variability in rod orientation and location relative to the hook in causing drag coefficients to vary nearly ~10-fold between different individual nanorods (see Supplementary Note S6 and Extended Data Figure 5). However, the measured drag coefficients are all so small that we expect them to have no influence on the key findings of the manuscript – the complexity of rotation dynamics. Effects of drag coefficient on transition rates may possibly exist, but to estimate them depends upon the unknown details of the bearing potential and its fluctuations, and is well beyond the scope of this paper. Our EM images were recorded with a different batch of rods than that used for bearing experiments, therefore we choose not to show them. Rather readers can refer to Nanopartz literature and other sources for quantification of rod variability. We have added a discussion of these considerations to Supplementary Note S6.

#2.7

- The authors note that they excluded any cells for which the axis or rotation was close to parallel to the optical axis. They should report how many cells were excluded and whether included cells differ from the population in terms of the probe load/attachment geometry. Could the observed dynamics and heterogeneity correlate with these parameters?

We SELECTED (not excluded as the reviewer comments) nanorods with rotation axis close to the optical axis. We have updated this section to make it more clear that we select, from many candidates, those rods which have well-oriented rotation axes close to the optical axis – and the reasons for this selection. Less well-oriented rods do not allow us to recover complete and accurate orientation trajectories, so we cannot make direct comparisons. We also correct an error that may have led to confusion between rotation axis and rod angle θ. These changes are added to Methods (lines 359-67). It is not possible that this selection of well-oriented rods and rotation axes could have any effect on the observed dynamics and heterogeneity.

#2.8

- Rod drag coefficient is estimated from rods loosely attached to the surface. The rotational diffusion of such rods is expected to be affected by boundary interactions. I would expect that rods farther from the surface, such as those attached to the hook, would have a lower drag coefficient. Thus, the barrier height could be much smaller than what is estimated and reported in the summary.

This is not the case. As described in Supplementary Note 7, the Kramers’ estimate of barrier height is based on a theoretical transition time that is proportional to the drag coefficient and the exponential of the barrier height. The exponential dependence ensures that the wide variation in measured transition times corresponds to a relatively small variation in estimated barrier height. Specifically the ~140-fold range in measured times corresponds to a ~5kT range in barrier heights. Variations in drag coefficient due to surface interactions are much smaller than those measured (~10-fold), which are probably due mostly to variation in the angle between the nanorod and the rotation axis; and neither makes a large difference to the estimated barrier height.

#2.9

- Orientation reconstruction is entirely based on Fourkas' formula and has not been independently tested or calibrated. Ideally, calibration should be performed by running the procedure with a known rotating scatterer (e.g., an immobilized rod mounted on a piezo rotary stage).

We performed an angle calibration of this type, which confirms that the angle recovery is good. This is described in Extended Data Figure 3 and Supplementary Note S3. Further details are included in response to comment #1.7.

Interpretation:

#2.10

- If the bearing does have structural plasticity (line 212), how do we know that the energetics described here are the same as when a motor is driven by multiple stators?

We don't know that, and our results support no speculations on the matter. There is currently no reason to believe that the bearing dynamics would be any different when driven by torque generators, nor under any other perturbations including those suggested by this reviewer in comment #2.1. These are questions for future work.

#2.11

- How do the authors know that the motors are diffusing freely and that any stators are no longer engaged after Na+ is removed? After all, their data in Fig. 1 show a clear signature of transient activation of a stator. It is conceivable a stator that remained engaged but only generated rotation when trace sodium was available.

It is conceivable that stator complexes may remain at least transiently engaged, though current understanding of stator dynamics is that they would be largely disengaged after several/many minutes de-energized, as in our experiments. The experiments with stator proteins deleted demonstrate that the rotational dynamics are NOT affected by stator proteins, even if any do remain engaged.

#2.12

- In delta motAB M1, M3, M4 (Extended data figs 5 and 6), why do the traces show prolonged, mostly unidirectional rotation lasting ~10-100 seconds? This would be highly unexpected under passive diffusion. The authors should quantify the directional bias of rotational diffusion to test whether the motion is symmetric or preferentially CCW/CW.

We have added a reference in line 391 to theoretical estimates of optical torque in Supplementary note S5, where we report that optical torque is of order 10,000x less than the motor torque from a single stator unit. But because the drag coefficient of nanorods is so small, this is still estimated to be sufficient to rotate a free nanorod (ie NOT attached to a flagellum) at ~10 Hz. We observe such rotation, but several times slower than the theoretical estimate. We have added a figure showing measured rotation due to optical torque as a small bias to diffusion (Extended Data Fig 4a). This observed optical rotation of free nanorods is still ~50x faster (~ several revs/second) than the worst case bearing drift (Delta-motAB cell 3, ~several revs/minute). Optical torque may be our best guess for the cause of this drift, but we are not sufficiently confident of this to add it as a speculation to the manuscript. For example, the next biggest drift is in Delta-motAB cell 1, in the opposite

direction, whereas optical torque should always be in the same direction. And both of these cells drift only for part of their records. Other possible causes are even more speculative, for example unknown cellular components with an element of rotary motion relative to the flagellum. However the effect is so small compared to the rotational dynamics that we observe and analyse, that it does not alter the conclusions of the paper.

#2.13

- Lines 199-204: While a stator can generate 200 pN nm, the torque per stator unit changes with external load, therefore the torque generated by a stator and the average energy barrier is not a simple comparison. Presumably, a motor would not be experiencing zero load conditions very often, and would most commonly experience the load of a flagellar bundle. Under these conditions, one unit would not need to overcome the barriers, we expect more than one stator unit to be recruited to the motor. Additionally, stator units generate different amounts of torque depending on direction of rotation, and thus would have different interactions with the bearing's energy barrier. The statement that stator unit torque coevolved with the energy barrier of the bearing lacks evidence and requires more nuanced discussion on the function of stators.

The relevant torque for crossing an energy barrier is the stall torque, we have clarified that this is what 200 pNnm refers to. The barrier height estimate is subject to considerable uncertainties, from the uncertainties in drag coefficient discussed in response to comment #2.8 and more fundamentally from the use of Kramers theory, despite our demonstration that this theory fails to capture the full bearing dynamics. We have modified the discussion to make it more clear that the torque estimate from the barrier height estimate is not precise. Finally, we modified the wording to make it clear that we are not claiming evidence for an evolutionary link, merely observing that the two torque scales are similar. Changes are in lines 199-202.

#2.14

- In the abstract the authors note: "These findings raise the question of whether heterogeneous dynamics are a universal feature of large protein machines." It is unclear what supports this claim. I did not see a discussion in the main text linking their observations to other molecular machines, so the basis for suggesting universality should either be explained explicitly or the claim toned down.

Because this is the first ever single-molecule investigation of a large protein complex with sufficient lifetime, temporal and positional resolution to resolve and record these rich dynamics, the current evidence is that ALL such complexes that have been observed show them. Therefore it is reasonable to hypothesize that they are universal, pending further evidence.

Presentation:

#2.15

- The figures feel overly dense, with many panels and insets packed together (e.g., Fig. 2). This makes it hard to follow the data in parallel with the text. Splitting some figures into multiple, more focused ones would improve readability and help highlight key results.

We have split and enlarged the figures as recommended by the reviewer, as follows.

Old figure	New figure

1a-e	1 a-e
1f	2 a-d. Each insert becomes a separate part
2a-d	3a-d
2e-i	4a-d
3a-f	5a-e
3g-i	6a-c

#2.16

- The main text contains extensive methodological detail (e.g., lines 76–80) that interrupts the narrative flow. Moving some of this material to the Methods section would make the paper more accessible.

Lines 76-80 described the core novelties of our method, which is a key feature of the paper, and should not be taken out of the main text. However in response to the reviewer's comment, to assist the narrative flow and accessibility, we moved other experimental details, including references to supplementary materials, to the Methods section (lines 89, 290, 378, 387-93)

#2.17

- Relatedly, the writing overall is a bit dense and would benefit from editing to make the paper more approachable to a general audience. As written, the paper will be easiest to follow for the narrow intersection of scientists interested in flagellar motors and stochastic processes. Smoothing the prose and adding more explanation in some of the more technical passages would broaden the paper's reach.

In response to the 3 reviewers' comments we have edited the manuscript and expanded it beyond the word limits of the original (Nature) submission to make it more approachable to a general audience.

#2.18

- A few typographical errors were noted. When writing species names (e.g. L20, L558), the genus name is capitalized but the specific epithet should remain lowercase. In L560 "unwrapped angle f…" likely is meant to be the Greek letter phi.

Corrected.

<u>Reviewer #3 (Remarks to the Author):</u>

The manuscript by Rieu and coworkers reports on a new method to measure the rotation of the bacterial flagellar motor with unprecedented spatiotemporal resolution for angular displacements. The authors developed a high-bandwidth - spanning about seven orders of magnitude in time - polarization microscope by using gold nanorods as probes. The gold nanorods enable a fast response time and long measurement durations. Surprisingly, they found rich dynamics and heterogeneity of both active and passive motors that they convincingly attribute to subtle asymmetries in the almost rotationally symmetric structure of the motor and the different protein lattices in the flagellum. Small fluctuations and changes in structure over time may affect the overall energy landscape leading to the complex dynamics that the authors have measured. Future experiments will have to show whether other large molecular machines are subjected to similar effects and whether they

serve a biological function. The manuscript is very well written and the novel methodology can be applied to wide range of systems. I strongly recommend publication after my minor points below are addressed.

#3.1

1. Greek symbols for angles in the figure caption of Fig. 1 need to be fixed.

Corrected.

#3.2

2. Fig. 1d: In my opinion, it would be better to only show 10 ms to see more details of the 4 channels.

We have made this change.

#3.3

3. Extended Data Fig. 1: in addition to the PSD and integrated-noise plots, a plot of the angular Allan deviation as a function of averaging time would be very informative.

Done

#3.4

4. Extended Data Fig. 2: less but larger labels would be helpful.

Done

#3.5

5. Extended Data Fig. 3: please indicate the expected rotational drag coefficient based on the AuNR dimensions in the figure. Was the proximity to the surface considered? It may increase the drag coefficient somewhat (maybe 10-20 percent, see e.g. Brenner H Appl. Sci. Res. 13:81–120 (1964)).

We have added upper and lower theoretical limiting estimates of the drag coefficient to the figure (now Extended Data Figure 5), and an explanation of how these were calculated, including surface proximity, to the legend. Experiments agree well with theory.

#3.6

6. Fig. 1b: Why is there a PBS underneath the QWP? Please introduce all abbreviations in the caption (BP is missing). A more detailed schematic and list of components in particular of the three levels described in Supp. Method S1 would be helpful for the supplement (including some pictures of this implementation).

“BP” has been added to the caption (line 576). The PBS ensures that the laser illumination is linearly polarized at the QWP, and therefore circularly polarized at the sample, ensuring isotropic excitation of nanorod scattering. It also allows a second wide-field illumination path using a second laser to be added, for wide-field searching for nanorods. This has been explained along with new pictures in Supplementary Method S1.

#3.7

7. Heating effects: the authors may consider to measure the drag coefficient of surface attached AuNR as a function of excitation power for a single rod. If there is no significant change that would be a good control for heating effects.

We did this experiment and confirmed that there is no significant change. Our data set an upper limit of ~4 K to any possible heating, but indicate that the true value is likely to be much closer to zero. This experiment is added as new Extended Data Figure 4 and new Supplementary Note S4.

#3.8

8. l. 104: there is no upper left panel.

Call-out corrected (line 99).

#3.9

9. Methods: l. 325/328 fix the missing Ohm symbol

Corrected.

#3.10

10. Supplementary Note 6: The equation for Kramer's time: I presume the authors used the equation as stated - if so they should replace the proportional sign with an equal sign. Please also add the missing Boltzmann constants in the equation.

We have made these corrections and added an explanation relating our expression to the corresponding form in the original paper (new Supplementary note 7).

#3.11

11. Fig. 2a: Does the inset correspond to (3)? In d and i and Fig. 3g, the Delta is missing for the DeltaMOTAB.

Yes, the inset corresponds to (3). This has been added to the legend. Deltas are corrected.

#3.12

12. Extended Data Figure 9: Please increase the figure size. The labels and equations are difficult to read and pixelated.

Done. Now Extended Data Figure 11

#3.13

13. Extended Data Figure 10: labels for c and e are exchanged.

Done. Now Extended Data Figure 12

#3.14

14. Supplementary Note 7: The details of the model remain rather vague and the code is not provided. As stated it is not reproducible.

We have added a more comprehensive description of the model to Supplementary Information (Supplementary Note S8, Extended Data new figures 13-17).
